# Mega-dams and extreme rainfall: Disentangling the drivers of extensive impacts of a large flooding event on Amazon Forests

**DOI:** 10.1371/journal.pone.0245991

**Published:** 2021-02-12

**Authors:** Washington Luis Oliveira, Marcelo Brilhante Medeiros, Pamela Moser, Marcelo Fragomeni Simon

**Affiliations:** 1 Programa de Pós-Graduação em Ecologia, Universidade de Brasília, Brasília-DF, Brazil; 2 Embrapa Recursos Genéticos e Biotecnologia, Brasília-DF, Brazil; Areospace Information Research Institute Chinese Academy of Sciences, CHINA

## Abstract

Extreme weather events and the presence of mega-hydroelectric dams, when combined, present an emerging threat to natural habitats in the Amazon region. To understand the magnitude of these impacts, we used remote sensing data to assess forest loss in areas affected by the extreme 2014 flood in the entire Madeira River basin, the location of two mega-dams. In addition, forest plots (26 ha) were monitored between 2011 and 2015 (14,328 trees) in order to evaluate changes in tree mortality, aboveground biomass (AGB), species composition and community structure around the Jirau reservoir (distance between plots varies from 1 to 80 km). We showed that the mega-dams were the main driver of tree mortality in Madeira basin forests after the 2014 extreme flood. Forest loss in the areas surrounding the reservoirs was 56 km^2^ in Santo Antônio, 190 km^2^ in Jirau (7.4–9.2% of the forest cover before flooding), and 79.9% above that predicted in environmental impact assessments. We also show that climatic anomalies, albeit with much smaller impact than that created by the mega-dams, resulted in forest loss along different Madeira sub-basins not affected by dams (34–173 km^2^; 0.5–1.7%). The impact of flooding was greater in *várzea* and transitional forests, resulting in high rates of tree mortality (88–100%), AGB decrease (89–100%), and reduction of species richness (78–100%). Conversely, *campinarana* forests were more flood-tolerant with a slight decrease in species richness (6%) and similar AGB after flooding. Taking together satellite and field measurements, we estimate that the 2014 flood event in the Madeira basin resulted in 8.81–12.47 ∙ 10^6^ tons of dead biomass. Environmental impact studies required for environmental licensing of mega-dams by governmental agencies should consider the increasing trend of climatic anomalies and the high vulnerability of different habitats to minimize the serious impacts of dams on Amazonian biodiversity and carbon stocks.

## Introduction

The current trend of intensification of extreme climatic events, likely related to ongoing climate changes, has resulted in increased impacts on Amazon forests. A significant increase in the frequency and intensity of droughts and floods, as well as changes in the hydrological cycle, have been reported in recent years [1]. Extreme rainfall events and, consequently, large floods, have occurred more frequently in recent decades (1989, 1999, 2009, 2011 and 2012) in Amazonian rivers, generally associated with La Niña events and the rise in sea surface temperatures in the South Atlantic Ocean [2]. The occurrence of high precipitation events, alternating with periods of extreme drought, is expected to increase in the next two decades [3] as a result of global warming caused by greenhouse gas emissions of anthropogenic origin, mainly associated with increased CO_2_ concentration [4, 5]. Extreme weather events can interact in complex pathways with land-use changes, such as the allocation of hydroelectric dams and deforestation with associated fires, often leading to degradation the increasingly vulnerable ecosystems [6, 7].

Concomitant with an increased deforestation rate and climatic variability, hydroelectric dams have remarkably expanded throughout the Amazon basin [8] and plans are underway for building more than 200 new hydroelectric dams throughout the region [9, 10]. The intensification of these disturbance drivers has resulted in hydrological changes caused by the disruption of connectivity in freshwater and terrestrial ecosystems through complex feedbacks and synergistic interactions [11]. Even naturally flooded forests have shown high mortality owing to the loss of flood pulse seasonality caused by hydroelectric dams, indicating that the tolerance of riparian forest formations to seasonal floods is limited to the natural hydrological cycle of Amazonian river systems [12–14].

Among the largest hydroelectric plants in operation in the Amazon, Santo Antônio and Jirau built on the upper Madeira River started operations in mid-2011 and 2013, respectively, after a controversial licensing process [15]. During the 2013/2014 rainy season, extreme precipitation anomalies occurred in southwestern Amazonia, exceeding the region’s average values by up to 100% [16] and resulting in historic levels of rainfall and discharge in the Madeira River [17]. As a consequence, extensive flooding in the Madeira River basin occurred, mainly in the area affected by the Santo Antônio and Jirau dams. During this event, the estimated flooding in the area, likely influenced by the hydroelectric dams, reached more than 800 km^2^, or 64.5% greater than that predicted in the environmental impact assessments of these hydroelectric dams, causing extensive environmental and social losses, including increased tree mortality [17, 18]. However, it is still unclear if extreme flooding in the upper Madeira River basin was caused primarily by abnormal precipitation, or whether flooding was amplified in an exceptional way by the presence of the Jirau and Santo Antônio mega-dams, with precipitation acting as a secondary factor in this process. Understanding the causes of such impacts has broad implications for the future of natural ecosystems in that region, quite apart from relevant social and economic aspects, which are beyond the scope of this study.

Aiming at understanding the ecological impacts of the presence of a mega-dam on the surrounding vegetation, several studies have been carried out in the area around the Jirau reservoir, where a network of permanent forest plots was established. Plot survey showed that composition and abundance of tree species were mainly determined by soil fertility and the distance from the nearest drainage, reflecting the influence of the riparian zone on the vegetation [19]. It also has been shown that 61% of palm species recorded in the study area occur within the area of direct influence predicted by the reservoir [20]. Environmental heterogeneity is a key factor for maintaining beta-diversity patterns among different vegetation types, with shallower water table during the rainy season representing a strong environmental filter for *campinarana* forest communities [21]. Tree mortality in campinarana forests affected by the 2014 flood resulted in consistent shifts in community function and a sharp reduction in the abundance of flood-sensitive species [17]. Despite the advances achieved by these studies, it is not clear how the 2014 flooding affected different vegetation types in the area around the Jirau dam, and its impacts on tree mortality, forest biomass, and species composition.

The main goal of this study was to access the impacts of the 2014 flood on the forests of the Madeira basin, and investigate the role of mega-dams as drivers of forest mortality. This goal is achieved through two complementary approaches. First, we used satellite imagery data to investigate the patterns of forest loss along the Madeira basin, including its main tributaries, after the 2014 flood. We hypothesize that the presence of mega-dams in the Madeira River basin played a major role in the magnitude of the 2014 extreme flood, amplifying the impacts of abnormal rainfall recorded for the 2013–2014 period in southwestern Amazonia. To test this hypothesis, we compared estimates of forest loss between areas directly affected by hydroelectric dams and areas not affected by dams along the Madeira basin. Secondly, we evaluated on a more detailed spatial scale, the impact of extreme flooding on tree mortality, aboveground biomass and species composition in monitored plots allocated in different habitats under the influence of the Jirau hydroelectric dam. Plots were surveyed before (2011) and after (2015) the extreme flooding and included areas both affect and not affected by inundation. We hypothesize that forest plots placed in habitats naturally subject to seasonal rise of the water table such as *várzea* and *campinarana* forests would be less affected by the extreme flood compared to forests growing on well-drained terrain. The outputs of these analyses are critical considering the extensive environmental impacts of a combination of river dams and extreme climatic events, which are not foreseen in environmental studies required to assess impacts of mega-dams, but are expected to be more frequent in a period of ongoing climate change.

## Material and methods

### Study area

The Madeira is considered the fifth largest river in the world and the largest tributary of the Amazon River in water discharge, contributing approximately 15% of its total net water discharge [22]. The discharge in Porto Velho (the capital city of the Brazilian state of Rondônia and where the Santo Antônio hydroelectric dam is located) ranges from 5,000–6,000 m^3^/s in the dry season to 45,000–50,000 m^3^/s in the rainy season, with an annual average of 23,000 m^3^/s. The Madeira River carries a large amount of suspended material, and, as such, it is responsible for the largest sediment discharge in the Amazon basin, representing around 50% of the sediment load transported by the Amazon River [11]. The Madeira River basin occupies an area of 1.42 M km^2^ [23] with a hyperthermic humid tropical climate [24]. Minimum and maximum annual average temperatures are 21.1°C and 32.2°C, respectively, with an annual average relative air humidity of 79% [25]. Annual average precipitation varies between 1,700 and 2,200 mm [26]. The hydrological cycle of the upper Madeira region is typically seasonal, varying according to the prevailing climatic season: discharge (May to August); low water level period (September and October); water level rising (November to February) and high water peak (March and April), some two months earlier than the Amazon River [27]. The Madeira basin harbors a large variety of aquatic and terrestrial habitats that are home to extensive biodiversity, as well as riverine people, with fundamental dependence on natural resources [28].

After an energy crisis in the beginning of the 2000’s, Brazil resumed its old hydroelectric projects in the Amazon, which included two mega-dams in the Madeira River (Santo Antônio and Jirau). The project for the Madeira River hydroelectric complex was boosted with the expectation of implementing small reservoirs with a high potential for energy use, compared to other dams previously installed in the Amazon region [15, 29, 30]. These mega-dams were designed as ‘run-of-the-river’ hydroelectrics that require little water storage and have a small reservoir size compared to traditional hydroelectric dams. Their operation involves both permanent and temporary flooding in forest areas surrounding the reservoirs as a result of seasonal rise in the river level. The Santo Antônio dam was built near Porto Velho, while the Jirau dam is located approximately 100 km upstream (S1 Fig), with the final filling phase of the reservoirs starting on September 2011 and November 2013, respectively.

### Variation in rainfall and Madeira River level during the extreme 2014 flood

**Fig 1:**
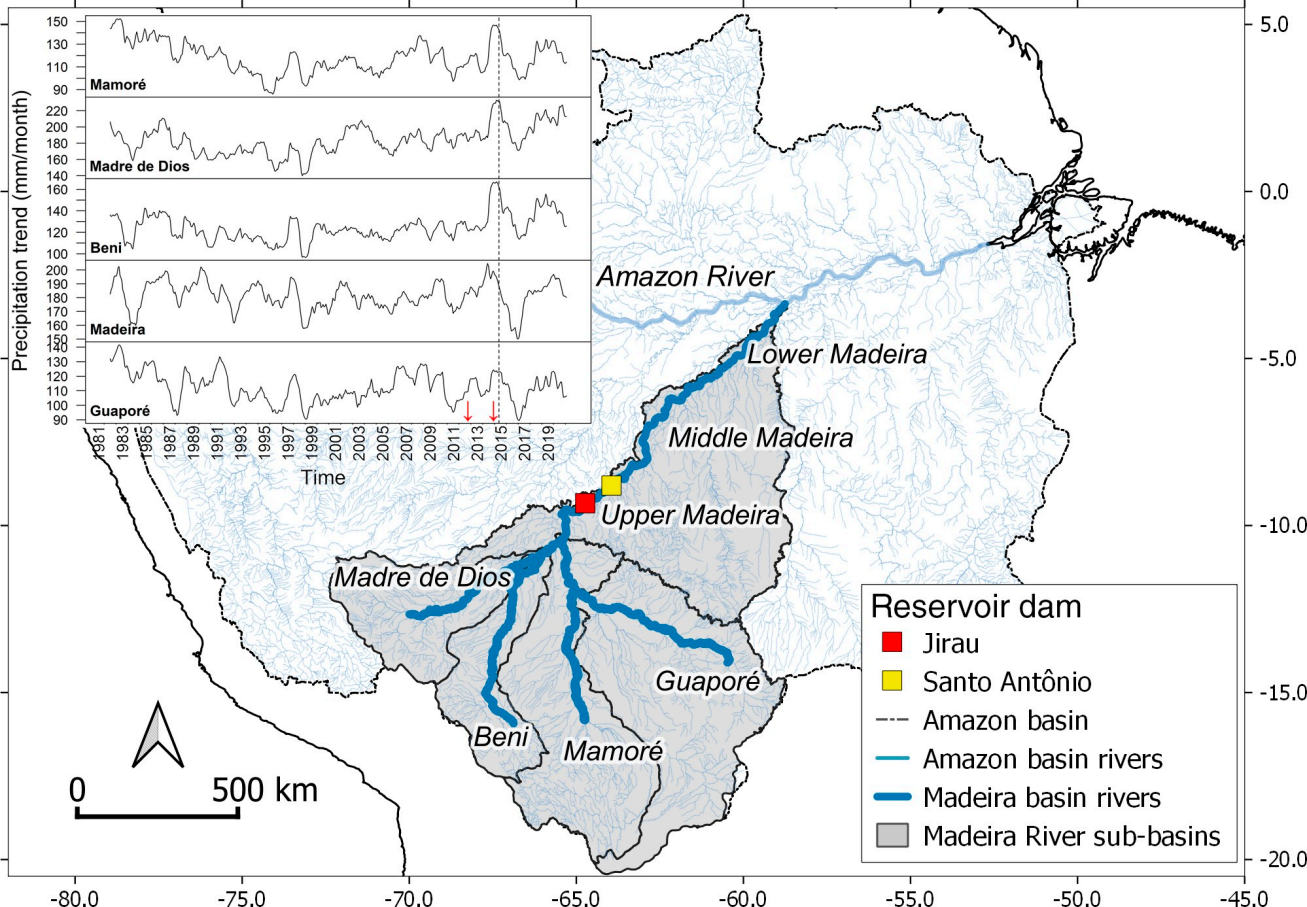
Study area in the Amazon basin highlighting the hydrographic basins of the Madeira River and its main tributaries, as well as the location of Jirau and Santo Antônio mega-dams. The inset on the map shows the precipitation trend in each sub-basin (upper, middle and lower sections of the Madeira River were grouped) based on data from the global rainfall dataset CHIRPS [31]. Red arrows represent the dates of the filling of Santo Antônio (September 2011) and Jirau (November 2013) reservoirs; the vertical dotted line indicates the peak of flooding at Porto Velho-RO, Brazil (April 2014). The limit of the Amazon basin and the hydrographic network were obtained from [32] and are openly shared datasets, without restriction, in accordance with the EOSDIS Data Use Policy, and the coastline was provided by The World Bank with data licensed by CC-BY4.0.

The last stage of the filling of the Jirau reservoir started at the end of 2013, a period that coincided with rainfall anomalies in southwestern Amazonia and a historic record in Madeira River discharge. We investigated extreme precipitation anomalies that occurred in southwestern Amazonia during the 2013–2014 rainy season, particularly in association with extensive flooding in the region of the Madeira basin, which was grouped into sub-basins: Madre de Dios, Beni, Mamoré, Guaporé, and Madeira, the latter including the upper, middle and lower sections of the Madeira River (Fig 1). Monthly average rainfall in the five sub-basins was obtained from CHIRPS (Climate Hazards group Infrared Precipitation with Stations), a global rainfall dataset [31] based on rain gauge and satellite observations at high spatial resolution of 0.05 x 0.05 degrees (https://www.chc.ucsb.edu/data/chirps). Rainfall anomalies from September 2013 to August 2014 were compared with monthly average estimates from January 1981 to August 2020 in each sub-basin of the Madeira River.

We also investigated the variation of water level in a stretch of the Madeira River critically affected by the 2014 flood by using a time series (November 2008 to February 2018) of the limnimetric scale readings from four locations in the area of influence of the Jirau dam. We used this ten-year dataset to investigate the occurrence of significant extreme events in the hydrological time series using tools available in the Hydrological Data Discovery Tools ‘*hddtools*’ package [33] for R software [34].

### Flooding and forest loss along the Madeira River basin

We assessed the extent of flooding and forest loss along the Madeira basin after the 2014 flooding. We compared flooding and forest loss rates in areas under the influence of the Jirau and Santo Antônio dams, as well as in river stretches outside the area of influence of these dams, both upstream and downstream. We included in this analysis the Madeira River and its main tributaries: Madre de Dios, Beni, and Mamoré, all from the Andes, and Guaporé, with springs originating in the Brazilian crystalline shield. River courses were obtained as shapefiles provided by [32]. A 6-km-wide buffer, which encompasses the most affected areas by the 2014 extreme flood, was defined perpendicular to the river course and variable in length, according to sub-basin river size. The same procedure was done for the Jirau and Santo Antônio reservoirs, which were divided into initial (dam), middle and final sections.

To compare the extent of flooded area during the 2014 extreme event between sub-basins, we used data from the long-term global surface water mapping data at a resolution of 30 meters featuring changes in seasonality and surface water cover across a historical series from 1984 to 2015 [35]. We used this product to evaluate the intensity and change in the occurrence of surface water based on records of water presence in each month over the years. We used the Water Occurrence Change Intensity tool (parameter CHANGE), which discriminates between ’permanent’ and ‘maximum water surfaces’ considering the inter-annual water distribution [35]. Permanent water was extracted from the data considering pixels that did not show any seasonal change over the time series (CHANGE = 100). The extension of flooding during the extreme 2014 flood was estimated from the maximum level delimited by the peak of the visible area of water surface. Estimates of permanent water and flooded area within the 6-km buffer were calculated for each sub-basin of the Madeira River, as well as for the areas affected by the Jirau and Santo Antônio dams. We acknowledge that these estimates of flooded area at the peak of the 2014 flood can be biased due to the low frequency of satellite imaging (every 16-days), as well as noise caused by clouds. As the peak of the flood occurred in the beginning of the dry season (April 2014), we assume that cloud cover did not biased our estimates of flooded area. In addition, these measurements do not capture flooding below forest canopy, and therefore underestimate the extent of flooding. Despite these limitations, we believe that these estimates can provide suitable data for a comparative analysis between the sub-basins considered here.

To estimate forest loss after the 2014 flood, we used data from Global Forest Change [36] at a spatial resolution of 30 meters. Areas delimited by buffers of 6 km along rivers were used as masks to extract forest loss data, and the resulting scene was converted to the respective UTM zone for area calculation. Forest loss caused by the 2014 flood was calculated for each sub-basin and areas affected by dams as the sum of forest loss recorded in 2014 and 2015, considering that the effects of flooding on tree mortality could appear one year after flooding. Forest loss not related to flooding (e.g., deforestation) was excluded from the analyses. Visual inspection of Landsat images allowed us to identify features associated with logging, pastures or agriculture (eg. forest clearings of regular shape, generally along roads and far from the river; S1 Fig), which were removed from our calculations of forest loss since they are not related to flooding. Forest loss associated with the filling of the Jirau reservoir, predicted to operate at 90 m level, was not considered here since forest loss in this case is not related with the 2014 flood.

### Impacts of flooding on different habitats along the Jirau reservoir

In addition to the regional scale study on the impact of flooding on forest loss in the Madeira River basin, we carried out a detailed study with a network of permanent forest plots in the area affected by the Jirau reservoir, one of the areas most damaged by the 2014 flood. These forest plots are part of the “Programa de Conservação da Flora da UHE Jirau” that aims to understand the impact of the Jirau dam on the different forest formations around the reservoir. Data were sampled before (2011) and after (2015) the Jirau reservoir filling. Following the RAPELD protocol [37], we allocated 26 permanent plots (1 ha-plots) distributed into eight transections perpendicular to the river established in three modules corresponding to initial (Caiçara), middle (Mutum) and final (Abunã) sections of the Jirau reservoir, (S1 Fig; [19]). Among the 26 sampled plots, 17 were affected by the 2014 flooding.

The data were collected in three sub-plots per plot, according to classes of DBH (diameter at breast height). In a 250 x 2 m (0.05 ha) sub-plot, all individuals with 1 cm ≤ DBH < 10 cm were sampled; in a 250 x 20 m (0.5 ha) sub-plot, all individuals with 10 cm ≤ DBH < 30 cm were sampled; and individuals with DBH ≥30 cm were sampled in a 250 x 40 m (1 ha) sub-plot. We collected voucher specimens of at least one individual of each morphospecies surveyed for further identification and deposit at the Embrapa Genetic Resources and Biotechnology Herbarium (CEN).

The study area comprises a mosaic of vegetation types mainly determined by water table saturation and soil fertility, and were classified into four major habitats: *terra firme* forests, transitional forests, *várzea* forests, and *campinarana* forest [21, 38] (S1 Fig). *Terra firme* forests occur on well-drained terrain and low-fertility soils, and predominate in the upland areas in the north margin of the Madeira river (seven plots sampled), comprising areas with highest species richness and diversity [21]. Transitional forests comprise sites on well-drained soils with intermediate fertility that have been subject to occasional disturbances and include areas with high incidence of bamboo (two plots) and “*sororoca*” (*Phenakospermum guyannense*; four plots). As a consequence, these areas have a different species composition and lower diversity compared to *terra firme* forests [21]. The vegetation that occur in the riparian zone along the Madeira river margins has a markedly distinct structure and floristic composition compared to adjacent upland habitats away from the river [19, 21], and is characterized by high-fertility soils and a shallow water table. Because of Madeira river’s deep catchment and high margins, seasonal natural flooding is restricted to a narrow strip of land on its banks, contrasting with highly flooded areas in most riparian zones along large Amazon basin rivers [39]. Although not directly corresponding to the vegetation typically found in the Amazonian floodplains, this habitat (six plots sampled) was referred here as *várzea* forests, and corresponds to an intermediate habitat between typical “*high-várzea*” [39] and *terra firme* forests. *Campinarana* forests, locally known as “*umirizal*”, correspond to low stature, species-poor forests that occur on flat lowland areas along the southern bank of the Madeira river (seven plots sampled), and are subject to periodic flooding due to elevation of ground water table during wet season. Soils generally have high silt content and intermediate fertility, which contrasts with white sand soils found in other Amazonian *campinarana* areas. The four major habitats sampled here are well differentiated in terms of water-table variation and edaphic attributes and have distinct structure and species composition. Detailed information on vegetation types found in the study area are presented in [21, 38]. Given their different exposure to natural flooding and levels of sensitivity to water stress, we expect that plots sampled in the four habitats surveyed would be affected differently by various levels flood intensity caused by the filling of the Jirau dam. More specifically, we expect that plots located in non-flooded terrain (*terra firme* and transitional forests) will be more sensitive to flooding than plots located in habitats naturally subject to seasonal flooding such as *várzea* and *campinarana* forests.

#### Flood intensity in forest sampling plots

We calculated the flood index (FI) in each plot as the number of consecutive days that a plot remained flooded (including the water table at ground level) by the rise of the Madeira river. To estimate the magnitude of the aboveground flood, we adjusted the variation in ground water height in each plot according to the time series of limnimetric scale readings from four locations along the Jirau reservoir (S1 Fig) and calibrated for the altitude of each plot and the maximum height mark of the flood measured on the tree trunks during the 2014 and 2015 flood seasons. Plot altitude was obtained discounting mean tree height per plot from the digital surface model with 5 m pixels, as obtained by aerial survey with laser sensor and three points.m^-2^ (data provided by Energia Sustentável do Brasil, ESBR).

#### Change in forest structure and species composition after the 2014 flood

We used exponential models to estimate annual average tree mortality and variation in aboveground biomass (AGB) in each plot based on censuses before and after damming [40]. The AGB estimate was obtained with the allometric model: AGB = 0.0673 x (ρD^2^H) 0.976, where D is the diameter in cm, H is the height in m, and ρ is the wood density in g cm^-3^ [41]. Diameter and height were obtained from plots census, and wood density was obtained from a functional database for tropical trees [42]. Wood density average values for the genus were adopted when the species sampled was not included in the database, or when the individual was identified only at the genus level. We also used family average wood densities for trees identified at the family level, and global average wood density of the inventory for trees that remained undetermined. The number of individuals determined at the family or genus level or undetermined was relatively small (10.7%). The abundance estimates and AGB were standardized for area (hectare) by extrapolating the values calculated in the sub-plots.

To characterize variation in plot species composition, we used the Sørensen index (presence/absence dataset) and the Bray-Curtis index (abundance dataset), with species abundances standardized by the total number of individuals per plot. Dissimilarities among 26 plots were summarized by a non-metric multidimensional scaling analysis (NMDS) [43] using the vegan package [44]. This analysis enabled a comparison of species composition between and within plots, considering measurements taken before (2011) and after (2015) the 2014 flood.

We examined the effects of FI per habitat type on individual tree mortality rate between 2011 and 2015 by using a generalized linear mixed model (GLMM). In order to control for random variation in each plot, we used binomial distribution and maximum likelihood adjustment, as well as Laplace approximation by using the *lme4* package [45]. Plot responses to flooding were evaluated by using mixed linear effects models, with plot identity as a random effect nested in the transections and sample modules, in order to consider the bias of repeated measures over time, before and after flooding, besides the spatial autocorrelation between sample units. We tested the effects of FI and habitat predictor variables on the following response variables: live AGB; dead AGB, which included both dead trees that had fallen and also dead trees that remained standing; absolute change in live aboveground biomass measured before (2011) and after (2015) the 2014 flood; and the Fisher’s alpha diversity index. Random components were evaluated from the saturated model with all variables and interaction between FI and habitat, adjusted with maximum restricted likelihood and selected using the Akaike information criterion (AIC) [46]. Then, the fixed predictors were selected with the maximum likelihood test, according to the protocols established in the literature [47], using the “*nlme*” package [48]. All analyses were performed in R software [34].

## Results

### Rainfall anomaly in southwestern Amazon and variation in Madeira River water level

**Fig 2:**
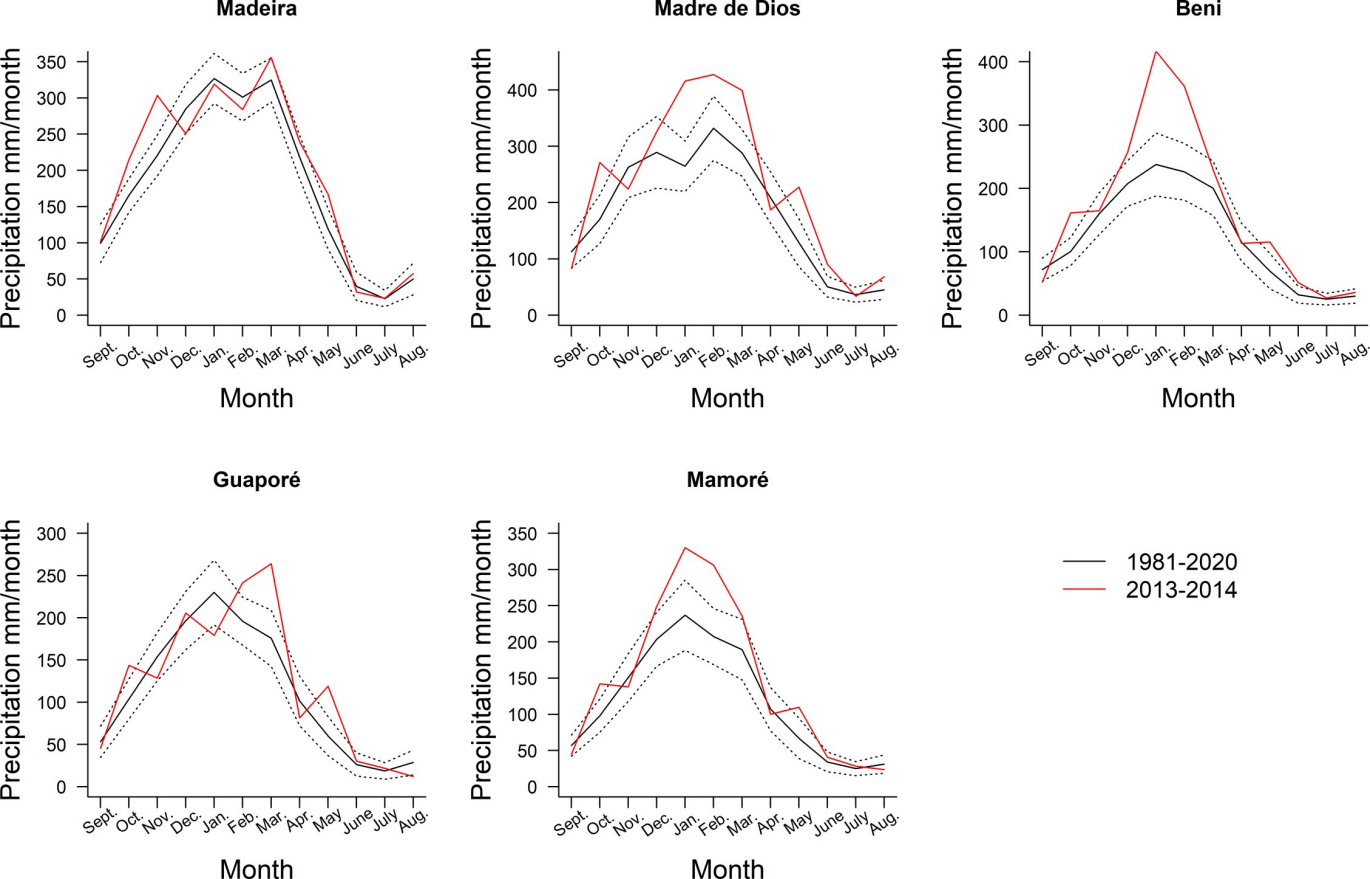
**Monthly average rainfall (black line) ± standard deviation (dotted line) on Madeira River Sub-basins between September/1981-August/2019.** Monthly rainfall associated with the extreme 2014 flood period from September/2013 to August/2014 is denoted by the red line, in the hydrographic basins of the Madre de Dios, Beni, Mamoré, Guaporé and Madeira Rivers. Data source: CHIRPS v2.0 [1, 31] (https://www.chc.ucsb.edu/data/chirps).

**Fig 3:**
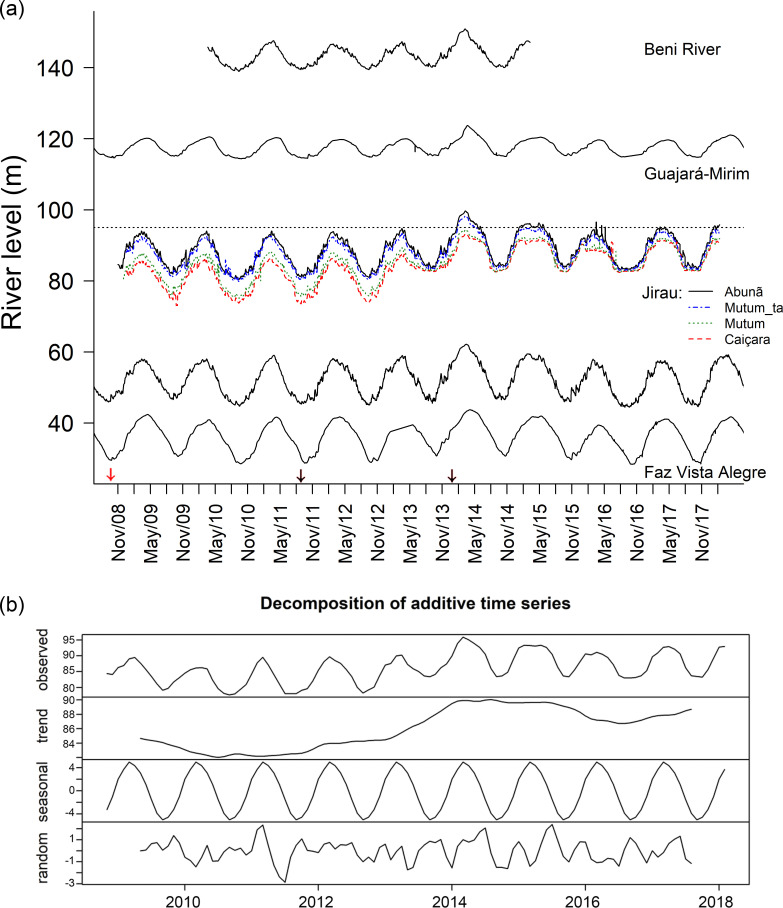
Water level recorded in different portions of the Madeira River between 2008–2019. (a) Beni River = UHE Jirau Jusante Rio Beni, station # 15318000 (130 m altitude); Guajará-Mirim, station # 15250000 (109.3 m); Jirau stations: initial (Caiçara), middle (Mutum Tamborete, Mutum) and final (Abunã) sections of the Jirau reservoir before (2008–2013) and after reservoir filling (2013–2018); Porto Velho, station # 15400000 (42.5 m); Faz Vista Alegre = Fazenda Vista Alegre, station # 15860000 (20 m). (b) One-decade time series additive decomposition observations of the Madeira River water level (2008–2018) in the initial section of the Jirau reservoir (Caiçara), indicating estimated trends, seasonal pattern and time series random component. The red arrow indicates the start of construction of the hydroelectric dams on the Madeira River (September 2008) and the black arrows indicate the final stages of filling the Santo Antônio (September 2011) and Jirau (November 2013) reservoirs. Data provided by ANA at the HidroWeb Portal (http://www.snirh.gov.br/hidrotelemetria); Jirau data provided by ESBR (Energia Sustentável do Brasil).

The extreme 2014 flood in the upper Madeira basin was preceded by rainfall anomalies in late 2013 and early 2014 in its tributaries. High precipitation occurred in the watersheds of Mamoré, Madre de Dios, Beni, with rainfall peaks far above the 1981–2020 average (Fig 2, S1 Table). Precipitation in the rainiest month was 75% higher than the historical average in the Beni basin, 61% higher in the Madre de Dios basin, 39% higher in the Mamoré basin, 15% higher in the Guaporé and 9% higher in the Madeira basin (Fig 2). Annual rainfall between September 2013 and August 2014 was higher than the historical trend in all basins, respectively, 34%, 26%, 24%, 10% and 8%. The level of the Madeira River measured in four locations in the area of influence of the Jirau dam reached its peak in April 2014 (Fig 3A). After reaching this historic maximum, the level of the river remained higher than average, but with reduced amplitude as a consequence of the operation of the Jirau dam (Fig 3B).

### Extreme 2014 flood and forest loss

**Fig 4:**
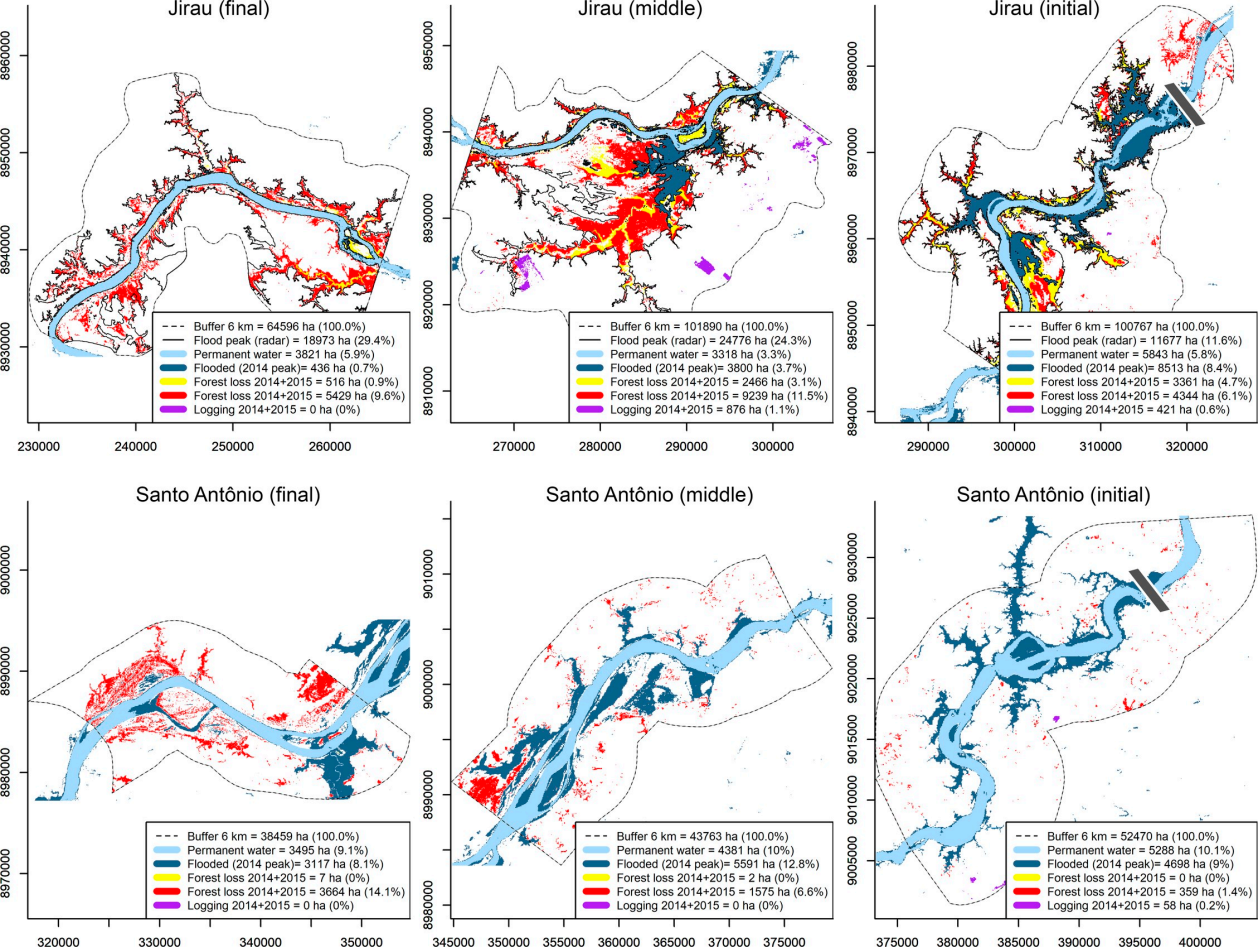
Landscape sections in the area affected by the Jirau and Santo Antônio reservoirs (final, middle and initial sections), the two mega-dams on the Madeira River. Effects of the 2014 extreme flood are indicated. Diagrams show the 6-km-wide buffer (Buffer) on each bank along the course of the river, percentage of permanent surface water (Permanent water), flooded area at the peak of 2014 extreme flood (Flooded) visible by optical sensors (Landsat), flooded area peak along the Jirau reservoir estimated by radar sensor is denoted by the black line (data provided by Energia Sustentável do Brasil, ESBR) is denoted by black line, forest loss two years after flooding (2014+2015), highlighting forest loss from filling the reservoirs (yellow) and the loss of forest of unforeseen areas beyond the predicted limits of the reservoirs (red). Deforestation by logging, which was not considered in the calculations of forest loss caused by flood, are shown in purple. Percentage of forest loss is relative to the total area of forest cover in 2013 before flooding. Location of dams are indicated by grey bars. Additional analyzed buffers are presented in S1 Appendix. Permanent superficial water and flooded area data from EC JRC/Google [35]. Forest loss data from Hansen/UMD/Google/USGS/NASA [36].

The proportion of permanent waters, which do not vary seasonally, was higher in the course of the Madeira River (11.8%), followed by Mamoré (5.9%) and Madre de Dios (4.2%) (Table 1). The 2014 flood detected by satellite imagery reached an estimated 3062 km^2^ of flooded area in the entire Madeira basin, including its tributaries and the area flooded by hydroelectric reservoirs, as measured in 6-km-wide buffers. The Mamoré, Beni and Madeira Rivers had at least, respectively, 1131, 728 and 476 km^2^ of flooded area, equivalent to 11.4%, 6.2% and 3.5% within each of the 6km buffers, while Jirau and Santo Antônio had 4.8% and 10% of their area affected by flooding, respectively. Rates of forest loss owing to the 2014 extreme flood were higher in the areas affected by the Jirau (9.2%) and Santo Antônio (7.4%) reservoirs when compared to areas outside the influence of these hydroelectric dams (0.5–1.7%; Table 1). Flooding that exceeded the top level of the reservoir in unforeseen areas resulted in forest loss of 4344, 9239 and 5429 ha at the initial, middle and final sections of the Jirau reservoir, respectively, and 359, 1575 and 3664 ha in the initial, middle and final sections of the Santo Antônio reservoir (Fig 4). The overlap between the maximum flooded area in 2014 and forest loss was negligible, corresponding to only 120 ha in Jirau and 52 ha in Santo Antônio, and representing less than 0.02% of the buffer area in the sub-basins analysed. Forest loss not related to flooding (e.g., deforestation by logging) comprised 1297 ha in Jirau, 58 ha in Santo Antônio, and 513 ha in the upper Madeira River, and was negligible in other sub-basins analyzed. Maps describing the detailed landscape parameters for each sub-basin are presented in S1 Fig and S2 Table.

**Table 1:** Landscape parameters in the sub-basins Beni, Guaporé, Madre de Dios, Mamoré, and Madeira, the latter including the upper, middle and lower sections of the Madeira River and excluding the areas of influence of the Jirau and Santo Antônio reservoirs, which are presented separately.

River Basin	Buffer	Perennial	Flooded	Forest 2013	Forest Loss
**Beni**	11675.05	231.87 (2.0%)	728.20 (6.2%)	9486.53 (81.3%)	156.76 (1.7%)
**Guaporé**	9058.10	196.84 (2.2%)	246.58 (2.7%)	7142.69 (78.9%)	33.73 (0.5%)
**Madre de Dios**	7696.41	319.85 (4.2%)	218.69 (2.8%)	6741.39 (87.6%)	50.00 (0.7%)
**Mamoré**	9910.02	585.62 (5.9%)	1130.68 (11.4%)	6055.59 (61.1%)	87.00 (1.4%)
**Madeira**	13636.43	1614.07 (11.8%)	476.47 (3.5%)	10575.80 (77.6%)	173.18 (1.3%)
**Jirau**	2672.53	129.82 (4.9%)	127.49 (4.8%)	2071.09 (77.5%)	190.12 (9.2%)
**Sto Antônio**	1346.92	131.64 (9.8%)	134.06 (10%)	752.62 (55.9%)	55.98 (7.4%)

Area (km^2^) occupied by different features within 6-km-wide buffers established on each bank along river channels (Buffer); permanent surface water area without seasonal variation (Perennial) from long-term global surface water mapping data; visible flooded area estimated to 2014 extreme flooding (Flooded); total area of forest cover in 2013 before flooding (Forest 2013); summed forest loss in 2014 and 2015 (Forest Loss). Calculation of forest loss in the reservoirs of Jirau and Santo Antônio excluded areas submerged by the filling of reservoirs. Therefore, only areas beyond the hydroelectric reservoirs were considered as forest loss. Percentage values in parentheses show the fraction of each metric (Perennial, Flooded, Forest 2013) in relation to the total area of the respective buffer and the percentage of Forest Loss is relative to the total area of forest cover in 2013 before flooding.

### Impacts of the 2014 flood on different habitats in the area affected by the Jirau dam

**Fig 5:**
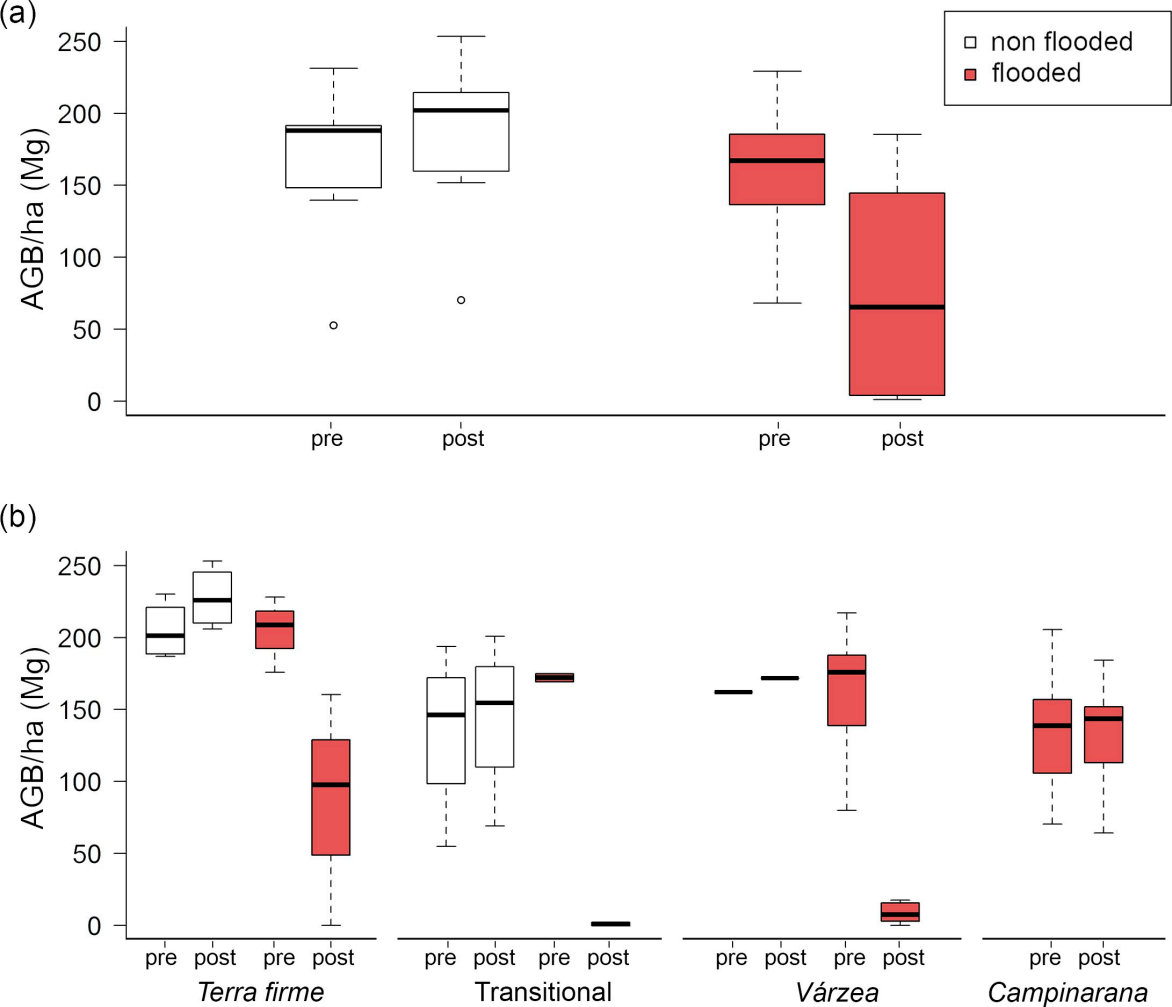
Aboveground biomass (AGB) per hectare in 26 forest plots sampled before (2011; pre) and after (2015; post) the filling of the Jirau reservoir in 2014. (a) AGB estimates of non-flooded (N = 9) and flooded (N = 17) plots sampled pre- and post-dam. (b) AGB estimates in different habitats: *terra firme* forests (non-flooded N = 4; flooded N = 3), transitional forests (non-flooded N = 4; flooded N = 2), *várzea* forests (non-flooded N = 1; flooded N = 5) and *campinarana* forests (flooded N = 7), sampled pre- and post-dam.

Among the 26 forest plots monitored in the Jirau dam area, 17 were simultaneously affected by the filling of the Jirau reservoir and the effects of the 2014 flood. Four out of five *várzea* forest plots, as well as three *terra firme* and two transitional forest plots, were severely affected by flooding (FI>100), which resulted in substantial reduction in tree abundance, AGB, and species richness (Table 2). Three plots underwent 100% of tree mortality (one *várzea*, one transitional forest and one *terra firme* forest plot). On the other hand, all seven plots of *campinarana* forests were reached by the 2014 flood (FI≥50), but this habitat was more resilient than others, showing limited changes after extreme flooding (Table 2). Most plots not affected by flooding showed a modest increase in surveyed parameters (Table 2). Additional structural and compositional parameters of flooded and non-flooded plots are provided in S4 and S5 Tables. Average AGB (± sd) of plots not affected by flood was estimated at 167.3 ± 52.6 tons/ha in 2011, with a 10.2% increase in biomass (184.3 ± 55.0 tons/ha) in 2015. On the other hand, plots affected by flood suffered a 49.0% reduction in AGB, from 155.8 ± 48.0 tons/ha in 2011 to 72.1±70.0 tons/ha in 2015 (Fig 5A). *Terra firme* forests that were not flooded increased their AGB by 10.8%, while flooded plots showed a reduction of 57.9% in AGB, from 204.3 ± 26.4 tons/ha to 86.0 ± 80.8 tons/ha (Fig 5B). The greatest impact occurred in flooded transitional forests with 99.2% reduction of AGB in contrast to the average biomass increase of 9.8% of plots of this same habitat that did not suffer flooding. *Várzea* forests also showed a large AGB decrease from 156.5 ± 52.8 tons/ha before flooding to 8.7 ± 7.7 tons/ha after flooding (94% decrease). On average, *campinarana* forests showed a slight increase in AGB, going from 130.9 ± 45.9 tons/ha before flooding to 131.7 ± 39.7 tons/ha after flooding. Similarly, flooding affected all vegetation strata, including understory (1 ≤ DBH <10 cm), canopy (10 ≤ DBH <30 cm), and emergent trees (DBH> 30 cm), all showing a sharp decrease in AGB, except for *campinarana* trees, which remained largely unaltered (S2 Fig; S3 Table).

**Table 2:** Tree monitoring plots in the initial, middle, and final Jirau reservoir sections (Caiçara, Mutum and Abunã, respectively).

Location	Plot	Habitat	Impact	Flood index			% change: 2015/2011		
				2012	2014	2015	N	AGB	S
Caiçara	T1-P1*	TF	F	0	187	224	-100.00	-100.00	-100.00
Caiçara	T1-P2**	TF	F	0	121	189	-73.28	-56.38	-69.71
Caiçara	T1-P3	TF	NF	0	0	0	0.26	6.99	3.93
Abunã	T10-P2**	TF	F	0	134	98	-32.94	-4.89	-41.32
Abunã	T11-P2	TF	NF	0	0	0	-3.39	5.92	-1.16
Abunã	T11-P4	TF	NF	0	0	0	-3.70	9.01	3.17
Abunã	T11-P5	TF	NF	0	0	0	2.26	7.24	8.67
Caiçara	T1-P4	DF	NF	0	0	0	-2.44	2.05	-0.69
Caiçara	T1-P5	DF	NF	0	0	0	-8.18	6.61	1.39
Caiçara	T4-P2*	DF	F	0	149	155	-97.48	-98.64	-91.60
Caiçara	T4-P3*	DF	F	0	125	48	-100.00	-100.00	-100.00
Caiçara	T4-P4	DF	NF	0	0	0	-11.84	24.21	-1.22
Caiçara	T4-P5	DF	NF	0	0	0	-5.19	11.29	6.09
Mutum	T5-P1*	VF	F	0	159	184	-90.77	-96.42	-89.23
Mutum	T6-P1*	VF	F	28	152	115	-100.00	-100.00	-100.00
Mutum	T7-P1*	VF	F	0	172	203	-97.48	-98.72	-96.58
Mutum	T8-P1*	VF	F	7	132	204	-88.41	-89.27	-91.96
Abunã	T10-P1**	VF	F	0	113	51	-89.11	-89.58	-78.13
Abunã	T11-P1	VF	NF	0	0	0	-6.35	5.93	3.53
Mutum	T6-P2	CF	F	0	144	129	-22.06	14.38	-36.96
Mutum	T6-P3	CF	F	0	134	143	-30.63	-0.85	-34.25
Mutum	T8-P2	CF	F	0	50	0	-16.67	7.59	-2.27
Mutum	T8-P3	CF	F	0	108	20	-28.26	-8.27	-29.58
Abunã	T10-P3	CF	F	0	60	60	-25.64	7.42	-6.82
Abunã	T10-P4	CF	F	0	69	0	-19.40	20.86	-2.17
Abunã	T10-P5	CF	F	1	83	0	-16.74	13.58	-3.41

Habitats (TF = *terra firme* forests, DF = transitional forests, VF = *várzea* forests, CF = *campinarana* forests). Impact: 2014 flooded (F) and non-flooded (NF) plots. Flood Index is the number of consecutive days that a plot was flooded by the waters of the Madeira River before (2012) and after the Jirau reservoir filling (2014–2015). Relative change in values recorded in 2011 and 2015 is presented for the following parameters: abundance of arboreal individuals (N), aboveground biomass (AGB) and species richness (S). Plots are sorted by habitat and plot name. Plots were classified as located within areas of forest loss (*), and on the edge of forest loss areas (**), as detected by the analysis of Global Forest Change data at a spatial resolution of 30 meters.

In *terra firme* and transitional forests affected by flood, we found a slight increase in average wood density, suggesting higher mortality of softwood species. On the other hand, in *várzea* forests, average wood density per individual decreased 60% (S4 Table), reflecting high tree mortality after flooding and subsequent colonization by two fast-growing pioneer species (*Cecropia ficifolia* and *Muntingia calabura*). Wood density remained unchanged in plots not affected by flood, and also in *campinarana* forests (S5 Table).

Tree mortality in flooded plots resulted in a sharp reduction in composition parameters, such as number of singletons and doubletons, species, genera and families per plot (S4 Table). Mean number of singletons per plot decreased by 63.0%, 92.2% and 98.6% in flooded *terra firme*, transitional, and *várzea* forests, respectively (S4 Table). Flooded plots also experienced a significant reduction in species richness with loss of 69.3% of morphospecies present before flooding in *terra firme*, 95.0% in transitional, 86.8% in *várzea*, and 6.1% in *campinarana* forests (S4 Table).

**Fig 6:**
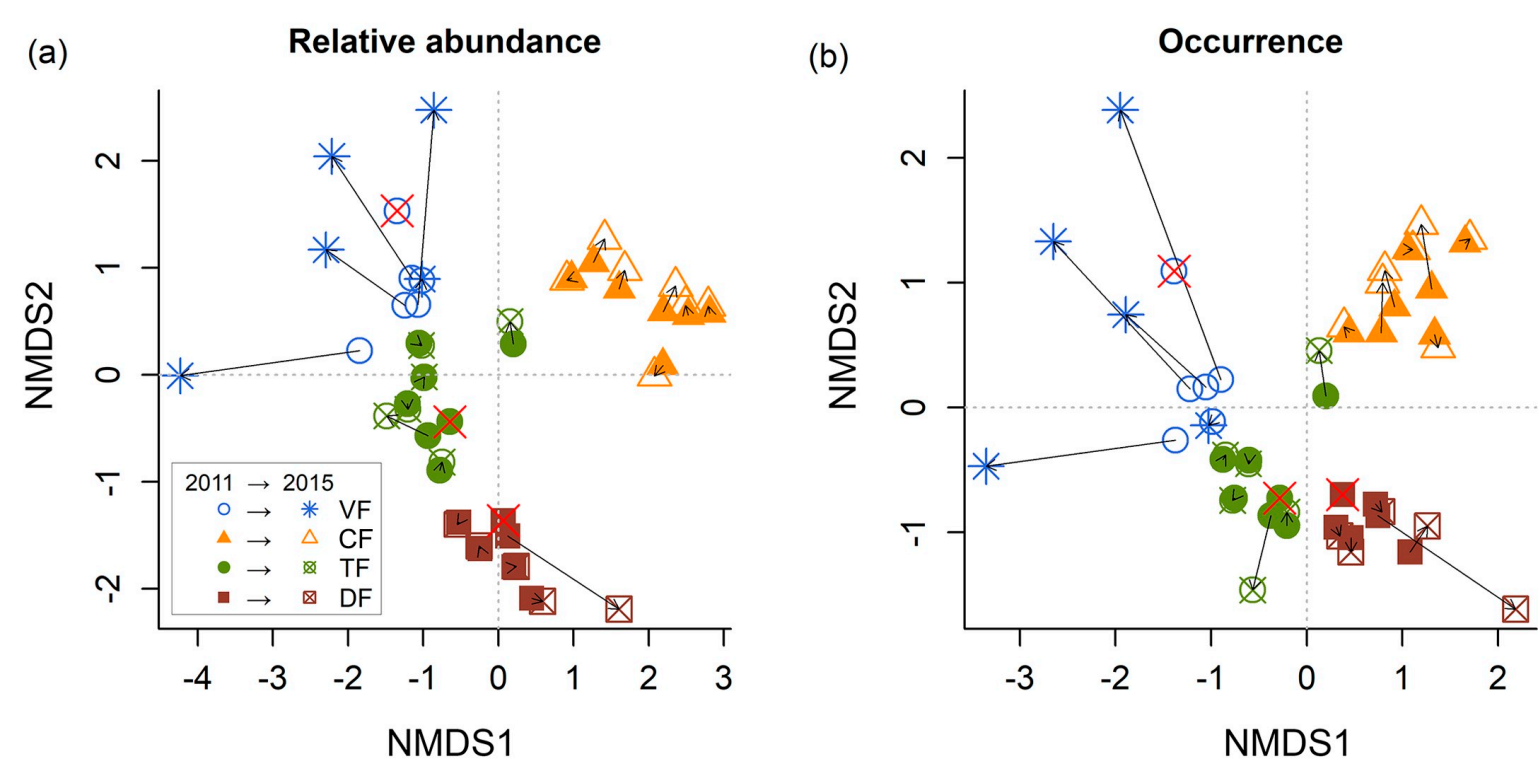
Non-metric dimensional scaling (NMDS) based on relative abundance (a) and species occurrence (b). Arrows indicates the trajectory of 26 plots sampled before (2011) and after (2015) filling the Jirau hydroelectric dam in the Madeira River basin. TF = *terra firme* forests, DF = transitional forests, V = *várzea* forests, CF = *campinarana* forests. Plots with total mortality in 2015 are denoted with a red “×”. For these plots, only 2011 scores are shown.

Plot species composition summarized by NMDS analysis of both presence/absence and abundance data measured in 2011 and 2015 shows significant changes in plots affected by the 2014 extreme flood compared to non-flooded plots (Fig 6). *Várzea* plots showed drastic changes in species composition, which involved the replacement of a rich and equitable community by forest stands dominated by two pioneer species that colonized the area after flooding. Conversely, *campinarana* plots showed negligible changes in species composition compared to other habitat types affected by flooding. However, we recorded the loss of less abundant *campinarana* species, including 25 singletons and 6 doubletons, as well as reductions in abundance of flood-sensitive species, such as *Cybianthus* sp.1 (100% mortality), *Miconia rimachii* (100%), *Meriania urceolata* (93%), *Miconia prasina* (93%), *Iryanthera juruensis* (85%), *Clusia* sp. (78%), *Remijia* sp. (76%), *Ternstroemia dentata* (97%), *Simarouba amara* (90%), *Neea ovalifolia* (75%) and *Trattinnickia glaziovii* (67%). In contrast, the most abundant species in *campinarana* forests, *Ruizterania retusa*, showed only 0.7% of mortality and an increase in dominance from 33.6% to 40.8% after flooding.

**Fig 7:**
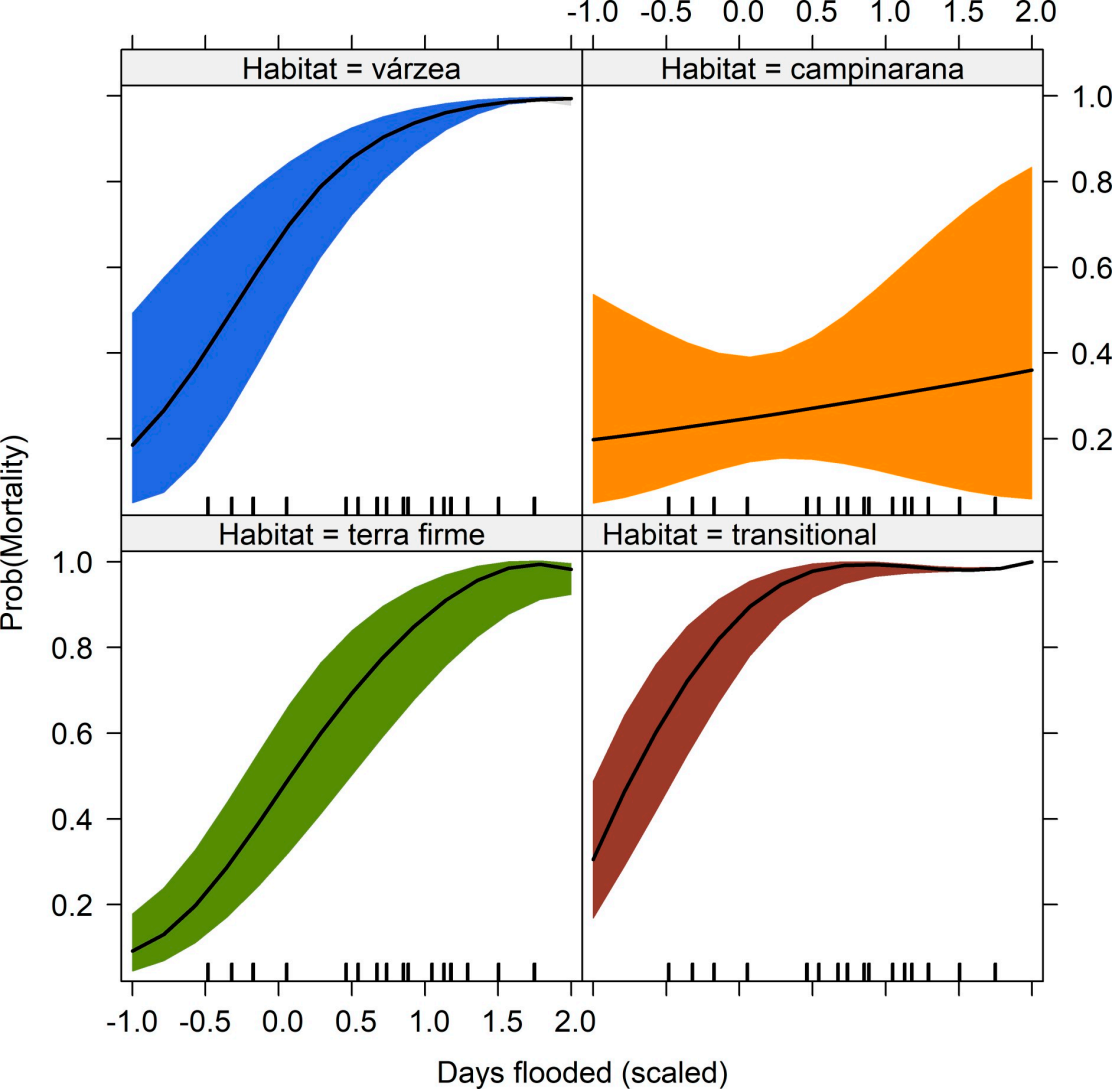
Individual tree mortality probability as a function of Flood Index (FI) in four habitats in the influence area of the Jirau reservoir. Generalized linear mixed effects model (GLMM) was applied to control for random variation in plots. The predictor FI is the number of consecutive days that a plot remained flooded in 2014 (scaled).

Individual mortality rates were strongly associated with the interaction between FI and habitat type (GLMM adjusted for maximum likelihood and Laplace approximation *p*<0.05; Fig 7). All habitat types, except *campinarana* forests, showed a sharp increase in mortality in response to higher FI. Habitat type and FI, as well as the interaction between them, were highly significant predictors of AGB, dead biomass, absolute biomass change over time, and Fisher’s alpha diversity index (Table 3).

**Table 3:** Effects of flooding on biomass and tree diversity in 26 plots before (2011) and after (2015) the Jirau hydroelectric dam in the upper Madeira River.

	Live AGB		Dead biomass		Absolute biomass change over time		Fisher’s alpha	
Effect	L-ratio_(df)_	*P*	F-ratio_(df)_	*P*	F-ratio_(df)_	*P*	L-ratio_(df)_	*P*
FIxHabitat	32.02 _(10;7)_	<0.0001	14.25 _(44;47)_	<0.00001	26.82 _(44;47)_	<0.0001	32.07 _(10;7)_	<0.0001
FI	68.13 _(10;6)_	<0.0001	57.83 _(44;48)_	<0.00001	101.07 _(44;48)_	<0.0001	70.63 _(10;6)_	<0.0001
Habitat	44.23 _(10;4)_	<0.0001	12.67 _(44;50)_	<0.00001	23.20 _(44;45)_	<0.0001	52.95 _(10;4)_	<0.0001

Flood Index (FI) is the number of consecutive days that a plot remained flooded in 2014 and habitat type (*terra firme*, transitional, *várzea* and *campinarana* forests) were predictors with significant interaction in all models. Live aboveground biomass (AGB) was evaluated with a linear mixed effects model, including plot as a random effect. Dead biomass and absolute biomass change over time were evaluated with simple linear regression (R^2^ adjust = 84.05% and 90.31%, respectively, p<0.0001). Response of Fisher’s alpha diversity index was assessed with a mixed effects model, including transect as a random component.

## Discussion

### Large-scale impacts of the 2014 flood on the Madeira River basin

The 2013–2014 period in the Madeira River basin showed exceptional levels of rainfall in several tributaries, mainly Madre de Dios, Beni and Mamoré, according to analysis of CHIRPS data for each sub-basin. Rainfall peak occurred in January 2014, when precipitation volume in the sub-basins of the Andean tributaries exceeded 75–39% of the averages recorded in four decades. Intense rains, mostly in the Andean headwaters caused the highest level and flow of the Madeira River ever recorded at the Porto Velho-RO station [17], causing flooding in the lowland areas of the city [18]. It is believed that the warming of the Indo-Pacific and the subtropical South Atlantic Oceans, as a probable consequence of global climate changes, was the mechanism associated with exceptionally high rainfall between 2013 and 2014 in southwestern Amazonia [16].

The interaction between the new flood pulse, as controlled by the hydroelectric dams, and the new scenario of global climate uncertainties increases the difficulty of forecasting the long-term environmental impacts of mega-dams in the Amazon. Such interaction was evident from the unforeseen flooding of long stretches of BR-364 in the area of the Jirau dam, a vital highway that connects the states of Rondônia and Acre in Brazil with Peru. According to the EIA [49] about 20 km would be affected in the vicinity of Mutum-Paraná (at middle section of the Jirau reservoir), therefore works were carried out to increase the level of the highway. However, these changes were not sufficient to overcome the historic flood of 2014, causing the interruption of BR-364 and isolation of the locations of Guajará-Mirim, Nova Mamoré, Abunã in addition to the whole state of Acre [50]. As we reinforced earlier, the risk of exceptional water flow of the Madeira River in the face of extreme weather events was not properly addressed by the EIA as a requirement for obtaining the hydroelectric license, in addition to the failure to recognize and evaluate other relevant issues [28]. Therefore, we highlight the importance of a new licensing approach that considers the interactions between ongoing climate changes and the construction of mega-dams in the Amazon, and its associated impacts. A critical need to reformulate EIAs for the licensing of hydroelectric dams was also pointed out in a recent study in which the authors observed the multiple effects after the installation of the Balbina hydroelectric dam in central Amazon. The operation of this hydroelectric plant drastically changed the natural flooding pulses of the Uatumã River downstream of the dam [51]. Flooded area by Balbina was predicted by Eletronorte to be 1654 km^2^, while posterior analyzes detected 2996 km^2^ [29, 52].

We found that the impact of high rainfall on river level and flow was amplified in an exceptional way Madeira River mega-dams, affecting areas well beyond the boundaries predicted in the official EIA before project construction [49]. According to this environmental report, a predicted 308 km^2^ of natural vegetation would be lost by the filling of Madeira River mega-dams and whose sum of flooded areas would be 529 km^2^. However, the estimates of Cochrane et al. [18] also using Landsat images, but considering a buffer at least three times bigger (20 km) than the one used here, indicate that the reservoirs of Jirau and Santo Antônio are at least 341 km^2^ (64.5%) larger than predicted in the EIA, with an additional 160 km^2^ of forests loss by the flood beyond expected. Our analyses indicated that the total forest loss in 2014 and 2015 was even greater in these areas where flooding was not predicted, totaling 190.1 km^2^ in Jirau and 56.0 km^2^ in Santo Antônio, representing an excess of 79.9% of forest loss over that expected in the EIA. It is important to emphasize that the estimates of the flooded area presented by both studies are certainly lower than the actual flooding, which in many areas was below the forest canopy, and therefore not detected by the optical sensor used for imaging. In fact, estimates of the 2014 flood peak based on radar sensor available for the Jirau region indicates that the flooded area measured by Landsat images (127.49 km^2^) underestimates real the extent of the 2014 inundation by 435%.

The rate of forest loss was at least five times higher in the region of the Jirau and Santo Antônio reservoirs (9.2 and 7.4%, respectively) compared with the other assessed sub-basins (0.5–1.7%) not affected by the mega-dams. This indicates that the 2014 extreme flood in the Madeira basin was caused primarily by the Jirau and Santo Antônio dams, which amplified the effects of observed extreme rainfall, inundating large areas upstream of reservoirs, which confirms our working hypothesis. Flooding in the Mamoré, Beni, Guarporé and Madre de Dios sub-basins caused a forest loss of 1.4%, 0.7%, 0.5% and 0.7% relative to the respective total buffer areas within each sub-basin. Although proportionally well below that observed in the areas affected by the mega-dams, these values represent a significant amount of forest loss (327 km^2^), which must be related to rainfall extremes in the 2013–2014 period, considering that these sub-basins were not directly affected by the Jirau and Santo Antônio dams.

Although showing a similar proportion of flooded area caused by the 2014 inundation (around 6%), Madeira basin tributaries upstream of mega-dams and areas directly affected by Jirau and Santo Antônio dams differed markedly in the rates of forest loss. We interpret this result as an indication that inundation in Madeira tributaries not affected by dams was more similar to natural flood pulses, reaching forests naturally more resilient to floods. On the other hand, flooding in the area affected by dams was more intense and harmful to vegetation, reaching forests not adapted to long-term flooding, resulting in increased rates of forest loss.

### Impacts of the 2014 flood on monitored forest plots

In addition to basin-wide forest loss detected from satellite data, our permanent forest plots revealed high mortality rates, reduction in AGB and substantial changes in species composition in areas affected by the 2014 flooding. It must be noted that satellite data can underestimate the effect of flooding on tree mortality, community structure and species composition. For example, in some cases, areas affected by flooding at different levels were recognized by satellite data as living forests. Such areas, although still standing after flooding, experienced species loss, as well as changes in functional composition [17, 20], which cannot be detected by remote sensing data alone. This is more challenging with medium resolution optical images such those from Landsat.

The loss of *várzea* forests in areas upstream of the Jirau dam was markedly greater than in other vegetation types owing to the landscape configuration of these habitats in association with rivers. Contrary to our expectations, *várzea* forests, a vegetation type that naturally experiences seasonal flooding, was severely affected by inundations. This can be explained by some peculiarities of *várzea* forests in the upper Madeira River. In that region, the Madeira River has a deep channel and high riverbanks, and *várzea* forests occupy only a narrow (10–20 m) zone along the banks of the river, which experiences only limited flooding during high-water periods [38] compared to the extensive seasonal inundation in central Amazonia floodplains. Consequently, *várzea* forests in the upper Madeira River are less tolerant to high-intensity flooding, which could explain the elevated tree mortality observed. It is noteworthy that even the low-várzea, a habitat type much more adapted to inundation, could have a significant tree mortality if the flood pulse loses its regularity [14].

It has been shown that *várzea* forests in the upper Madeira basin harbor a species composition distinct from that of adjacent *terra firme* forests [17, 38] (Fig 4) and that community assembly is driven by distinct environmental filters and dispersal limitations [21]. The spatial configuration of *várzea* forests and their sensitivity to intense flooding make this habitat more vulnerable to inundations. In this case, the species-area effect is itself an important conservation issue because habitat loss is directly associated with species loss, mainly affecting rare species [53, 54]. It has been shown that rare species are more seriously threatened by artificial flooding and deforestation [55], as well as habitat fragmentation associated with mega-dams in the Amazon [56]. Studies carried out in the area of the Jirau dam reported that populations of trees and palm species associated with *várzea* forests were the most seriously affected by the filling of the reservoir [19, 20]. We found that 41 out of 326 morphospecies surveyed in five *várzea* plots were lost after the 2014 flood, indicating that populations of a considerable fraction of the regional species pool, particularly flooding-sensitive species that occur in low abundances, were severely affected.

Although highly threatened by extreme climatic anomalies and constructions of dams, *várzea* forests play a key role in floodplain ecosystems due to high biomass production, low decomposition rates and availability of non-timber forest products for riverine people [57, 58]. These riparian forests also provide various ecological services, such as erosion and flood control, mitigation of nutrient leaching from agricultural areas, maintenance of water quality and support for biodiversity including food for terrestrial and aquatic fauna [59].

Although having less impact, the 2014 extreme flood also affected forest formations distant from the Madeira River banks, such *terra firme*, transitional and *campinarana* forests. Despite the limited reduction in AGB, *campinarana* experienced an increase in tree mortality, with elimination of flood-sensitive species in extensive stretches of this formation. Such selective tree mortality in *campinarana* forests resulted in functional changes at the community level towards a suite of more conservative life strategies, as shown in a recent study [17]. It has been shown that species in *campinarana* forests undergo a strong environmental filter, largely mediated by the natural rise of the water table in the rainy season, which exerts selective pressure in this habitat, favoring dominance by flood-tolerant species [21]. This probably explains the milder impacts experienced by *campinarana* forests compared to other habitats, despite high flooding intensity experienced by all *campinarana* plots during the 2014 flood, which is in line with our expectations. On the other hand, both *terra firme* (three plots) and transitional forests (two plots) affected by flooding showed dramatic reduction in abundance, biomass and species richness. This outcome was expected since these forests mostly grow on well-drained soils where species highly sensitive to flooding predominate.

### Basin-wide impacts of 2014 flood for carbon stocks

In the last decades of the 20th century, forests underwent accelerated growth and increased biomass, which practically balanced the carbon emissions in the atmosphere from Amazon deforestation [60]. However, more recent research has shown that tree mortality appears to be increasing in tropical rainforests, with important implications for global carbon cycle [61]. Changes in the frequency or severity of climate extremes could considerably reduce carbon absorption and contribute to climate warming [62]. Extreme climatic events associated with droughts, heat, storms, and high precipitation have increased tree mortality, especially in the functional groups of pioneers, softwoods and evergreens, accelerating nutrients and carbon cycling, but reducing the forest’s ability to fix carbon and nutrients in living biomass [63].

The 2014 flood in the upper Madeira basin dramatically affected forest carbon stocks, with substantial AGB losses in most habitats surveyed. Taking together the estimated area of forest loss in the Madeira River basin and tributaries (747 km^2^; Table 1), along with our field measurements of biomass in forest plots (AGB range of 118 to 167 tons per hectare), we estimate that the 2014 flood would have resulted in 8.81–12.47 ∙ 10^6^ tons of dead biomass with significant implications for greenhouse emissions. These numbers are probably underestimated since tree mortality could happen several years after flooding. Also, we only considered in these calculations forest loss as estimated by satellite data; thus, tree mortality that occurred in sites with less affected forests was not taken into account here. The recovery of biomass lost during the 2014 flood is uncertain, as forest regeneration will depend on a number of factors, including the frequency and intensity of future floods.

The 2014 flood damage observed in areas without the influence of the dams indicates the natural susceptibility of riparian forests to episodic flooding events. This finding is relevant considering the evidences of an intensification of the Amazon hydrological cycle in the last decades, with a general trend toward an increment in the annual amplitude of river discharge caused by abnormal precipitation [64]. As exemplified by our study, these alterations in the hydrological cycle have the potential to cause large carbon emissions associated with losses of Amazonian riparian forests.

## Conclusions

Our study demonstrated that mega-dams were mainly responsible for the high forest mortality in the upper Madeira River basin after the 2014 extreme flood, with forest loss rates at least five times higher when compared to the losses resulting from rainfall extremes. In addition, the results also showed, in an unprecedented way, that the extreme rainfall anomalies, probably triggered by global warming and intensified in the last decades, also represent a key driver of forest mortality in the Amazon, even in areas not affected by dams, as observed for the Beni, Guaporé, Madre de Dios and Mamoré River basins. Finally, the interaction between extreme rainfall events, and the installation of mega-dams has negative consequences of extensive magnitude for species composition, functionality and biomass stocks of Amazonian forests, with various habitats responding differently to flooding intensity.

Considering the magnitude of 2013/2014 extreme weather event and its consequences, which were not foreseen by the environmental impact studies, a new approach for the environmental licensing of hydroelectric dams in the Amazon region becomes a matter of urgency. Alternative plans to mitigate the synergic effects of damming rivers and extreme climate events should be considered. The proliferation of dams and the increase of uncertainties about climatic anomalies place at risk different ecosystems in the Amazon basin, threatening their rich biodiversity with more drastic effects on floodplain forests.

## Supporting information

S1 Appendix The following figures contain the classification of land cover of 30 sections of the Madeira River Sub-basins and the effects of the 2014 extreme flood are indicated. Scale in UTM coordinates. Diagrams show the 6-km-wide buffer (Buffer) on each bank along the course of the river, percentage of permanent surface water (Permanent water), flooded area at the peak of 2014 extreme flood (Flooded) visible by optical sensors (Landsat), flooded area peak along Jirau reservoir estimated by radar sensor is denoted by the black line (data provided by ESBR), forest loss two years after flooding (2014+2015), highlighting forest loss from filling the reservoirs (yellow) and the loss of forest of unforeseen areas beyond the predicted limits of the reservoirs (red). Deforestation by logging, which was not considered in the calculations of forest loss caused by flood, is shown in purple. Forest loss metrics are presented with the respective percentage of forest loss relative to the area of standing forest in 2013. Locations of dams are indicated by grey bars. Additional analyzed buffers are presented in S1 Appendix. Permanent superficial water and flooded area data from EC JRC/Google. Forest loss data from Hansen/UMD/Google/USGS/NASA.
(DOCX)

S1 Fig Forest monitoring plots along the Jirau reservoir, Rondônia, Brazil. Circles indicate 26 1-ha plots in different habitats: VF = *várzea* forests, CF = *campinarana* forests, TF = *terra firme* forests, DF = transitional forests. Cota 90 represents the initial forecast of flooding by the reservoir, and Flood (2014) represents the peak of extreme flooding as detected by radar/laser sensors (both provided by ESBR). Triangles represent the location of four limnimetric stations along the Jirau reservoir where the level of the Madeira river was measured (see Fig 3). False color composition RGB-654 of Landsat-8/OLI imagery one month after extreme flooding (May/2014), courtesy of the U.S. Geological Survey.
(DOCX)

S2 Fig Estimates of Above Ground Biomass (AGB) per hectare in flooded and non-flooded areas before (pre) and after (post) Jirau reservoir filling. Estimates include data before (pre-dam) and after (post-dam) the Jirau reservoir filling, in four habitats: *terra firme* forests (non flooded N = 4; flooded N = 3), transitional forests (non flooded N = 4; flooded N = 2), *várzea* forests (non flooded N = 1; flooded N = 5), *campinarana* forests (flooded N = 7), considering different vegetation strata. (a) understory: 1 ≤ DBH < 10 cm; (b) canopy: 10 ≤ DBH <30 cm; (c) emergent DBH ≥ 30 cm.
(DOCX)

S1 Table Total rainfall (mm) during the extreme flood period (July-2013 to June-2014) in the Madeira River sub-basins. Total values were calculated from monthly average estimates from the CHIRPS climatic data set at high spatial resolution (0.05° x 0.05°). Area (km^2^) of each basin is indicated, with the respective number of pixels used to represent the climatic data.
(DOCX)

S2 Table Landscape metrics in 30 sections in the Madeira River sub-basins. The landscape sections were established along the river channel from 6 km wide buffers on each bank and area (km^2^) is indicated; permanent surface water area (Perennial); 2013–2014 flooded area (Flooded); 2014 and 2015 forest losses (Forest Loss). Calculation of forest loss in the reservoirs of Jirau and Santo Antônio excluded areas submerged by the filling of reservoirs. Therefore, only areas beyond the hydroelectric reservoirs were considered as forest loss. The percentage deforestation in relation to forest cover before flooding in 2013 (Forest cover) is indicated in parentheses.
(DOCX)

S3 Table Number of individuals of tree species in three diameter size classes in four forest habitats sampled in 26 1-ha plots in the area of influence of the Jirau dam, before (pre—2011) and after (post—2015) the filling of the reservoir. (DOCX)

S4 Table Descriptive statistics (mean ± standard deviation) of diversity metrics and structural variables in 17 plots affected by flooding in four habitats before (2011) and after (2015) the filling of the Jirau. DBH(max) = average maximum individual diameter per plot (cm); DBH = average diameter at breast height (cm); H(max) = average maximum height per plot (m); H = average height (m); AB(max) = average maximum individual basal area per plot (m^2^); AB = average individual basal area (m^2^); WD = average wood density (g cm^−3^); AGB(max) = average maximum individual aboveground biomass per plot (Mg); AGB = average individual aboveground biomass per plot (Mg); Abundance (min, max) = minimum and maximum individuals per plot; Abundance_plot = average individuals per plot; Fisher’s alpha = diversity index; Singletons (min, max) = minimum and maximum species per plot with only one individual per species; Singletons = average species with only one occurrence record per plot; Doubletons (min, max) = minimum and maximum species with only two individuals per plot; Doubletons = average species with only two individuals per plot. Species = average species per plot; Genus = average genera per plot; Family = average families per plot; Total number (#) of species, genera and families in each habitat; (*) values represent minimum and maximum. The number of plots in each habitat is indicated by (n).
(DOCX)

S5 Table Descriptive statistics (mean ± standard deviation) of diversity metrics and structural variables in 9 plots not impacted by 2014 flooding measured before (2011) and after (2015) the filling of the Jirau reservoir. DBH(max) = average maximum individual diameter per plot (cm); DBH = average diameter at breast height (cm); H(max) = average maximum height per plot (m); H = average height (m); AB(max) = average maximum individual basal area per plot (m^2^); AB = average individual basal area (m^2^); WD = average wood density (g cm^−3^); AGB(max) = average maximum individual aboveground biomass per plot (Mg); AGB = average individual aboveground biomass per plot (Mg); Abundance (min, max) = minimum and maximum individuals per plot; Abundance_plot = average individuals per plot; Fisher’s alpha = diversity index; Singletons (min, max) = minimum and maximum species per plot with only one individual per species; Singletons = average species with only one occurrence record per plot; Doubletons (min, max) = minimum and maximum species with only two individuals per plot; Doubletons = average species with only two individuals per plot. Species = average species per plot; Genus = average genera per plot; Family = average families per plot; Total number (#) of species, genera and families in each habitat; (*) values represent minimum and maximum. The number of plots in each habitat is indicated by (n).
(DOCX)
