# Supplementary material for: Mega-dams and extreme rainfall: Disentangling the drivers of extensive impacts of a large flooding event on Amazon Forests
Source: PLoS One. 2021 Feb 12;16(2):e0245991. doi: 10.1371/journal.pone.0245991 (PMC7880702; doi:10.1371/journal.pone.0245991)

**S1 Appendix. The following figures contain the classification of land cover of 30 sections of the Madeira River Sub-basins and the effects of the 2014 extreme flood are indicated.** Scale in UTM coordinates. Diagrams show the 6-km-wide buffer (Buffer) on each bank along the course of the river, percentage of permanent surface water (Permanent water), flooded area at the peak of 2014 extreme flood (Flooded) visible by optical sensors (Landsat), flooded area peak along Jirau reservoir estimated by radar sensor is denoted by the black line (data provided by ESBR), forest loss two years after flooding (2014+2015), highlighting forest loss from filling the reservoirs (yellow) and the loss of forest of unforeseen areas beyond the predicted limits of the reservoirs (red). Deforestation by logging, which was not considered in the calculations of forest loss caused by flood, is shown in purple. Forest loss metrics are presented with the respective percentage of forest loss relative to the area of standing forest in 2013. Locations of dams are indicated by grey bars. Additional analyzed buffers are presented in S1 Appendix. Permanent superficial water and flooded area data from EC JRC/Google. Forest loss data from Hansen/UMD/Google/USGS/NASA.


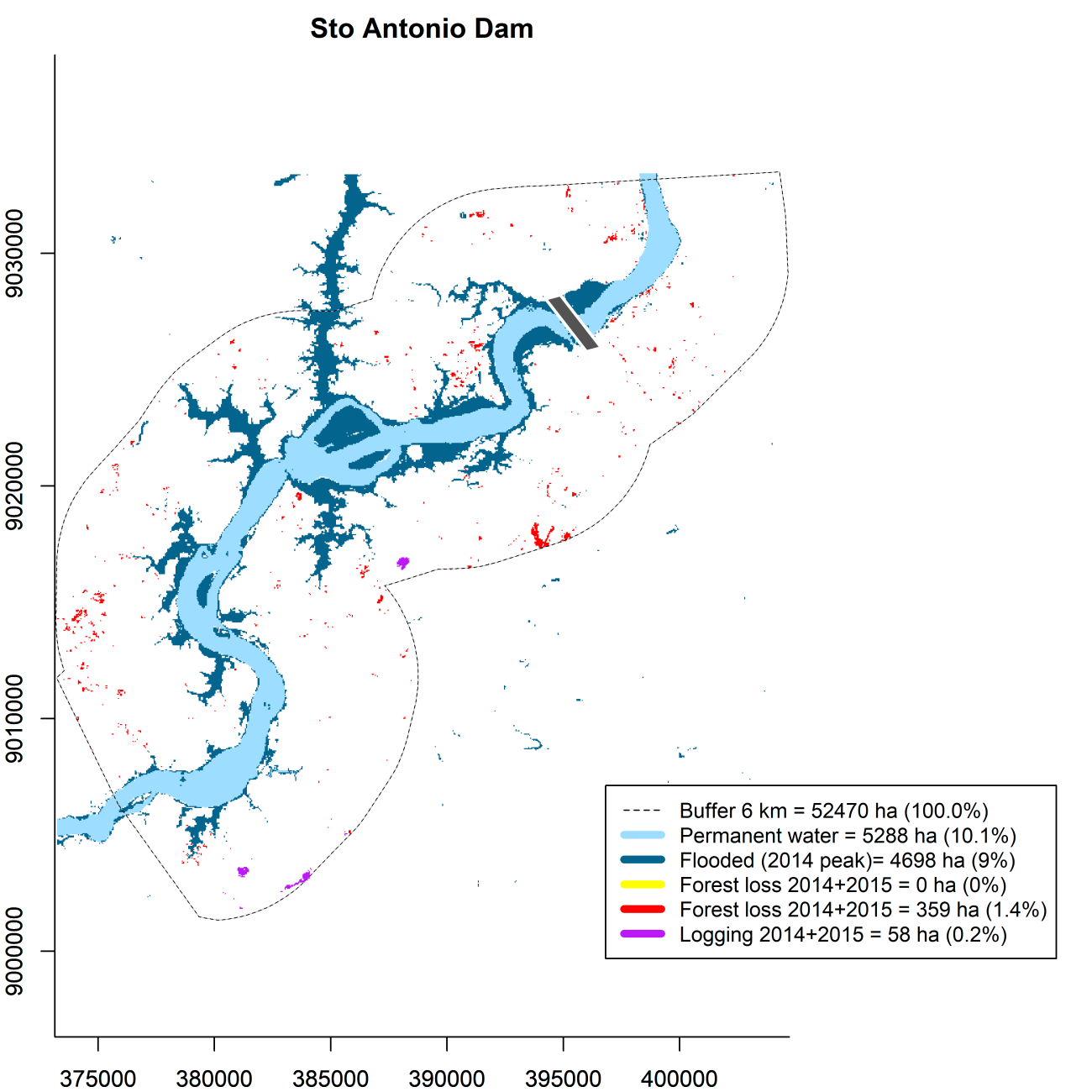


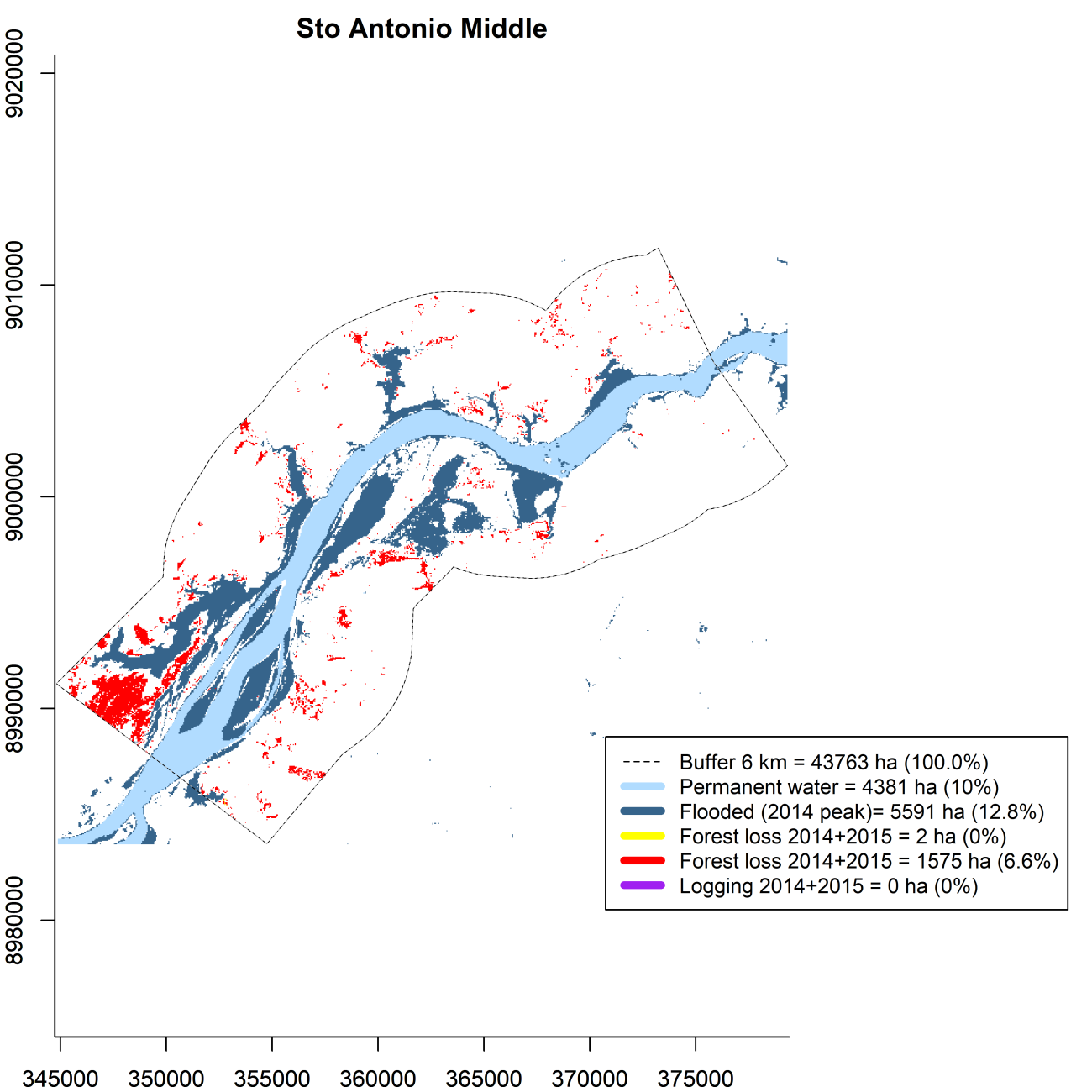


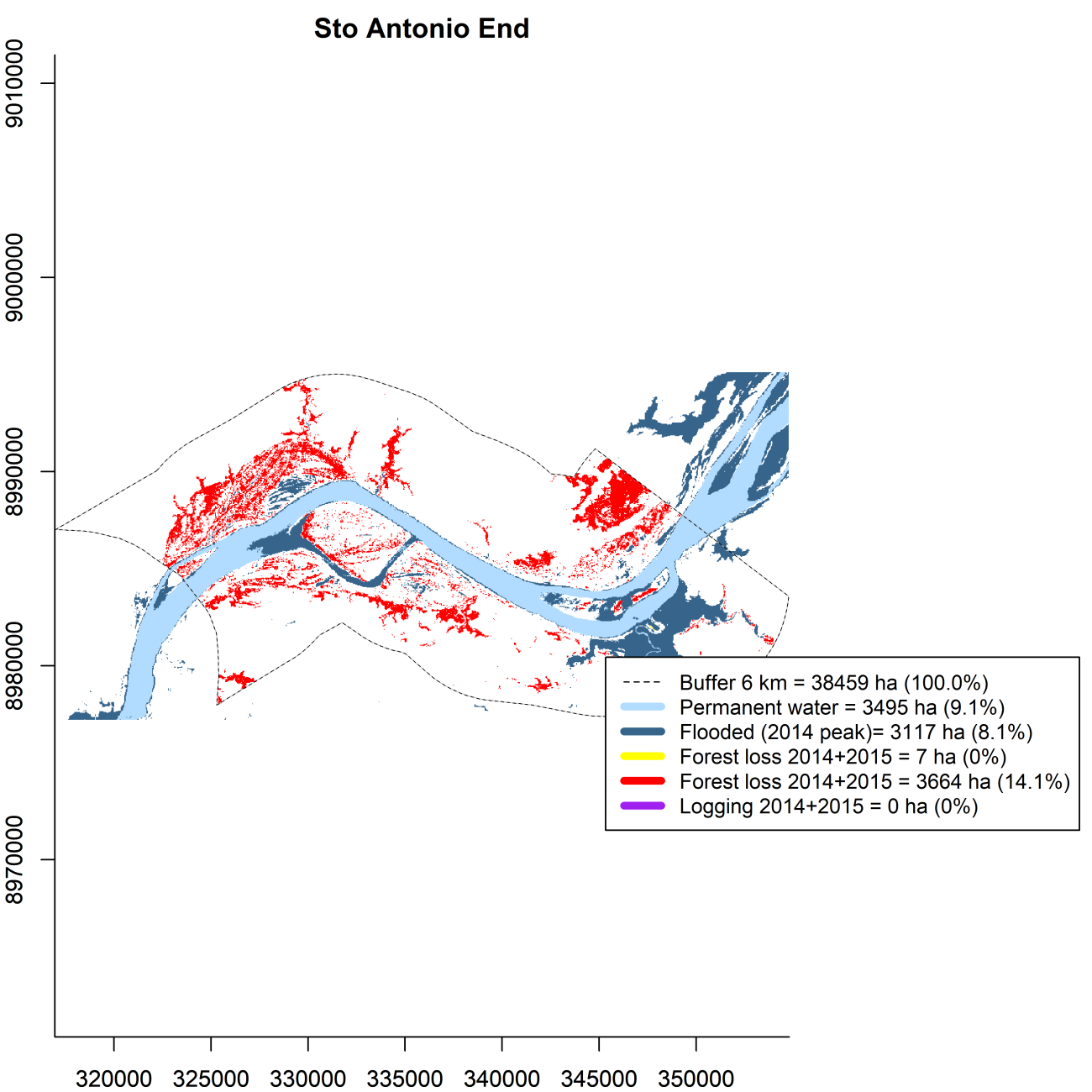


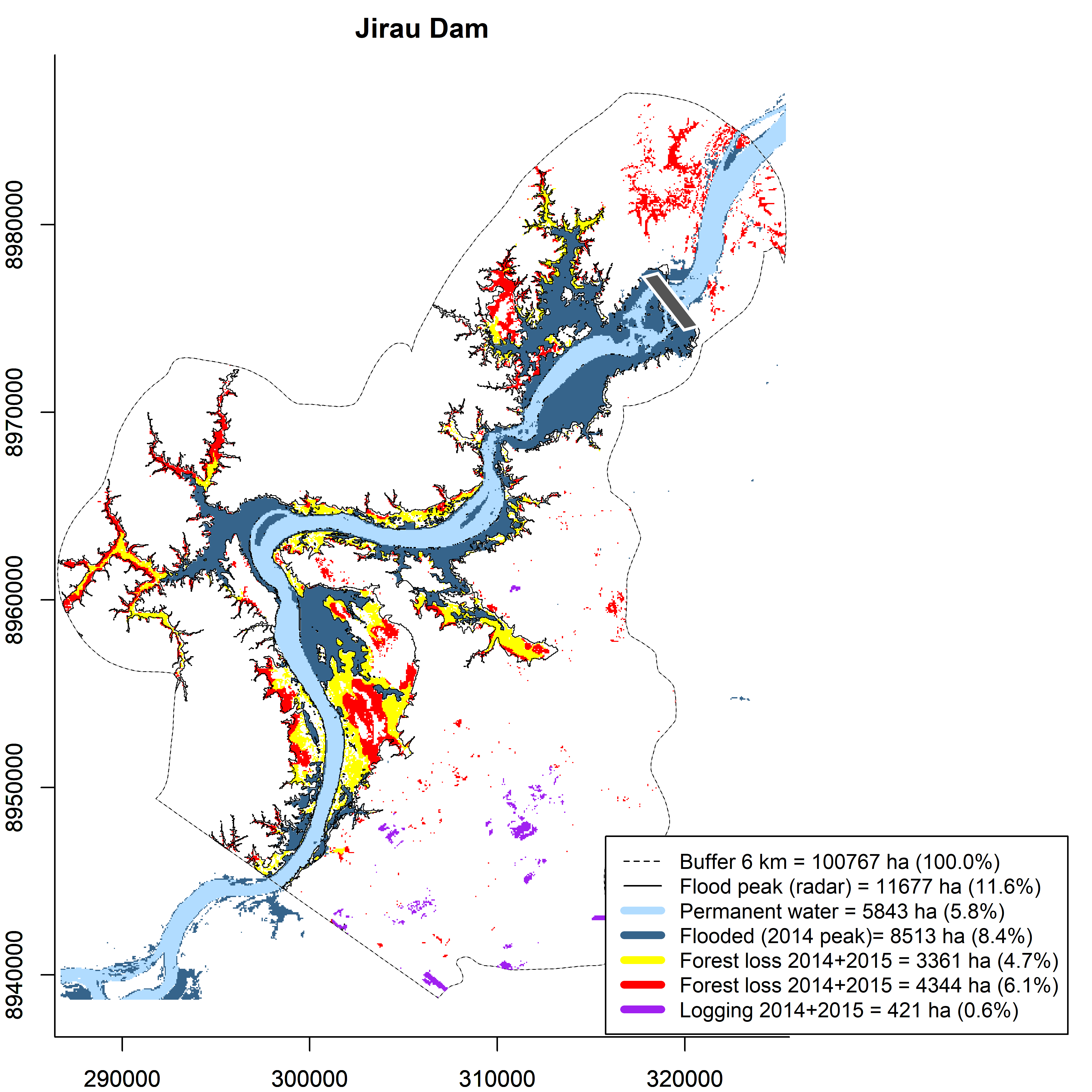

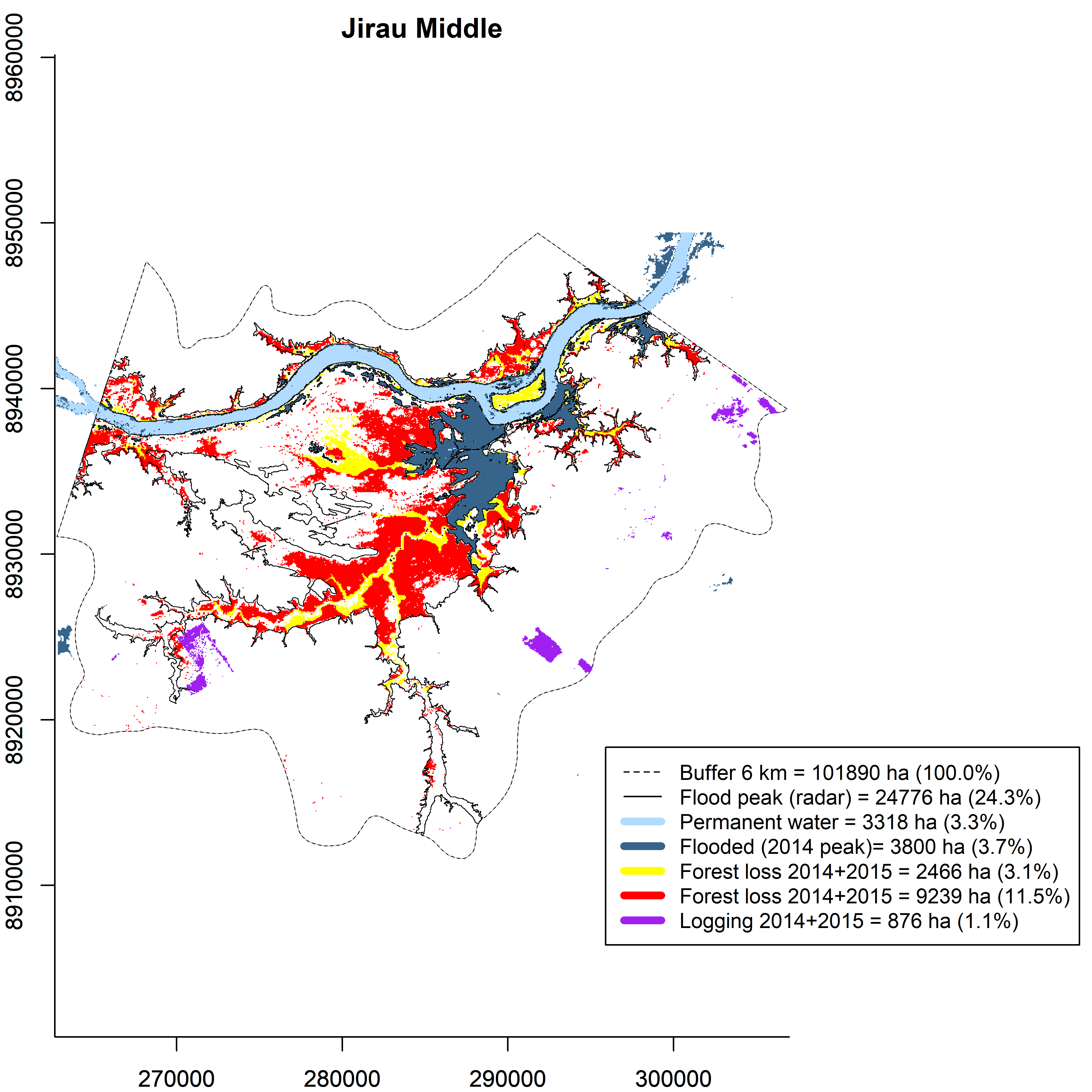

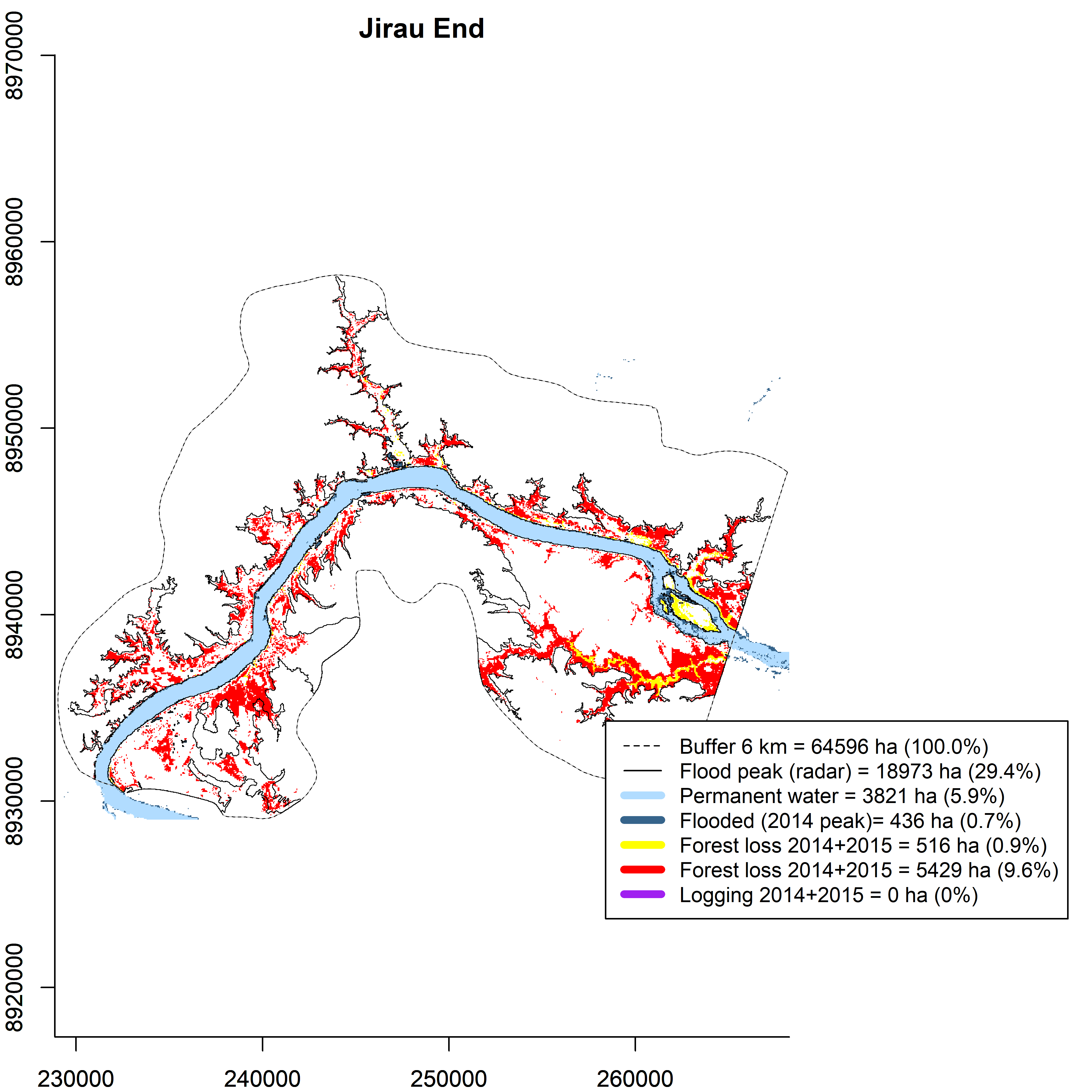

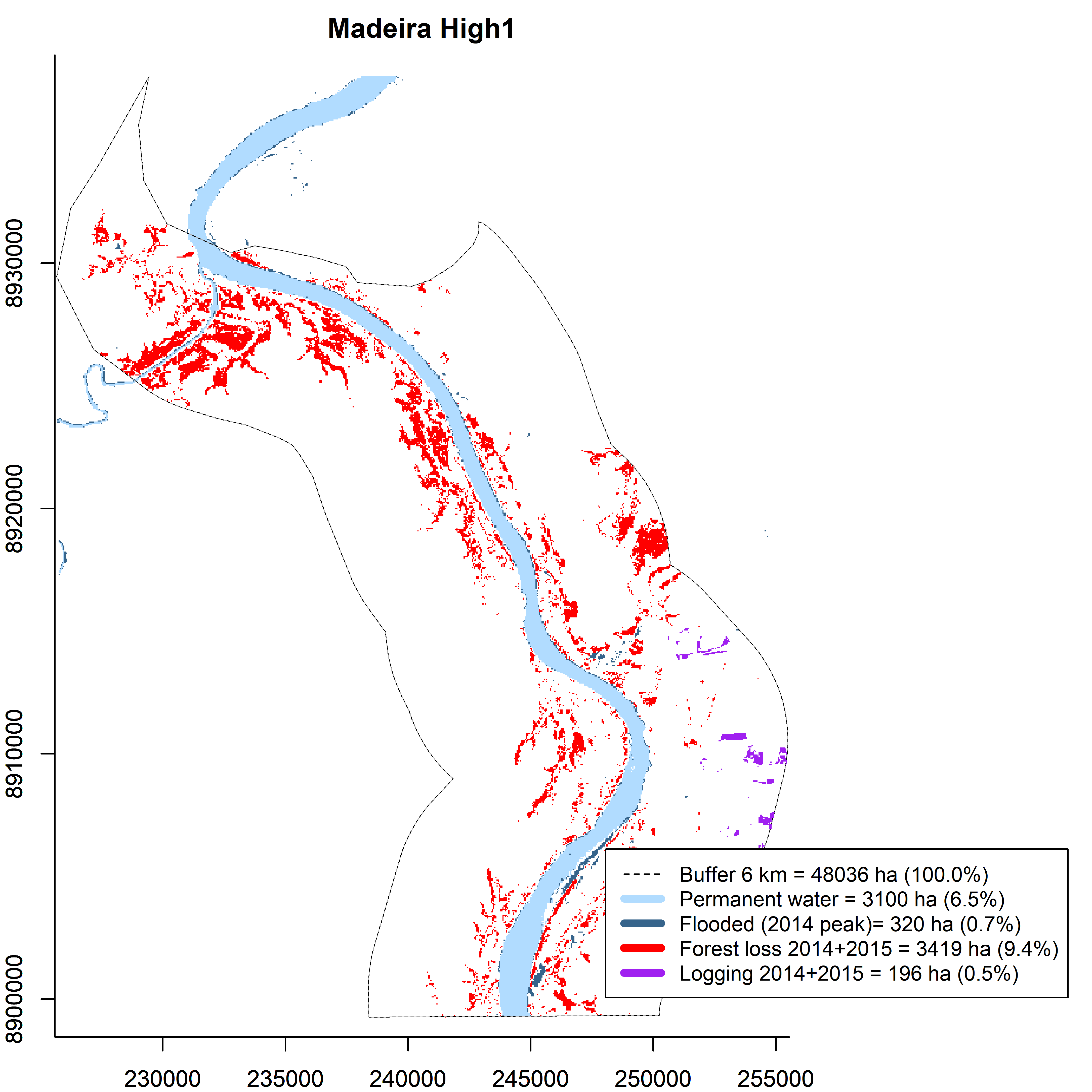

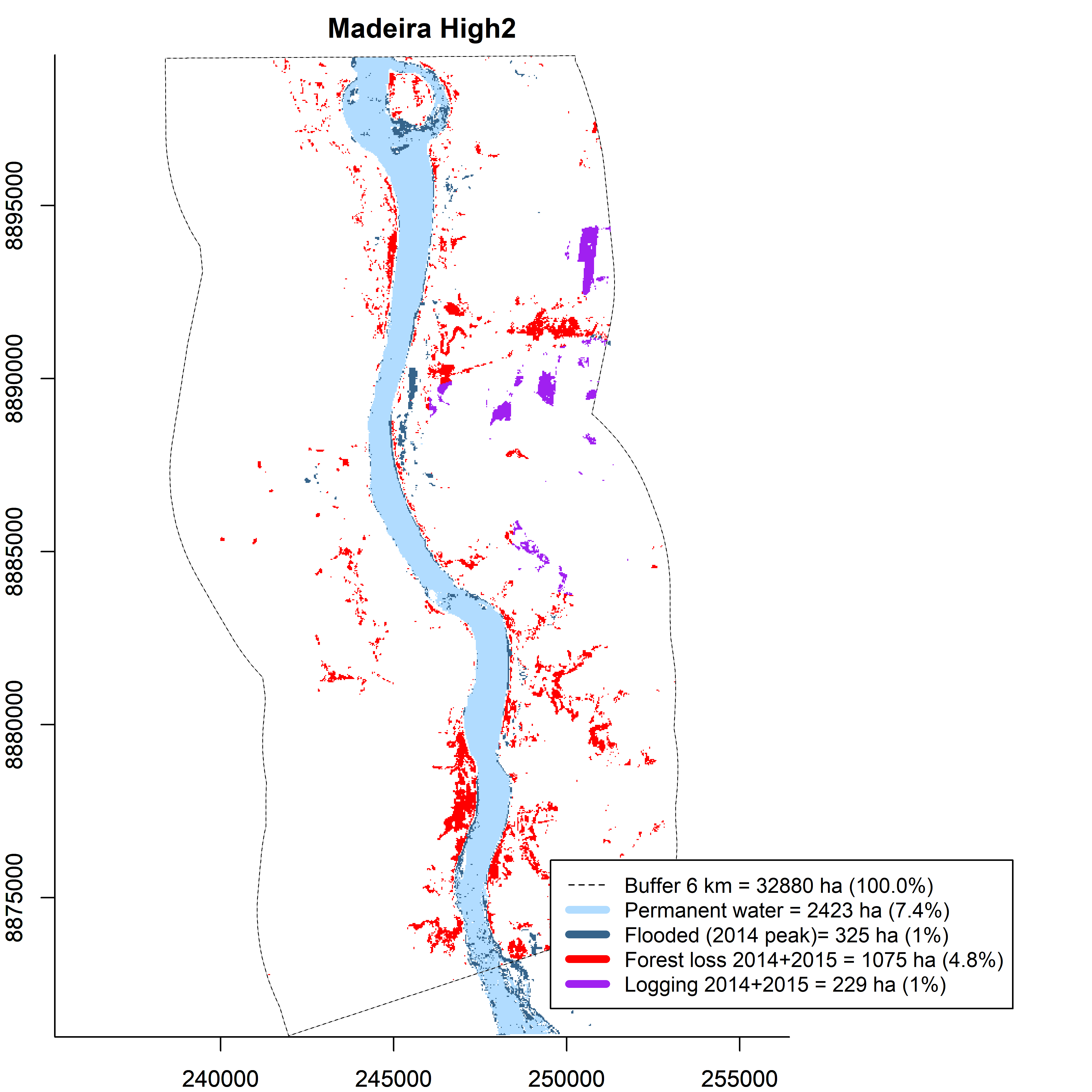

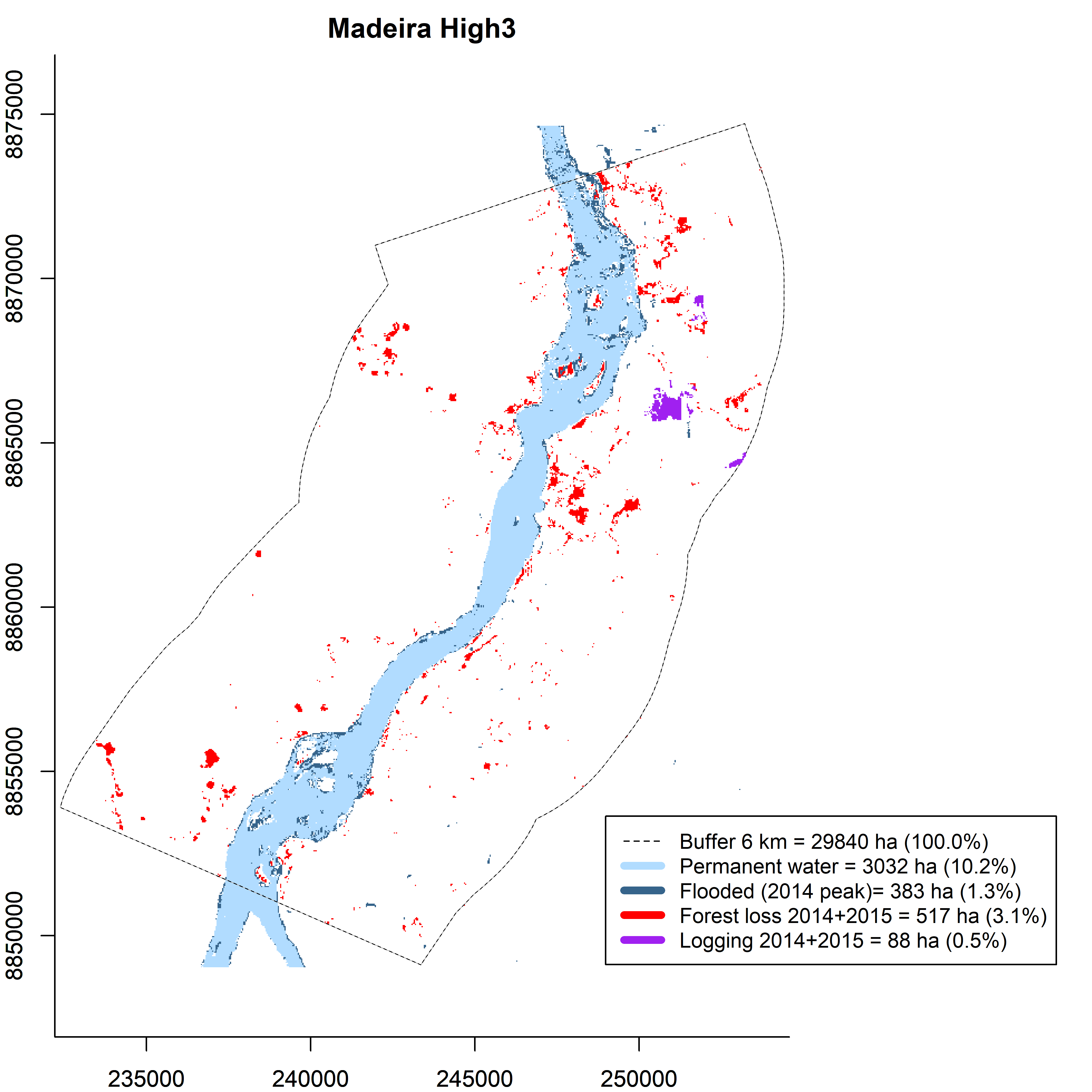

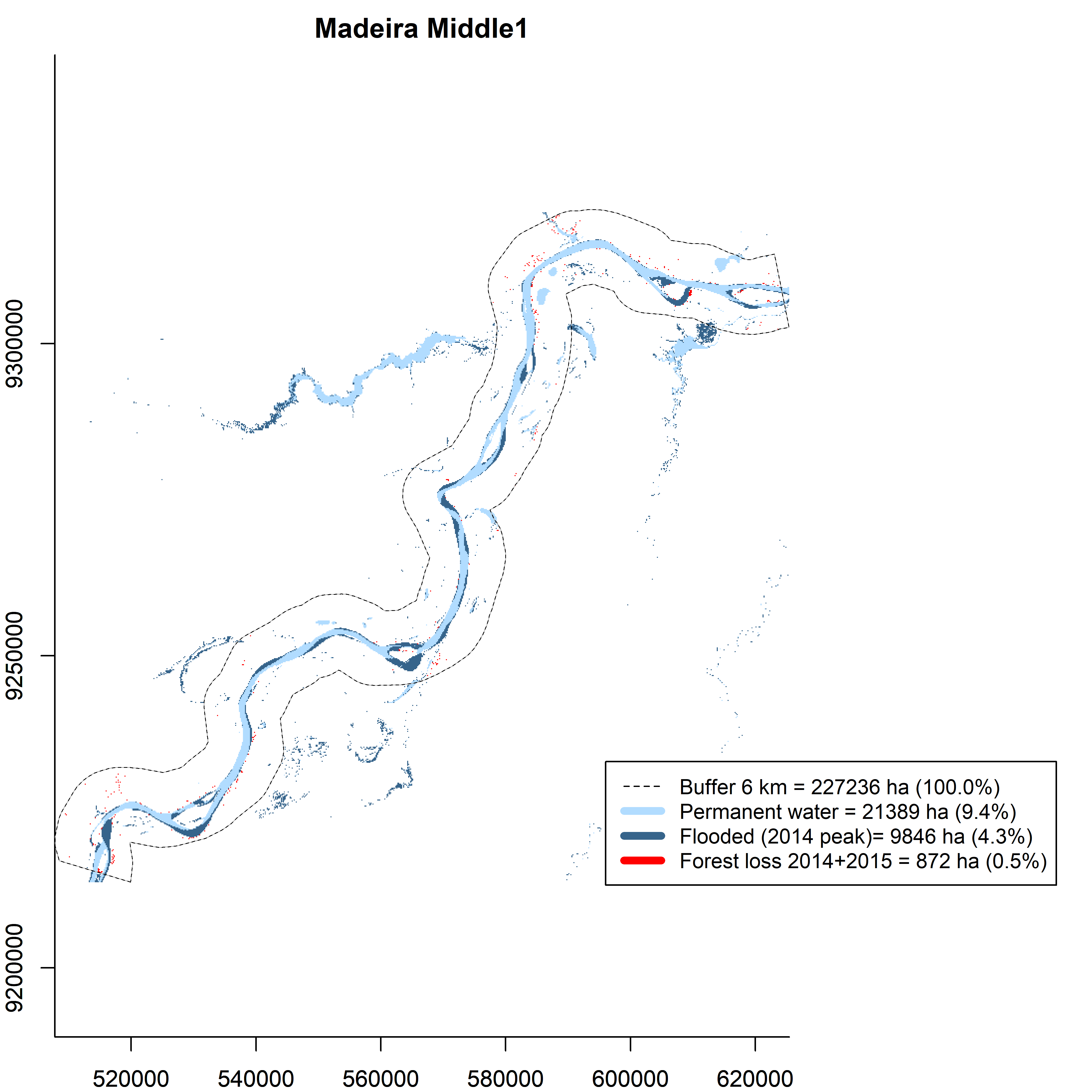

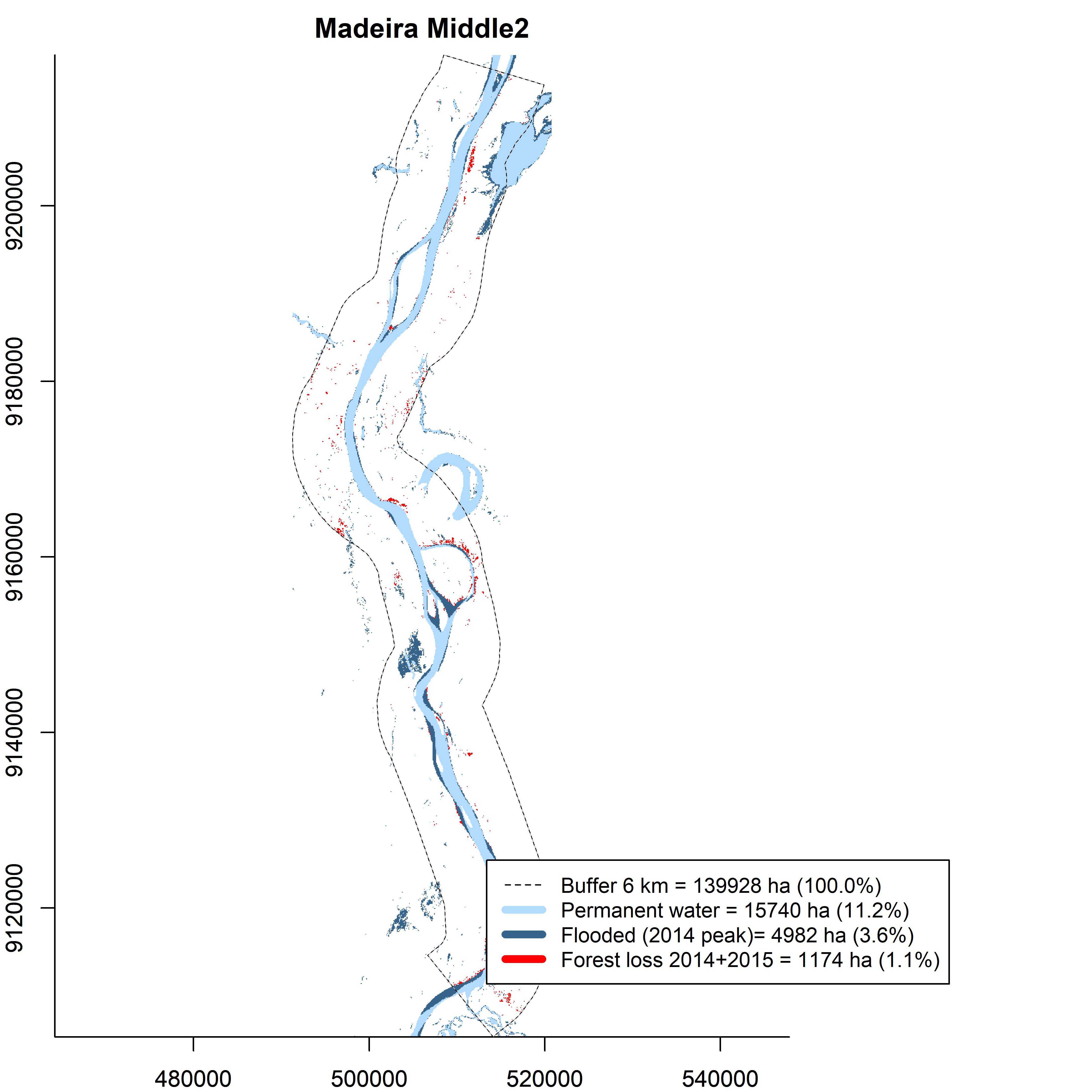

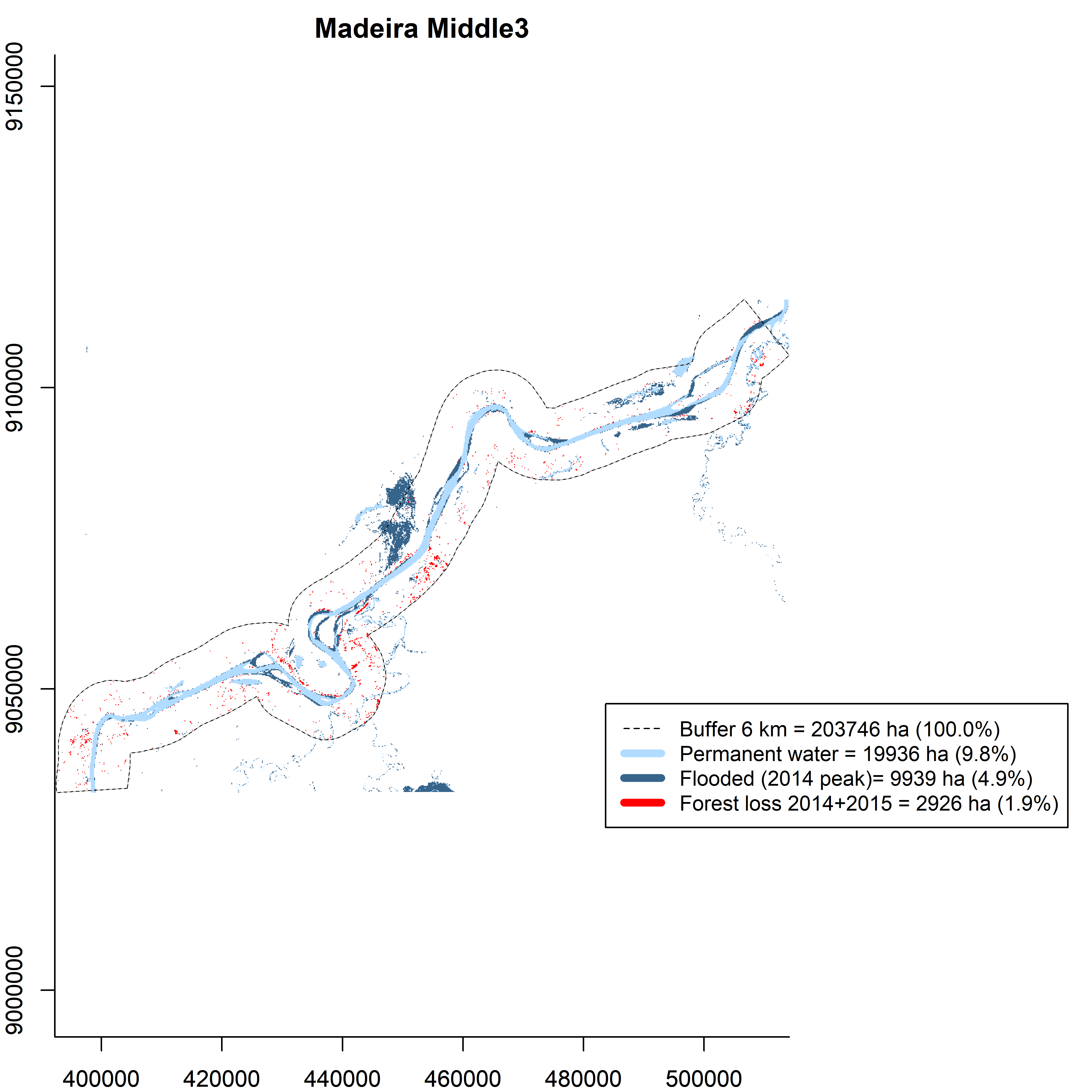


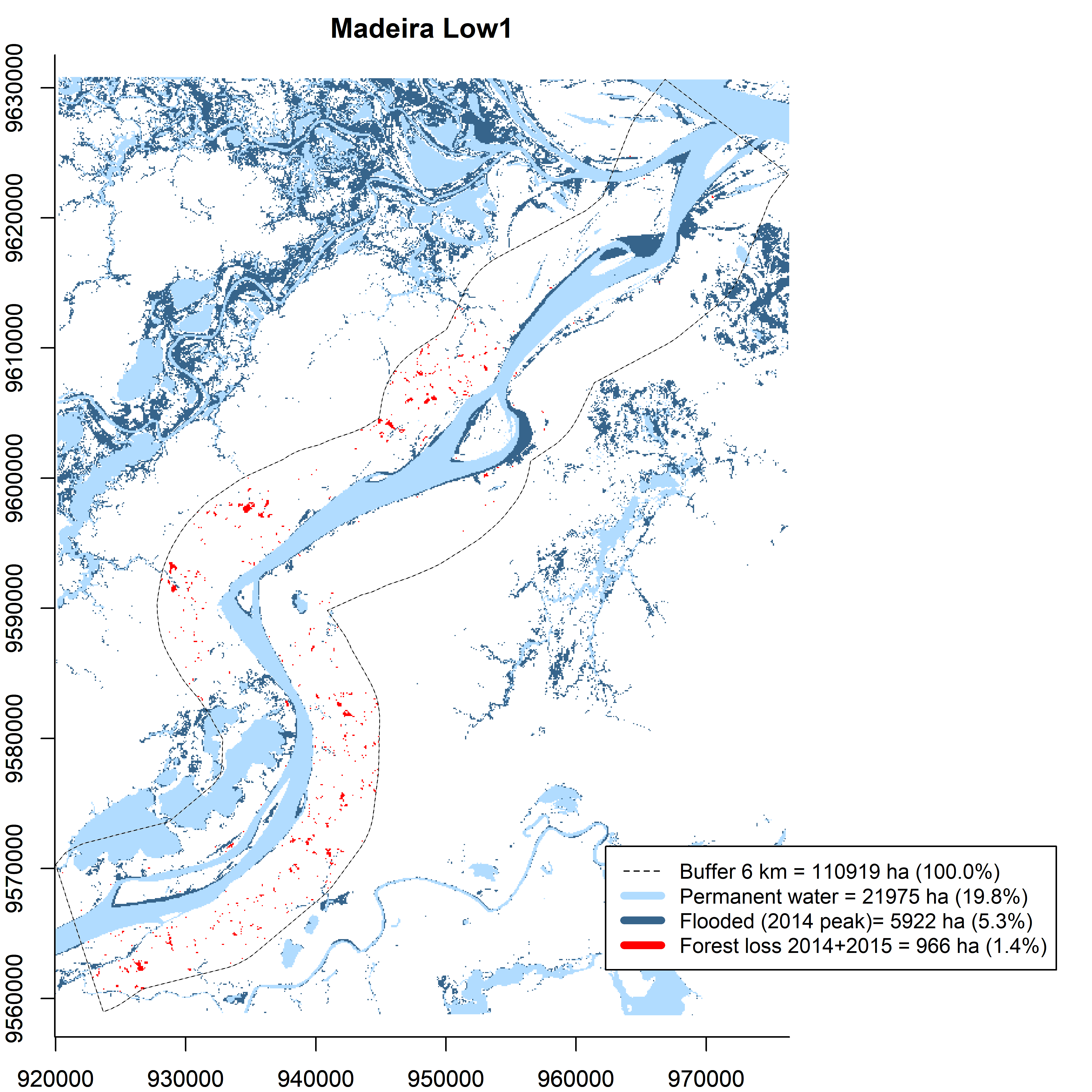

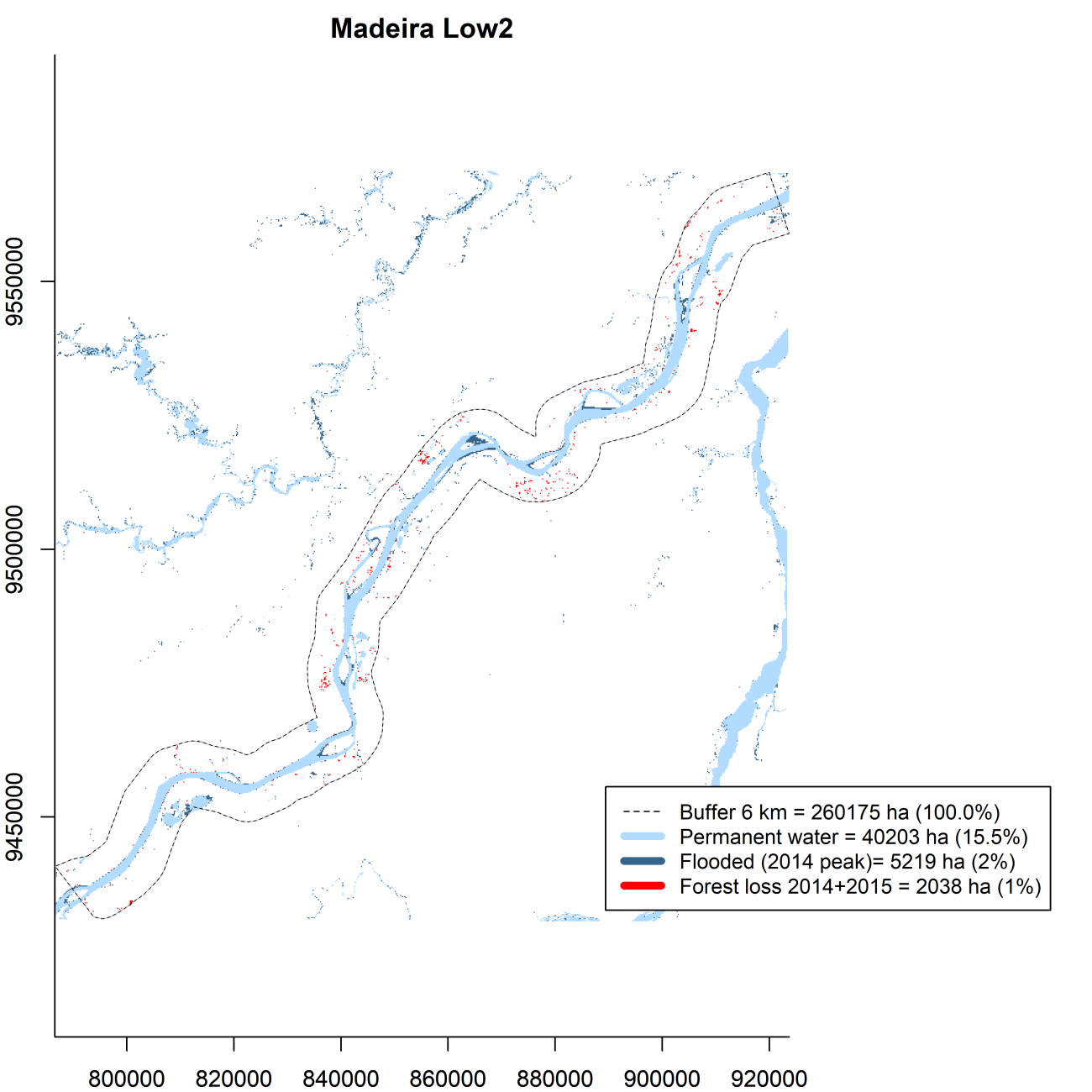

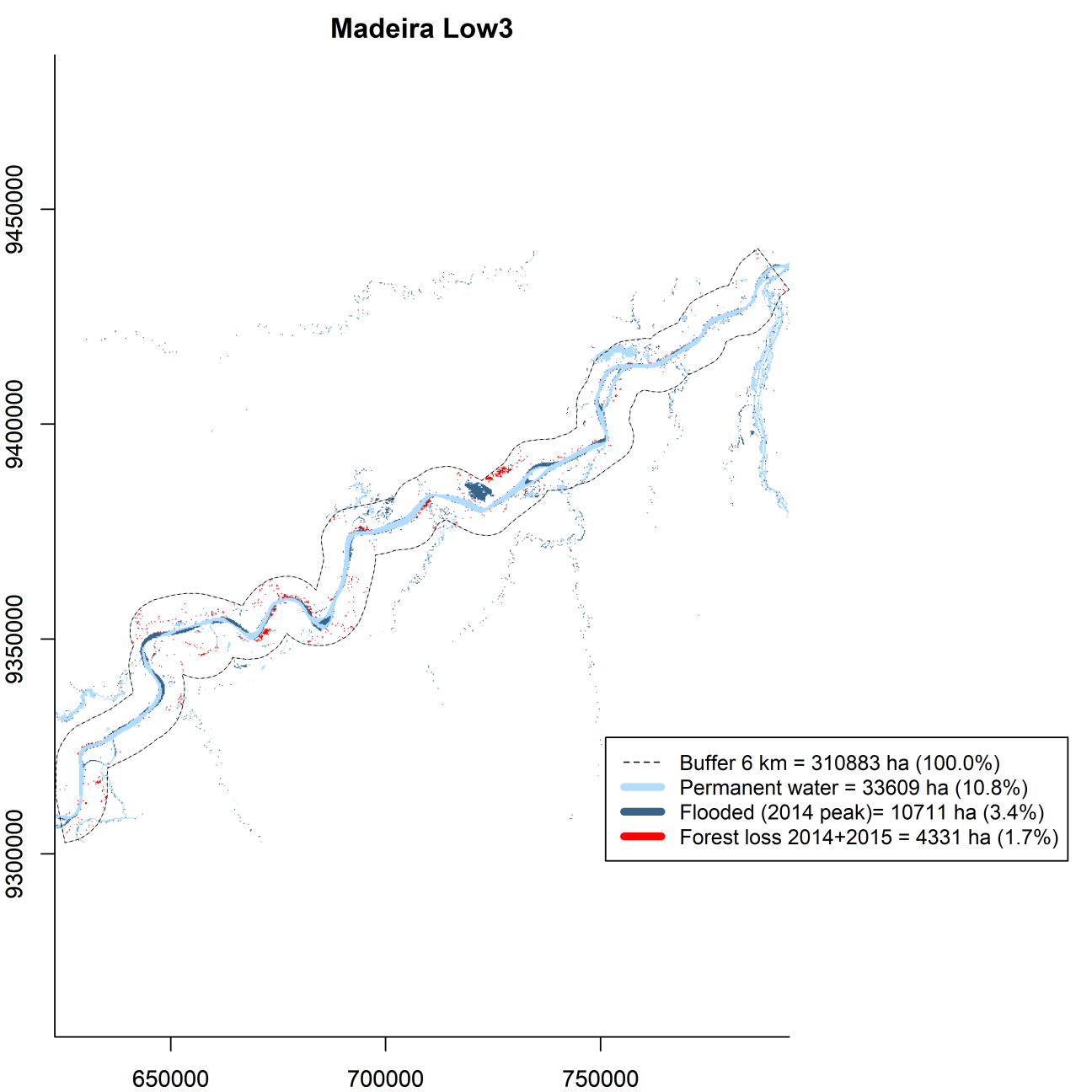


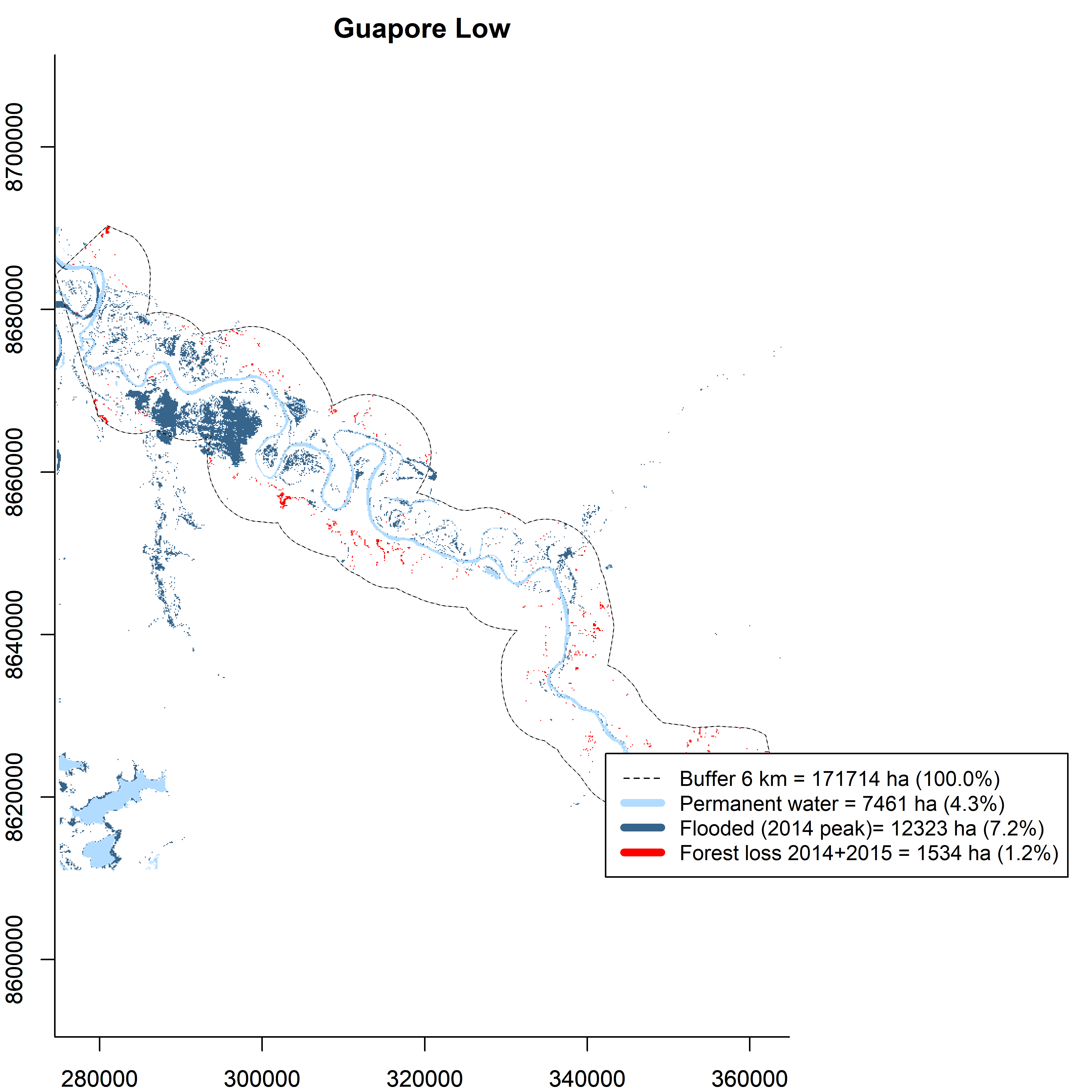

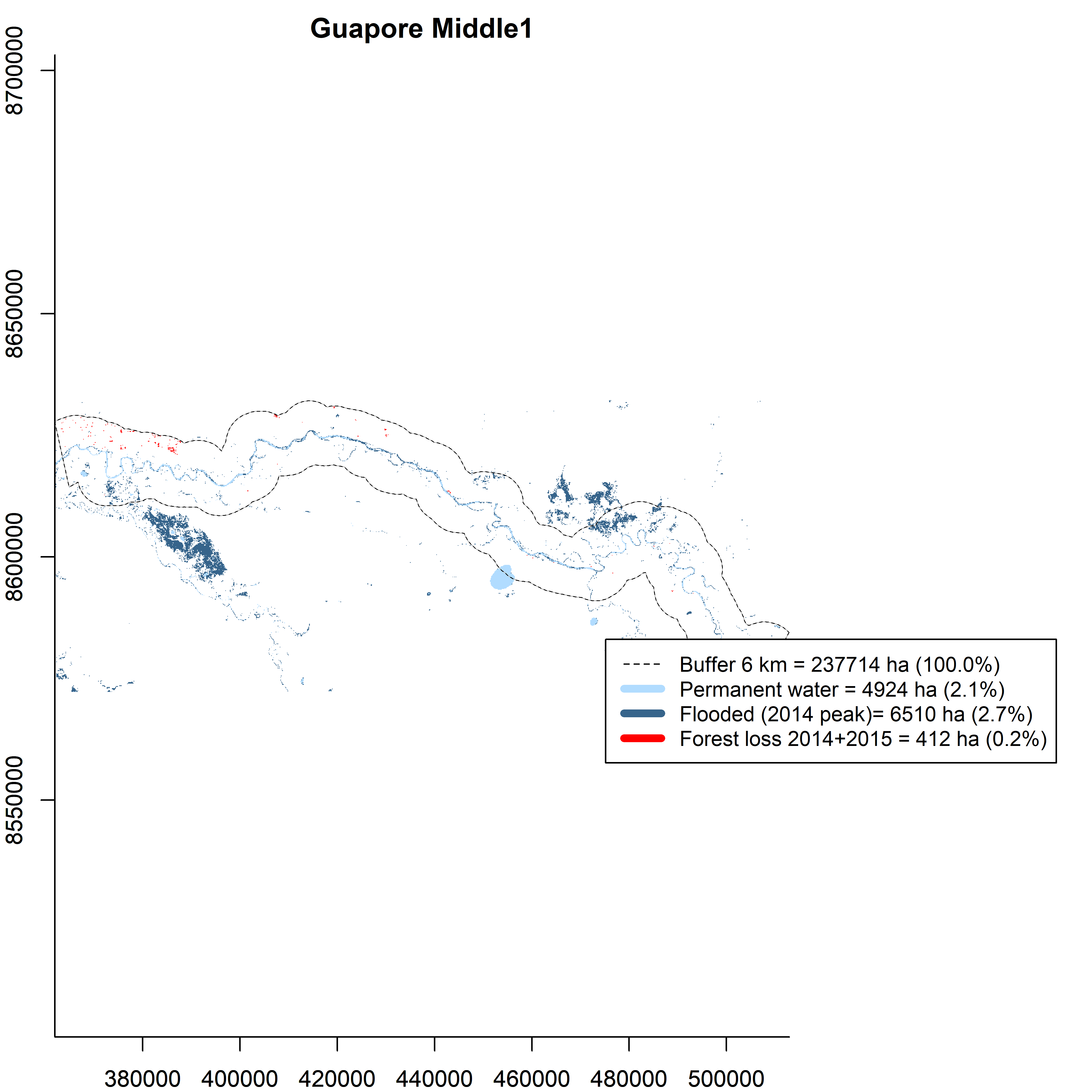

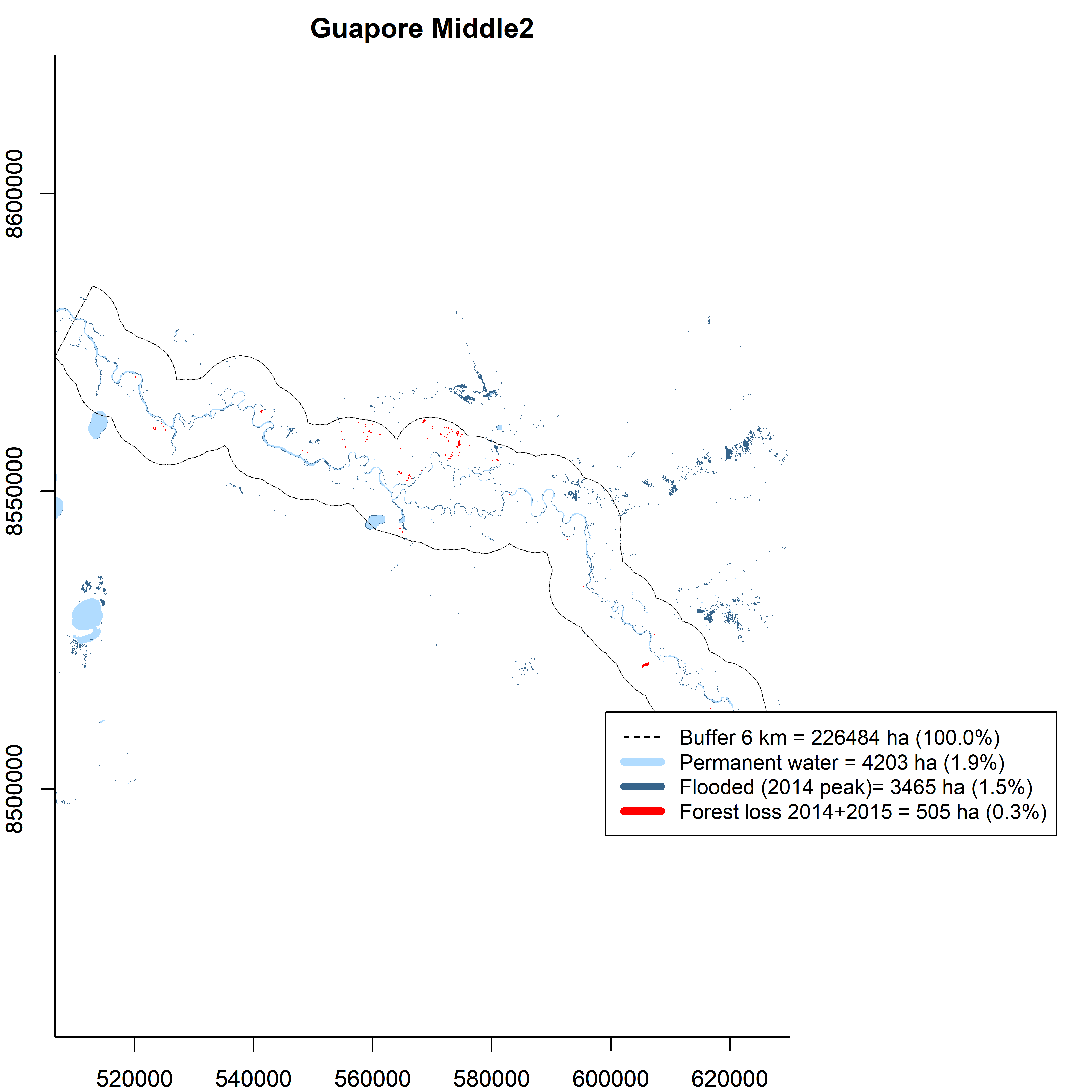

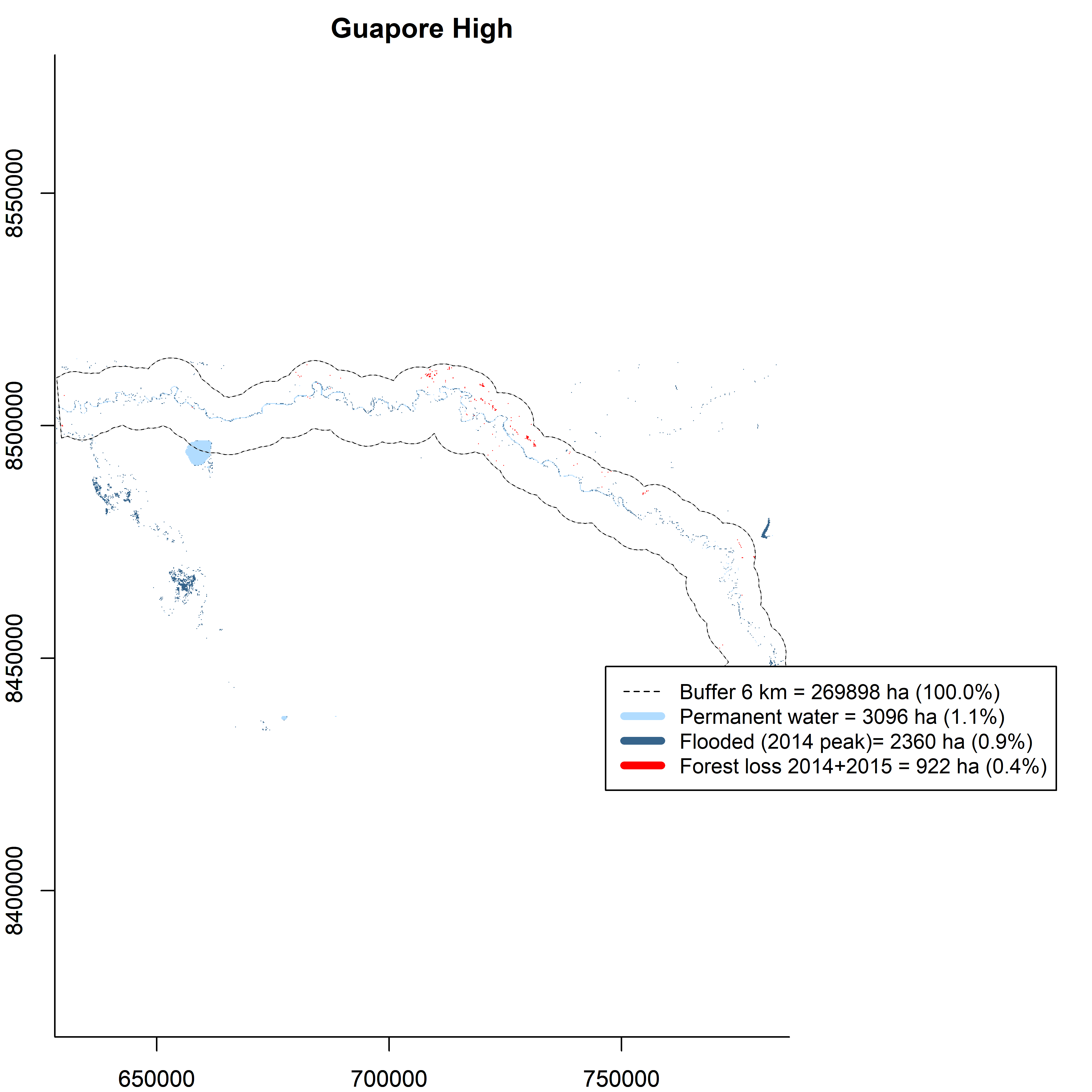

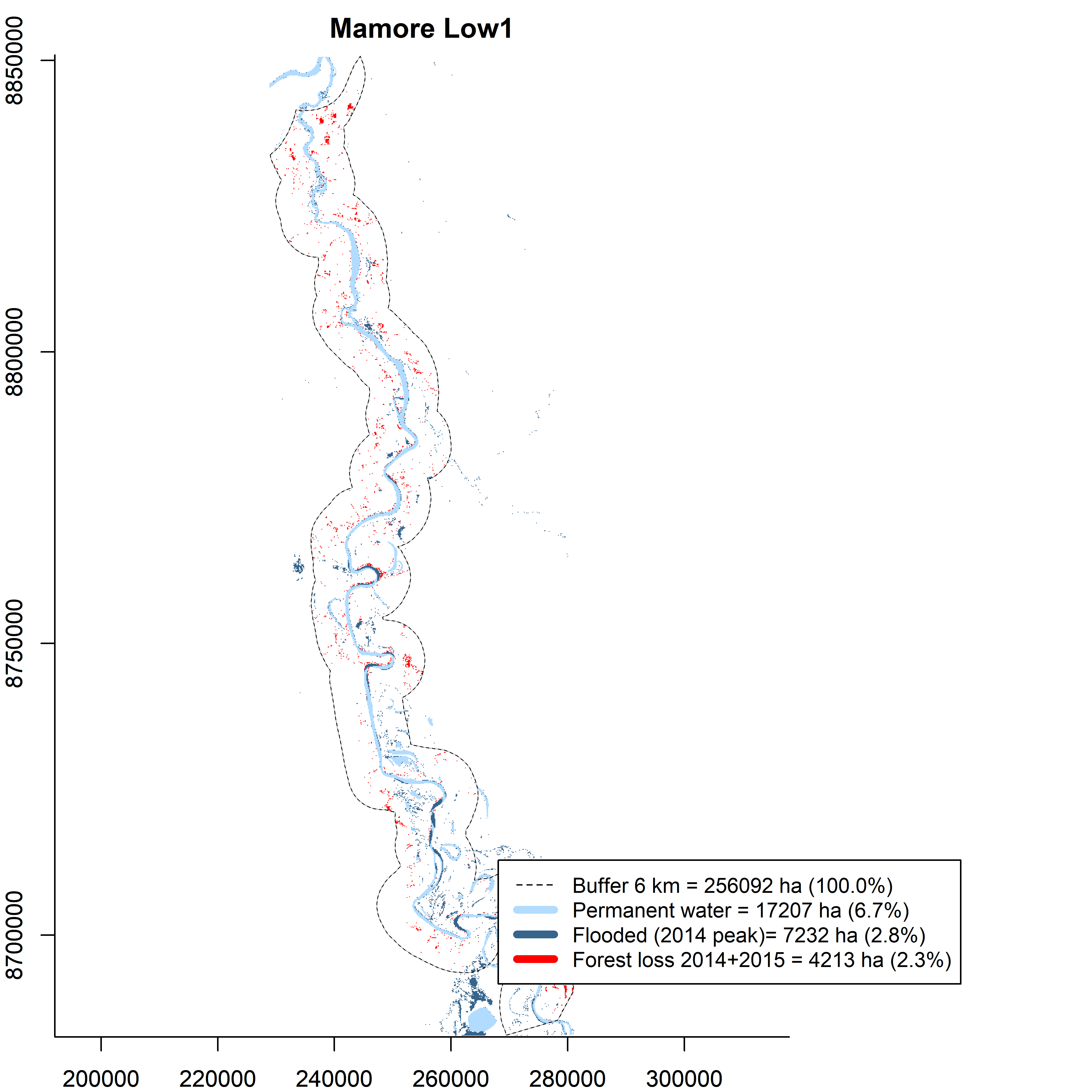

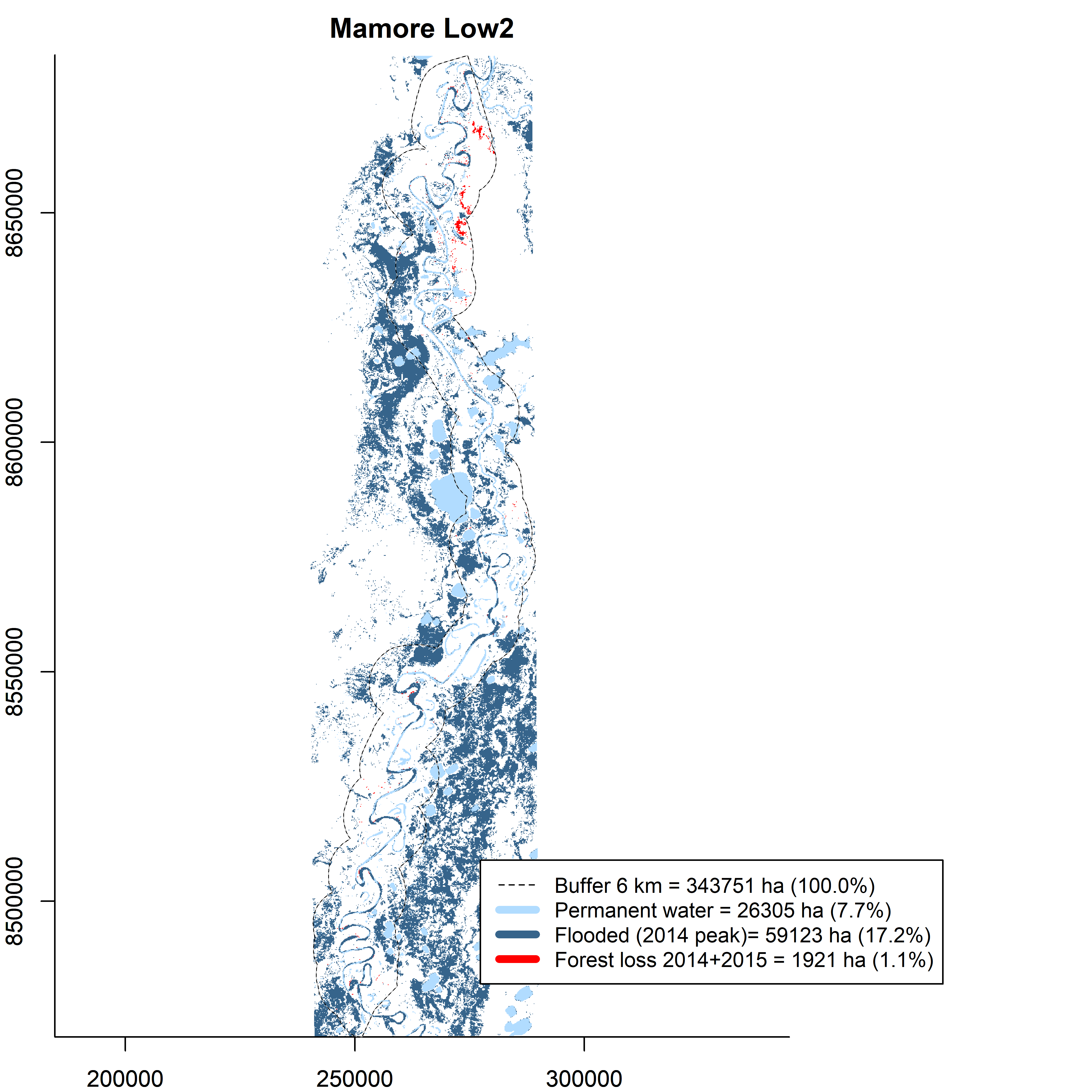

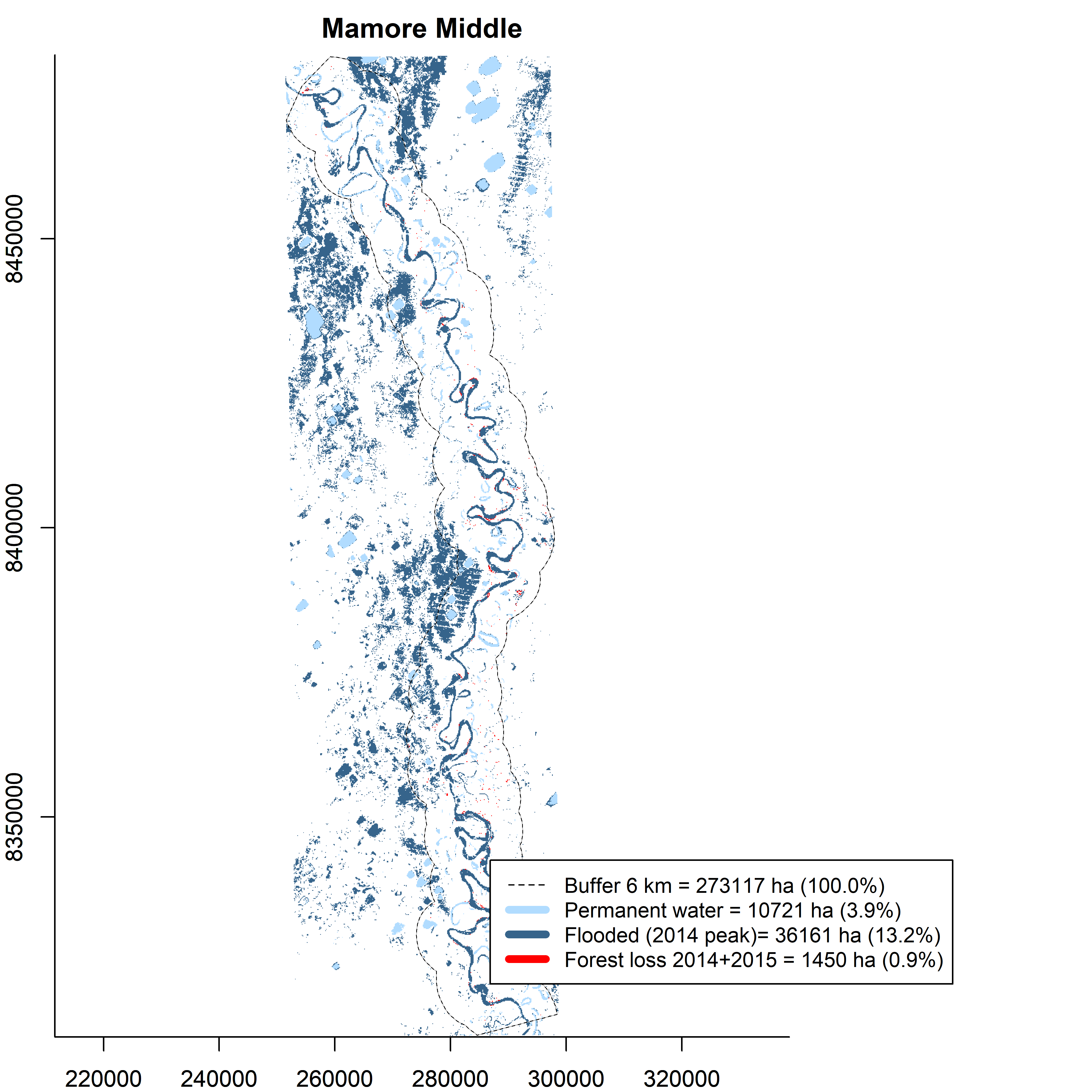

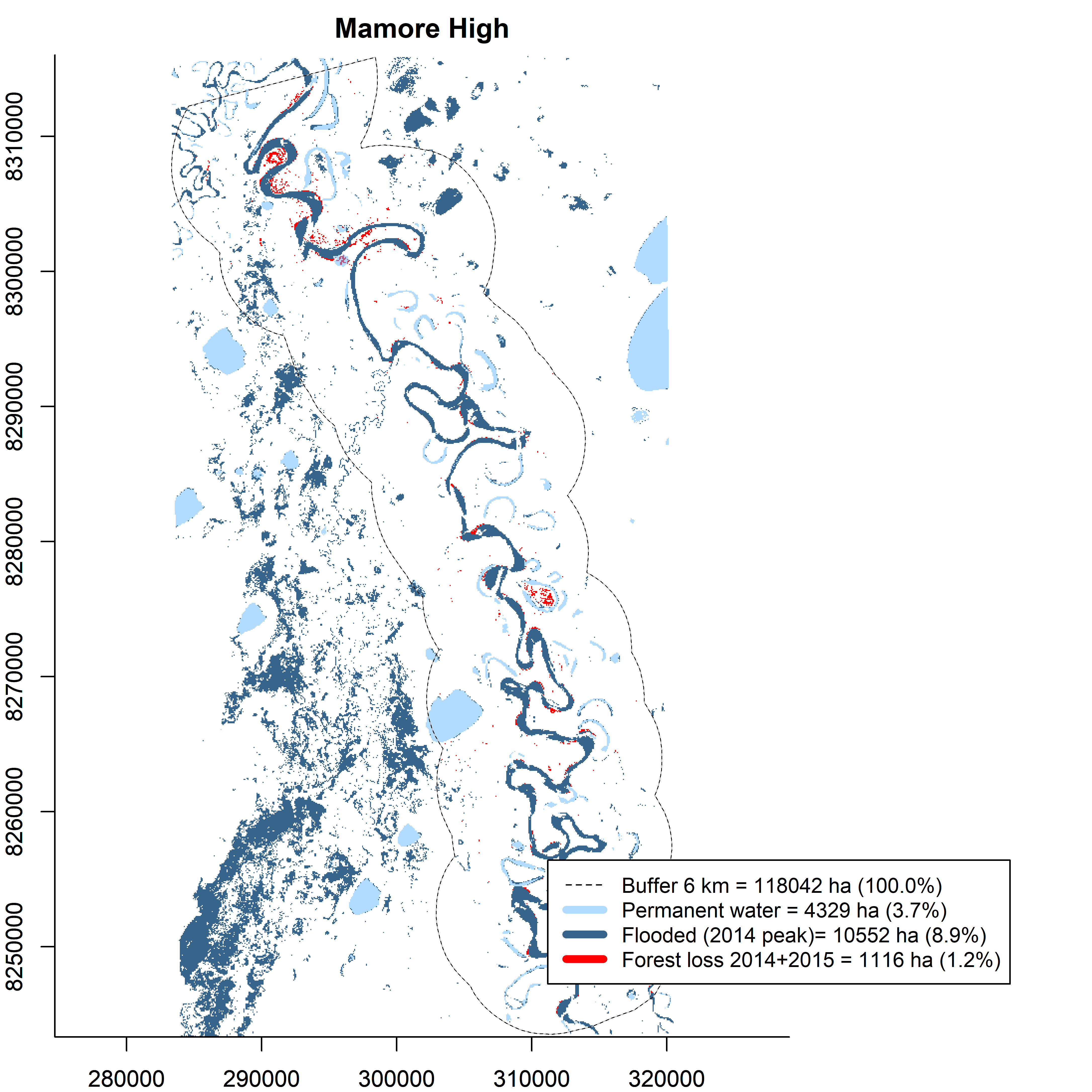

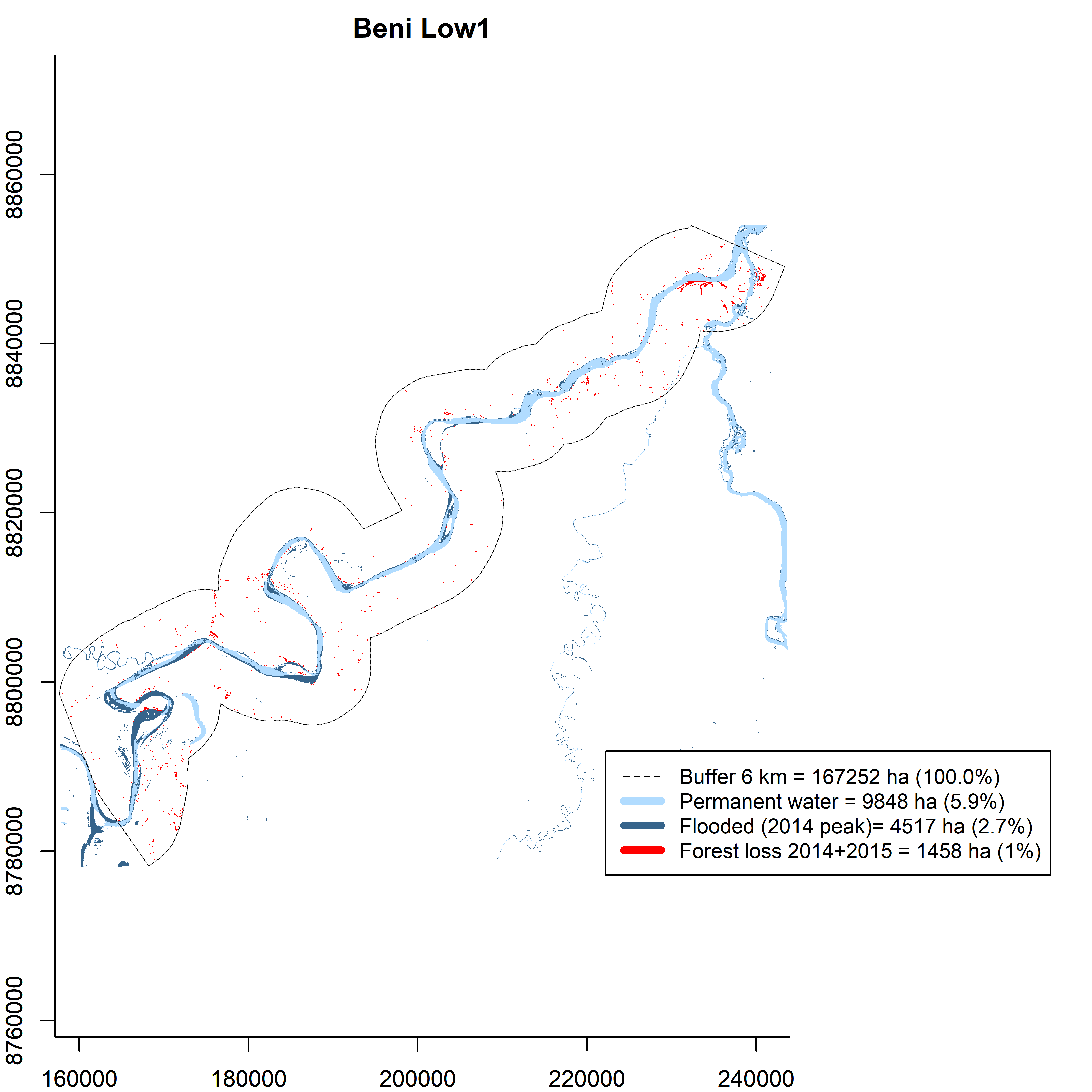

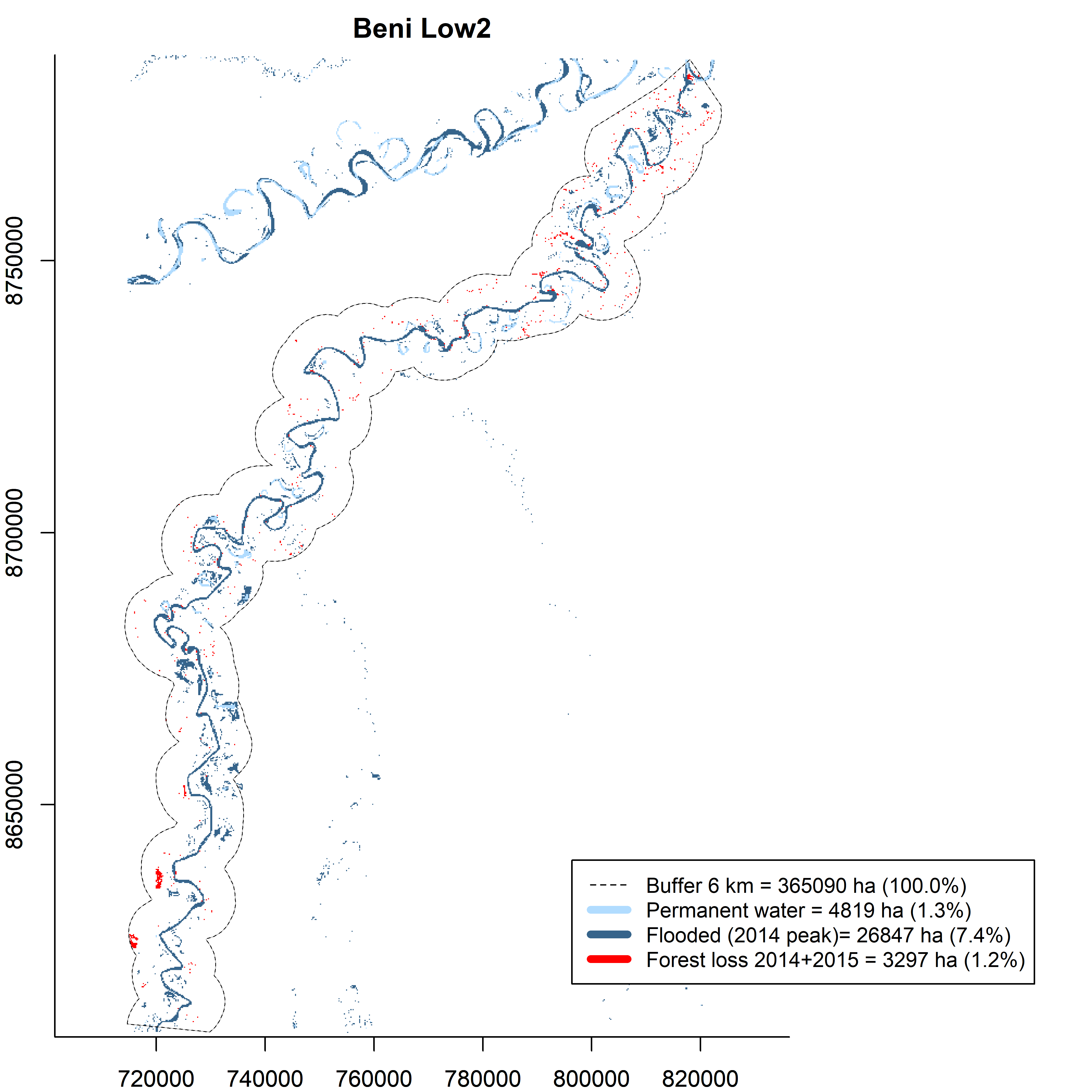

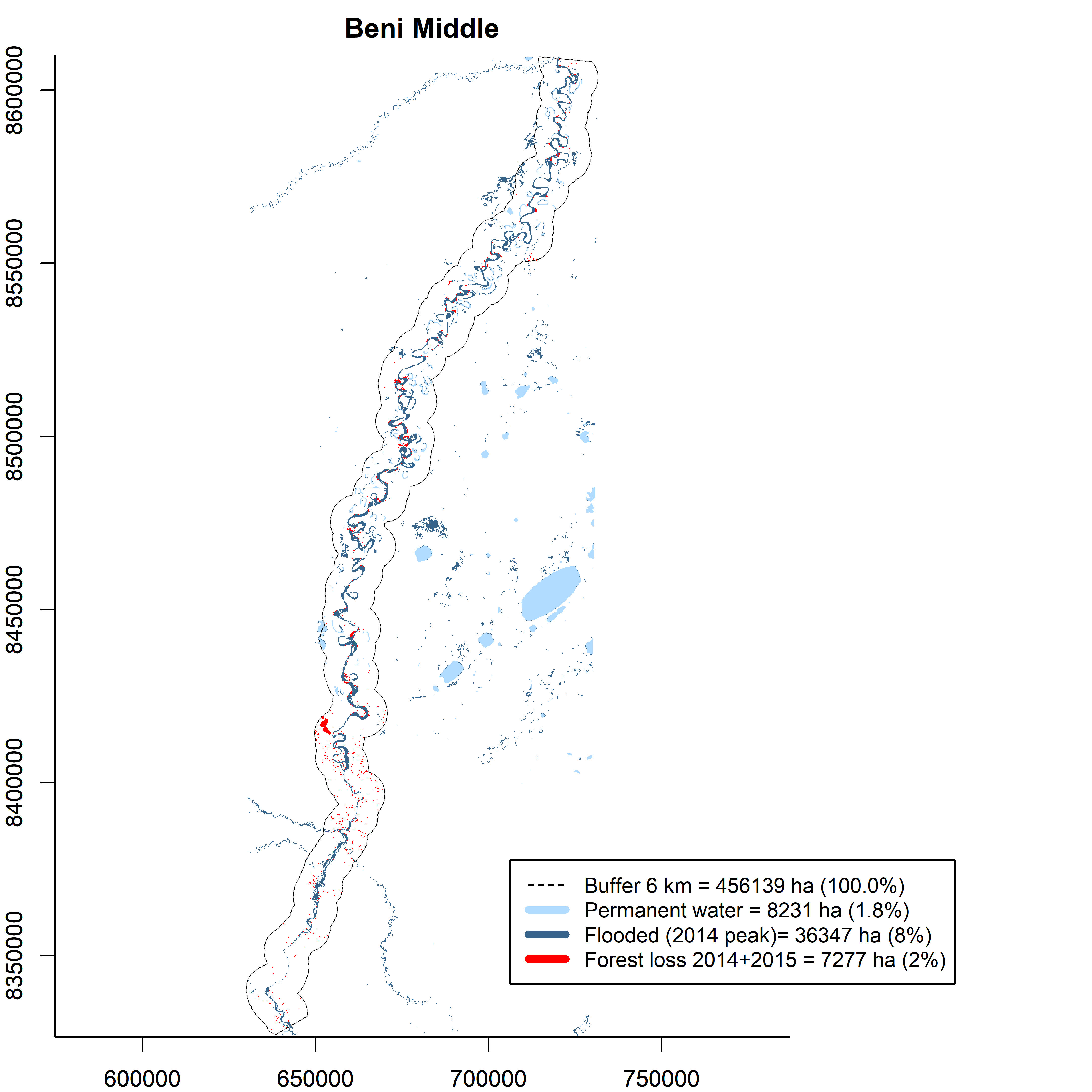

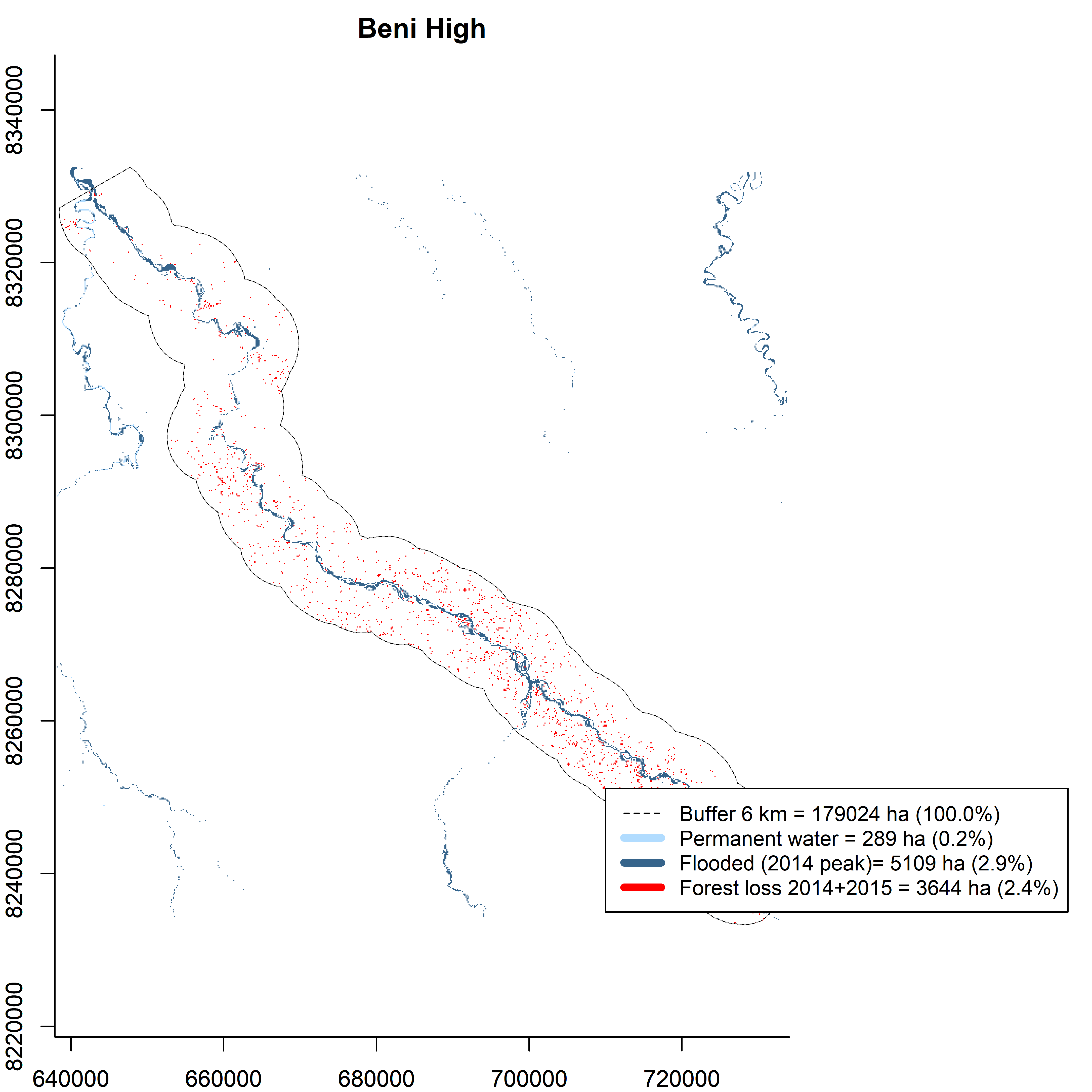

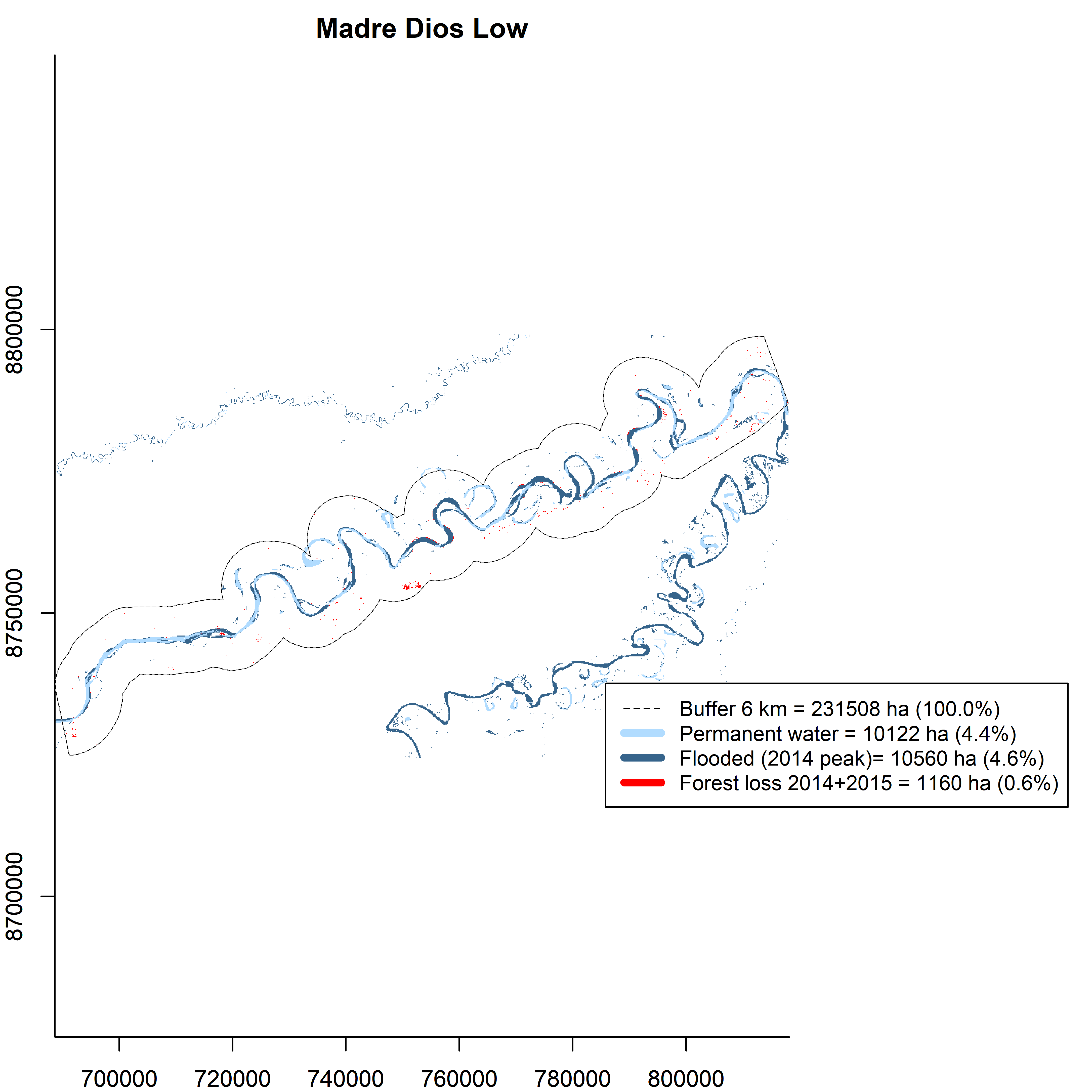

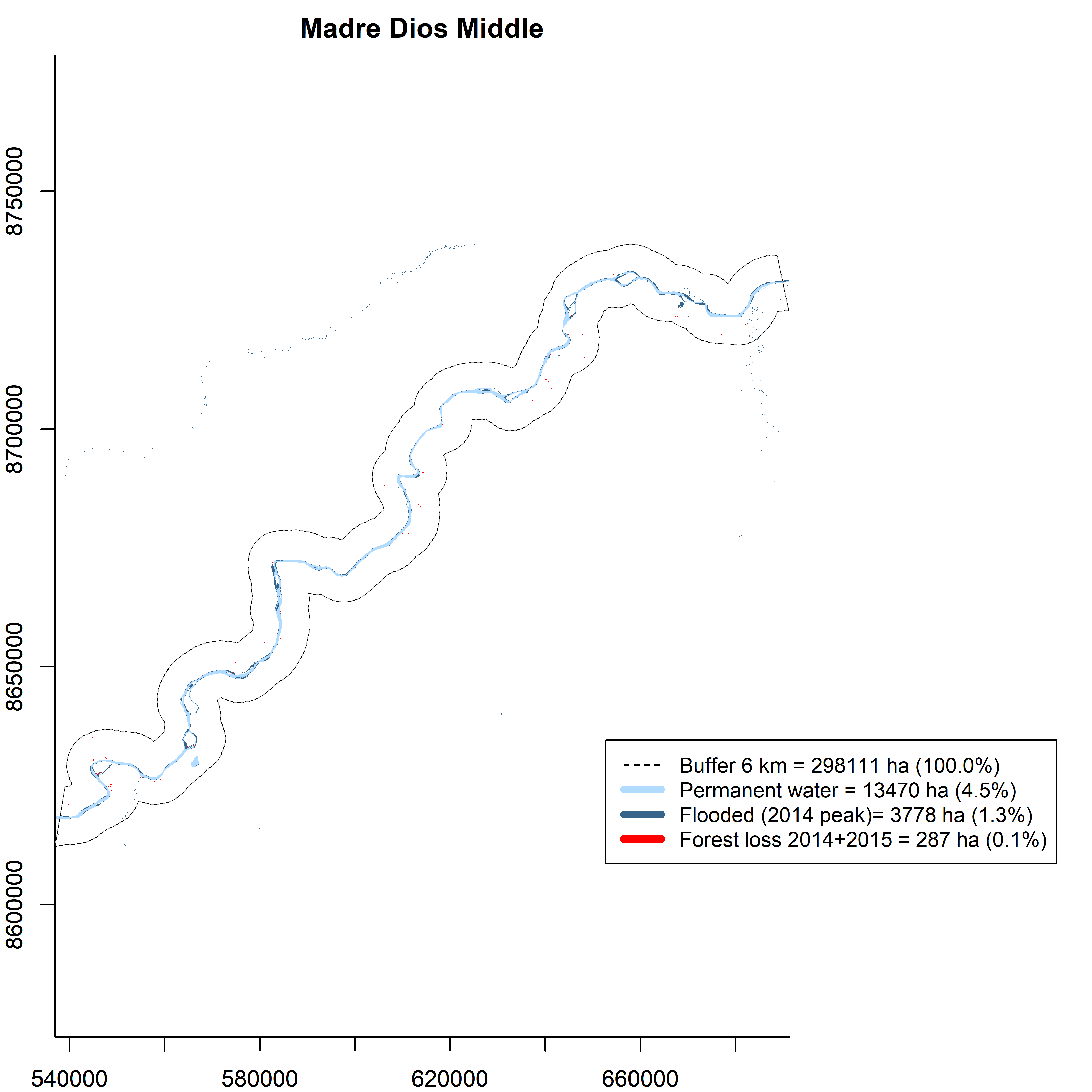

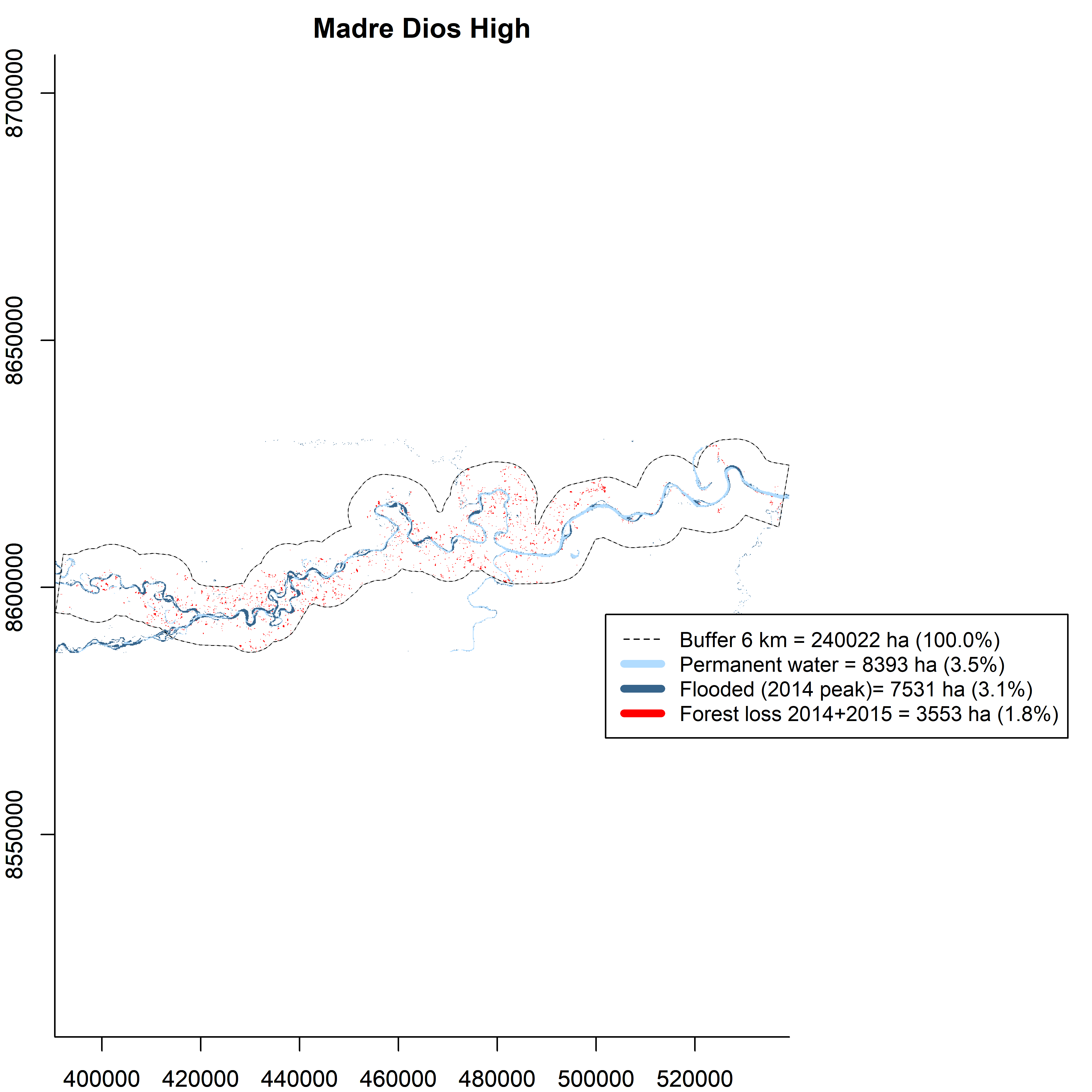

Supplement: S1 Appendix — Scale in UTM coordinates. Diagrams show the 6-km-wide buffer (Buffer) on each bank along the course of the river, percentage of permanent surface water (Permanent water), flooded area at the peak of 2014 extreme flood (Flooded) visible by optical sensors (Landsat), flooded area peak along Jirau reservoir estimated by radar sensor is denoted by the black line (data provided by ESBR), forest loss two years after flooding (2014+2015), highlighting forest loss from filling the reservoirs (yellow) and the loss of forest of unforeseen areas beyond the predicted limits of the reservoirs (red). Deforestation by logging, which was not considered in the calculations of forest loss caused by flood, is shown in purple. Forest loss metrics are presented with the respective percentage of forest loss relative to the area of standing forest in 2013. Locations of dams are indicated by grey bars. Additional analyzed buffers are presented in S1 Appendix. Permanent superficial water and flooded area data from EC JRC/Google. Forest loss data from Hansen/UMD/Google/USGS/NASA. (DOCX) [file pone.0245991.s001.docx]
