# Supplementary material for: Mega-dams and extreme rainfall: Disentangling the drivers of extensive impacts of a large flooding event on Amazon Forests
Source: PLoS One. 2021 Feb 12;16(2):e0245991. doi: 10.1371/journal.pone.0245991 (PMC7880702; doi:10.1371/journal.pone.0245991)
Supplement: S1 Fig — Circles indicate 26 1-ha plots in different habitats: VF = várzea forests, CF = campinarana forests, TF = terra firme forests, DF = transitional forests. Cota 90 represents the initial forecast of flooding by the reservoir, and Flood (2014) represents the peak of extreme flooding as detected by radar/laser sensors (both provided by ESBR). Triangles represent the location of four limnimetric stations along the Jirau reservoir where the level of the Madeira river was measured (see Fig 3). False color composition RGB-654 of Landsat-8/OLI imagery one month after extreme flooding (May/2014), courtesy of the U.S. Geological Survey. (DOCX) [file pone.0245991.s002.docx]

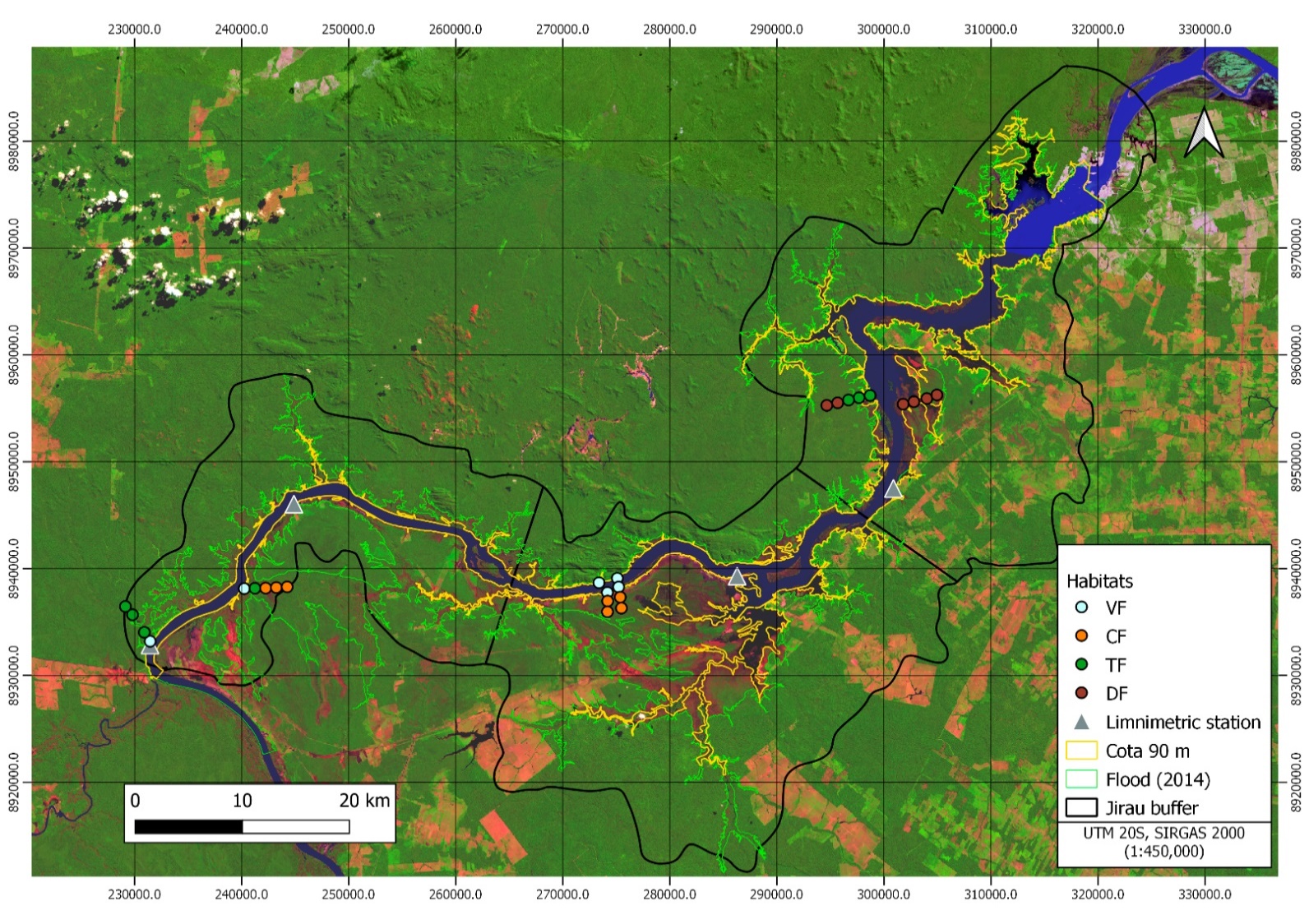


**S1 Fig. Forest monitoring plots along the Jirau reservoir, Rondônia, Brazil.** Circles indicate 26 1-ha plots in different habitats: VF = *várzea* forests, CF = *campinarana* forests, TF = *terra firme* forests, DF = transitional forests. Cota 90 represents the initial forecast of flooding by the reservoir, and Flood (2014) represents the peak of extreme flooding as detected by radar/laser sensors (both provided by ESBR). Triangles represent the location of four limnimetric stations along the Jirau reservoir where the level of the Madeira river was measured (see Figure 3). False color composition RGB-654 of Landsat-8 / OLI imagery one month after extreme flooding (May/2014), courtesy of the U.S. Geological Survey.
