# Supplementary material for: Mega-dams and extreme rainfall: Disentangling the drivers of extensive impacts of a large flooding event on Amazon Forests
Source: PLoS One. 2021 Feb 12;16(2):e0245991. doi: 10.1371/journal.pone.0245991 (PMC7880702; doi:10.1371/journal.pone.0245991)
Supplement: S2 Fig — Estimates include data before (pre-dam) and after (post-dam) the Jirau reservoir filling, in four habitats: terra firme forests (non flooded N = 4; flooded N = 3), transitional forests (non flooded N = 4; flooded N = 2), várzea forests (non flooded N = 1; flooded N = 5), campinarana forests (flooded N = 7), considering different vegetation strata. (a) understory: 1 ≤ DBH < 10 cm; (b) canopy: 10 ≤ DBH <30 cm; (c) emergent DBH ≥ 30 cm. (DOCX) [file pone.0245991.s003.docx]

**
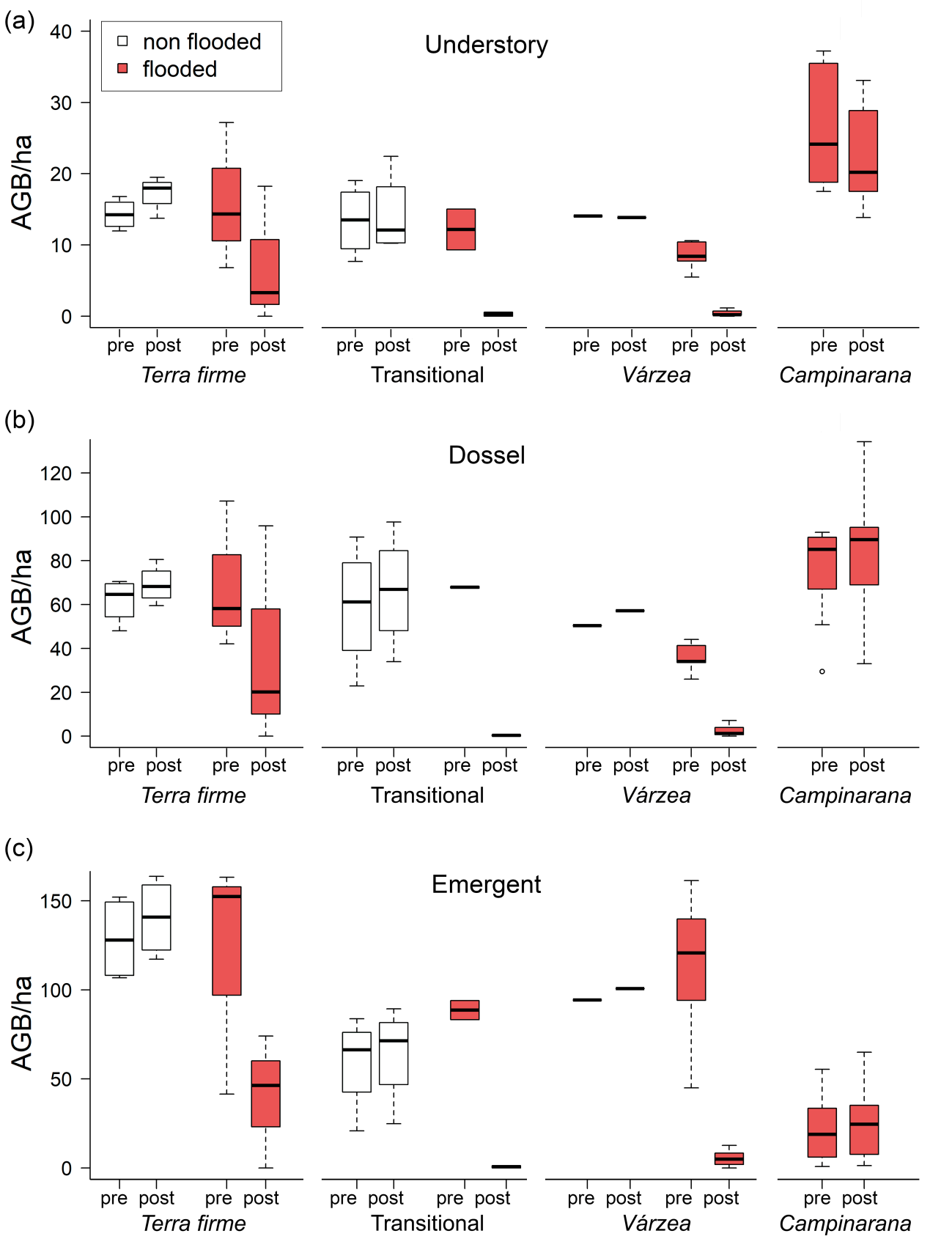
**

**S2 Fig. Estimates of above ground biomass (AGB) per hectare in flooded and non-flooded areas before (pre) and after (post) Jirau reservoir filling.** Estimates include data before (pre-dam) and after (post-dam) the Jirau reservoir filling, in four habitats: *terra firme* forests (non flooded N=4; flooded N=3), transitional forests (non flooded N=4; flooded N=2), *várzea* forests (non flooded N=1; flooded N=5), *campinarana* forests (flooded N=7), considering different vegetation strata. (a) understory: 1 ≤ DBH < 10 cm; (b) canopy: 10 ≤ DBH <30 cm; (c) emergent DBH ≥ 30 cm.
