# Supplementary material for: Mega-dams and extreme rainfall: Disentangling the drivers of extensive impacts of a large flooding event on Amazon Forests
Source: PLoS One. 2021 Feb 12;16(2):e0245991. doi: 10.1371/journal.pone.0245991 (PMC7880702; doi:10.1371/journal.pone.0245991)
Supplement: S1 Table — Total values were calculated from monthly average estimates from the CHIRPS climatic data set at high spatial resolution (0.05° x 0.05°). Area (km2) of each basin is indicated, with the respective number of pixels used to represent the climatic data. (DOCX) [file pone.0245991.s004.docx]

**S1 Table. Total rainfall (mm) during the extreme flood period (September-2013 to August-2014) in the Madeira River sub-basins.** Total values were calculated from monthly average estimates from the CHIRPS climatic data set at high spatial resolution (0.05° x 0.05°). Area (km²) of each basin is indicated, with the respective number of pixels used to represent the climatic data.

| **River Basin** | **Rainfall (mm)** | **Area (km^2^)** | **#Pixels** |
| --- | --- | --- | --- |
| Madre de Dios | 2747.9 | 166333.5 | 5400 |
| Beni | 1985.3 | 133682.8 | 4340 |
| Mamoré | 1749.6 | 292500.5 | 9496 |
| Guaporé | 1476.1 | 363962.3 | 11816 |
| Madeira | 2372.1 | 457848.4 | 14864 |
