# Supplementary material for: Mega-dams and extreme rainfall: Disentangling the drivers of extensive impacts of a large flooding event on Amazon Forests
Source: PLoS One. 2021 Feb 12;16(2):e0245991. doi: 10.1371/journal.pone.0245991 (PMC7880702; doi:10.1371/journal.pone.0245991)
Supplement: S2 Table — The landscape sections were established along the river channel from 6 km wide buffers on each bank and area (km2) is indicated; permanent surface water area (Perennial); 2013–2014 flooded area (Flooded); 2014 and 2015 forest losses (Forest Loss). Calculation of forest loss in the reservoirs of Jirau and Santo Antônio excluded areas submerged by the filling of reservoirs. Therefore, only areas beyond the hydroelectric reservoirs were considered as forest loss. The percentage deforestation in relation to forest cover before flooding in 2013 (Forest cover) is indicated in parentheses. (DOCX) [file pone.0245991.s005.docx]

**S2 Table**. **Landscape metrics in 30 sections in the Madeira River sub-basins.** The landscape sections were established along the river channel from 6 km wide buffers on each bank and area (km^2^) is indicated; permanent surface water area (Perennial); 2013-2014 flooded area (Flooded); 2014 and 2015 forest losses (Forest Loss). Calculation of forest loss in the reservoirs of Jirau and Santo Antônio excluded areas submerged by the filling of reservoirs. Therefore, only areas beyond the hydroelectric reservoirs were considered as forest loss. The percentage deforestation in relation to forest cover before flooding in 2013 (Forest 2013) is indicated in parentheses.

| **River Basin** | **Section** | **Buffer** | **Perennial** | **Flooded** | **Forest 2013** | **Forest Loss** |
| --- | --- | --- | --- | --- | --- | --- |
| Madre de Dios | High | 2400.22 | 83.93 | 75.31 | 1984.1 | 35.53 (1.79%) |
|  | Middle | 2981.11 | 134.7 | 37.78 | 2746.2 | 2.87 (0.1%) |
|  | Low | 2315.08 | 101.22 | 105.6 | 2011.2 | 11.6 (0.58%) |
| Beni | High | 1790.24 | 2.89 | 51.09 | 1513.4 | 36.44 (2.41%) |
|  | Middle | 4561.39 | 82.31 | 363.47 | 3729.5 | 72.77 (1.95%) |
|  | Low1 | 1672.52 | 98.48 | 45.17 | 2847.3 | 14.58 (1.04%) |
|  | Low2 | 3650.9 | 48.19 | 268.47 | 1396.4 | 32.97 (1.16%) |
| Mamore | High | 1180.42 | 43.29 | 105.52 | 918.6 | 11.16 (1.21%) |
|  | Middle | 2731.17 | 107.21 | 361.61 | 1594.0 | 14.5 (0.91%) |
|  | Low1 | 2560.92 | 172.07 | 72.32 | 1745.0 | 42.13 (2.34%) |
|  | Low2 | 3437.51 | 263.05 | 591.23 | 1798.0 | 19.21 (1.1%) |
| Guapore | High | 2698.98 | 30.96 | 23.6 | 2110.7 | 9.22 (0.44%) |
|  | Middle1 | 2264.84 | 42.03 | 34.65 | 1788.8 | 5.05 (0.28%) |
|  | Middle2 | 2377.14 | 49.24 | 65.1 | 1918.5 | 4.12 (0.21%) |
|  | Low | 1717.14 | 74.61 | 123.23 | 1324.8 | 15.34 (1.16%) |
| Madeira | High1 | 480.36 | 31 | 3.2 | 2509.0 | 34.19 (9.36%) |
|  | High2 | 328.8 | 24.23 | 3.25 | 2039.4 | 10.75 (4.78%) |
|  | High3 | 298.4 | 30.32 | 3.83 | 703.7 | 5.17 (3.07%) |
|  | Middle1 | 2272.36 | 213.89 | 98.46 | 1537.9 | 8.72 (0.46%) |
|  | Middle2 | 1399.28 | 157.4 | 49.82 | 1115.2 | 11.74 (1.05%) |
|  | Middle3 | 2037.46 | 199.36 | 99.39 | 1911.9 | 29.26 (1.9%) |
|  | Low1 | 1109.19 | 219.75 | 59.22 | 168.4 | 9.66 (1.37%) |
|  | Low2 | 2601.75 | 402.03 | 52.19 | 225.1 | 20.38 (1%) |
|  | Low3 | 3108.83 | 336.09 | 107.11 | 365.2 | 43.31 (1.73%) |
| Jirau | Initial | 1007.67 | 58.43 | 85.13 | 708.2 | 43.44 (6.13%) |
|  | Middle | 1018.9 | 33.18 | 38 | 800.3 | 92.39 (11.54%) |
|  | Final | 645.96 | 38.21 | 4.36 | 562.6 | 54.29 (9.65%) |
| Santo Antônio | Initial | 524.7 | 52.88 | 46.98 | 254.8 | 3.59 (1.41%) |
|  | Middle | 437.63 | 43.81 | 55.91 | 237.9 | 15.75 (6.62%) |
|  | Final | 384.59 | 34.95 | 31.17 | 260.0 | 36.64 (14.09%) |
