# Supplementary material for: Mega-dams and extreme rainfall: Disentangling the drivers of extensive impacts of a large flooding event on Amazon Forests
Source: PLoS One. 2021 Feb 12;16(2):e0245991. doi: 10.1371/journal.pone.0245991 (PMC7880702; doi:10.1371/journal.pone.0245991)
Supplement: S3 Table — (DOCX) [file pone.0245991.s006.docx]

**S3 Table. Number of individuals of tree species in three diameter size classes in four forest habitats sampled in 26 1-ha plots in the area of influence of the Jirau dam, before (pre - 2011) and after (post - 2015) the filling of the reservoir.**

|  | ***Terra firme*** | |  | **Transitional** | |  | ***Várzea*** | |  | ***Campinarana*** | |
| --- | --- | --- | --- | --- | --- | --- | --- | --- | --- | --- | --- |
|  | pre | post |  | pre | post |  | pre | post |  | pre | post |
| **1 ≤ DBH < 10 cm** | 1255 | 900 |  | 1030 | 578 |  | *721* | *405* |  | 2232 | 1517 |
| **10 ≤ DBH < 30 cm** | 1477 | 1148 |  | 1180 | 826 |  | 930 | 270 |  | 2722 | 2493 |
| **DBH ≥ 30 cm** | 464 | 351 |  | 323 | 181 |  | 444 | 115 |  | 197 | 203 |
