# Supplementary material for: Mega-dams and extreme rainfall: Disentangling the drivers of extensive impacts of a large flooding event on Amazon Forests
Source: PLoS One. 2021 Feb 12;16(2):e0245991. doi: 10.1371/journal.pone.0245991 (PMC7880702; doi:10.1371/journal.pone.0245991)
Supplement: S4 Table — DBH(max) = average maximum individual diameter per plot (cm); DBH = average diameter at breast height (cm); H(max) = average maximum height per plot (m); H = average height (m); AB(max) = average maximum individual basal area per plot (m2); AB = average individual basal area (m2); WD = average wood density (g cm−3); AGB(max) = average maximum individual aboveground biomass per plot (Mg); AGB = average individual aboveground biomass per plot (Mg); Abundance (min, max) = minimum and maximum individuals per plot; Abundance_plot = average individuals per plot; Fisher’s alpha = diversity index; Singletons (min, max) = minimum and maximum species per plot with only one individual per species; Singletons = average species with only one occurrence record per plot; Doubletons (min, max) = minimum and maximum species with only two individuals per plot; Doubletons = average species with only two individuals per plot. Species = average species per plot; Genus = average genera per plot; Family = average families per plot; Total number (#) of species, genera and families in each habitat; (*) values represent minimum and maximum. The number of plots in each habitat is indicated by (n). (DOCX) [file pone.0245991.s007.docx]

**S4 Table. Descriptive statistics (mean ± standard deviation) of diversity metrics and structural variables in 17 plots affected by flooding in four habitats before (2011) and after (2015) the filling of the Jirau reservoir.** DBH(max)= average maximum individual diameter per plot (cm); DBH= average diameter at breast height (cm); H(max)= average maximum height per plot (m); H= average height (m); AB(max)= average maximum individual basal area per plot (m²); AB= average individual basal area (m²); WD= average wood density (g cm−3); AGB(max)= average maximum individual aboveground biomass per plot (Mg); AGB = average individual aboveground biomass per plot (Mg); Abundance (min, max) = minimum and maximum individuals per plot; Abundance_plot = average individuals per plot; Fisher’s alpha = diversity index; Singletons (min, max) = minimum and maximum species per plot with only one individual per species; Singletons = average species with only one occurrence record per plot; Doubletons (min, max) = minimum and maximum species with only two individuals per plot; Doubletons = average species with only two individuals per plot. Species = average species per plot; Genus = average genera per plot; Family = average families per plot; Total number (#) of species, genera and families in each habitat; (*) values represent minimum and maximum. The number of plots in each habitat is indicated by (n).

|  | ***Terra firme* forests**  **(n=3)** | | | | **Transitional forests**  **(n=2)** | | | | ***Várzea* forests**  **(n=5)** | | | | ***Campinarana forests***  **(n=7)** | | | |
| --- | --- | --- | --- | --- | --- | --- | --- | --- | --- | --- | --- | --- | --- | --- | --- | --- |
|  | 2011 | | 2015 | | 2011 | | 2015 | | 2011 | | 2015 | | 2011 | | 2015 | |
|  | ***mean*** | *sd* | ***mean*** | *sd* | ***mean*** | *sd* | ***mean*** | *sd* | ***mean*** | *sd* | ***mean*** | *sd* | ***mean*** | *sd* | ***mean*** | *sd* |
| *DBH_(max)_* | **98.20** | 32.66 | **76.50** | 17.39 | **69.45** | 18.60 | **43.00** | NA | **109.45** | 19.37 | **44.38** | 4.72 | **52.66** | 17.00 | **54.20** | 17.53 |
| *DBH* | **15.20** | 14.20 | **17.02** | 12.13 | **13.96** | 13.26 | **11.10** | 11.42 | **19.09** | 17.44 | **10.55** | 13.40 | **10.88** | 8.61 | **12.25** | 8.80 |
| *H_(max)_* | **38.33** | 7.23 | **33.00** | 4.24 | **32.00** | 0.00 | **20.00** | NA | **37.00** | 2.74 | **29.63** | 4.96 | **22.29** | 8.42 | **23.79** | 8.18 |
| *H* | **11.59** | 7.01 | **13.21** | 6.64 | **10.35** | 6.87 | **7.46** | 7.05 | **11.14** | 7.31 | **6.79** | 8.10 | **8.38** | 4.97 | **9.44** | 5.33 |
| *AB_(max)_* | **0.81** | 0.46 | **0.47** | 0.21 | **0.39** | 0.20 | **0.15** | NA | **0.96** | 0.34 | **0.16** | 0.03 | **0.24** | 0.15 | **0.25** | 0.16 |
| *AB* | **0.03** | 0.08 | **0.03** | 0.05 | **0.03** | 0.05 | **0.02** | 0.04 | **0.05** | 0.10 | **0.02** | 0.04 | **0.02** | 0.02 | **0.02** | 0.03 |
| *WoodDens* | **0.67** | 0.16 | **0.72** | 0.17 | **0.65** | 0.13 | **0.67** | 0.15 | **0.60** | 0.17 | **0.36** | 0.16 | **0.64** | 0.10 | **0.64** | 0.11 |
| *AGB_(max)_* | **13.00** | 8.49 | **6.61** | 3.69 | **5.68** | 2.22 | **1.37** | NA | **9.46** | 4.84 | **1.20** | 1.12 | **2.02** | 1.62 | **2.52** | 2.03 |
| *AGB* | **0.34** | 1.07 | **0.35** | 0.79 | **0.25** | 0.55 | **0.16** | 0.40 | **0.41** | 1.06 | **0.11** | 0.28 | **0.09** | 0.20 | **0.11** | 0.25 |
| *Abundance_(min, max)*_* | **378.00** | 586.00 | **0.00** | 401.00 | **436.00** | 539.00 | **0.00** | 11.00 | **220.00** | 379.00 | **0.00** | 137.00 | **542.00** | 944.00 | **402.00** | 804.00 |
| *Abundance/plot* | **455.67** | 113.56 | **170.33** | 207.20 | **487.50** | 72.83 | **5.50** | 7.78 | **321.40** | 63.26 | **61.60** | 65.63 | **735.86** | 162.66 | **601.86** | 140.05 |
| *alpha* | **104.39** | 50.80 | **22.09** | 19.78 | **63.38** | 13.36 | **27.14** | 36.97 | **60.49** | 15.26 | **4.47** | 3.66 | **15.81** | 7.07 | **13.13** | 6.72 |
| *Singletons_(min, max)*_* | **70.00** | 101.00 | **0.00** | 58.00 | **46.00** | 82.00 | **0.00** | 10.00 | **25.00** | 50.00 | **0.00** | 19.00 | **6.00** | 37.00 | **6.00** | 43.00 |
| *Singletons* | **87.33** | 15.82 | **32.67** | 29.69 | **64.00** | 25.46 | **5.00** | 7.07 | **37.60** | 10.64 | **6.60** | 8.05 | **13.86** | 10.70 | **16.14** | 12.52 |
| *Doubletons_(min, max)*_* | **25.00** | 63.00 | **0.00** | 13.00 | **73.00** | 73.00 | **0.00** | 0.00 | **16.00** | 40.00 | **0.00** | 7.00 | **8.00** | 25.00 | **0.00** | 14.00 |
| *Doubletons* | **48.67** | 20.65 | **8.67** | 7.51 | **73.00** | 0.00 | **0.00** | 0.00 | **30.80** | 9.96 | **3.20** | 2.49 | **13.43** | 6.29 | **8.29** | 4.39 |
| *Species* | **162.00** | 36.29 | **41.33** | 36.91 | **137.00** | 25.46 | **5.00** | 7.07 | **111.20** | 24.75 | **11.00** | 10.86 | **58.86** | 18.11 | **48.71** | 17.39 |
| *Genus* | **102.33** | 21.08 | **30.00** | 26.06 | **84.00** | 18.38 | **4.50** | 6.36 | **79.00** | 15.36 | **10.20** | 9.76 | **47.29** | 9.98 | **39.00** | 9.50 |
| *Family* | **40.67** | 3.21 | **13.33** | 12.22 | **40.00** | 1.41 | **4.00** | 5.66 | **34.80** | 5.50 | **8.00** | 7.18 | **28.14** | 3.08 | **23.14** | 3.63 |
| *# Species* | **361.00** | - | **111.00** | - | **201.00** | - | **10.00** | - | **326.00** | - | **43.00** | - | **195.00** | - | **183.00** | - |
| *# Genus* | **179.00** | - | **70.00** | - | **114.00** | - | **9.00** | - | **172.00** | - | **38.00** | - | **115.00** | - | **108.00** | - |
| *# Family* | **57.00** | - | **27.00** | - | **48.00** | - | **8.00** | - | **57.00** | - | **20.00** | - | **49.00** | - | **44.00** | - |
