# Supplementary material for: Mega-dams and extreme rainfall: Disentangling the drivers of extensive impacts of a large flooding event on Amazon Forests
Source: PLoS One. 2021 Feb 12;16(2):e0245991. doi: 10.1371/journal.pone.0245991 (PMC7880702; doi:10.1371/journal.pone.0245991)
Supplement: S5 Table — DBH(max) = average maximum individual diameter per plot (cm); DBH = average diameter at breast height (cm); H(max) = average maximum height per plot (m); H = average height (m); AB(max) = average maximum individual basal area per plot (m2); AB = average individual basal area (m2); WD = average wood density (g cm−3); AGB(max) = average maximum individual aboveground biomass per plot (Mg); AGB = average individual aboveground biomass per plot (Mg); Abundance (min, max) = minimum and maximum individuals per plot; Abundance_plot = average individuals per plot; Fisher’s alpha = diversity index; Singletons (min, max) = minimum and maximum species per plot with only one individual per species; Singletons = average species with only one occurrence record per plot; Doubletons (min, max) = minimum and maximum species with only two individuals per plot; Doubletons = average species with only two individuals per plot. Species = average species per plot; Genus = average genera per plot; Family = average families per plot; Total number (#) of species, genera and families in each habitat; (*) values represent minimum and maximum. The number of plots in each habitat is indicated by (n). (DOCX) [file pone.0245991.s008.docx]

**S5 Table. Descriptive statistics (mean ± standard deviation) of diversity metrics and structural variables in 9 plots not impacted by the 2014 flooding, before (2011) and after (2015) the filling of the Jirau reservoir.** DBH(max)= average maximum individual diameter per plot (cm); DBH= average diameter at breast height (cm); H(max)= average maximum height per plot (m); H= average height (m); AB(max)= average maximum individual basal area per plot (m²); AB= average individual basal area (m²); WD= average wood density (g cm−3); AGB(max)= average maximum individual aboveground biomass per plot (Mg); AGB = average individual aboveground biomass per plot (Mg); Abundance (min, max) = minimum and maximum individuals per plot; Abundance_plot = average individuals per plot; Fisher’s alpha = diversity index; Singletons (min, max) = minimum and maximum species per plot with only one individual per species; Singletons = average species with only one occurrence record per plot; Doubletons (min, max) = minimum and maximum species with only two individuals per plot; Doubletons = average species with only two individuals per plot. Species = average species per plot; Genus = average genera per plot; Family = average families per plot; Total number (#) of species, genera and families in each habitat; (*) values represent minimum and maximum. The number of plots in each habitat is indicated by (n).

|  | ***Terra firme* forests (n=4)** | | | | **Transitional forests (n=4)** | | | | ***Várzea* forests (n=1)** | | | |
| --- | --- | --- | --- | --- | --- | --- | --- | --- | --- | --- | --- | --- |
|  | 2011 | | 2015 | | 2011 | | 2015 | | 2011 | | 2015 | |
|  | ***mean*** | *sd* | ***mean*** | *sd* | ***mean*** | *sd* | ***mean*** | *sd* | ***mean*** | *sd* | ***mean*** | *sd* |
| *DBH_(max)_* | **98.23** | 8.35 | **100.21** | 8.91 | **60.65** | 9.14 | **62.43** | 9.47 | **140.00** | - | **143.50** | - |
| *DBH* | **14.98** | 14.81 | **15.05** | 14.85 | **14.07** | 12.18 | **14.29** | 12.15 | **14.33** | 14.97 | **14.57** | 15.23 |
| *H_(max)_* | **44.00** | 3.65 | **43.00** | 5.35 | **30.00** | 4.32 | **29.75** | 3.30 | **40.00** | - | **40.00** | - |
| *H* | **11.52** | 7.80 | **12.09** | 8.21 | **10.82** | 6.52 | **11.71** | 6.88 | **9.91** | 7.28 | **10.30** | 7.37 |
| *AB_(max)_* | **0.76** | 0.13 | **0.79** | 0.14 | **0.29** | 0.08 | **0.31** | 0.09 | **1.54** | - | **1.62** | - |
| *AB* | **0.03** | 0.07 | **0.04** | 0.08 | **0.03** | 0.04 | **0.03** | 0.05 | **0.03** | 0.10 | **0.03** | 0.11 |
| *WoodDens* | **0.65** | 0.15 | **0.65** | 0.15 | **0.65** | 0.13 | **0.65** | 0.13 | **0.61** | 0.15 | **0.62** | 0.15 |
| *AGB_(max)_* | **13.50** | 3.64 | **14.54** | 3.36 | **3.70** | 1.38 | **4.06** | 1.29 | **12.65** | - | **13.95** | - |
| *AGB* | **0.35** | 1.07 | **0.37** | 1.13 | **0.23** | 0.49 | **0.25** | 0.53 | **0.25** | 1.01 | **0.27** | 1.10 |
| *Abundance_(min, max)*_* | **386.00** | 531.00 | **390.00** | 547.00 | **245.00** | 501.00 | **272.00** | 471.00 | **488.00** | 488.00 | **482.00** | 482.00 |
| *Abundance/plot* | **457.25** | 75.82 | **472.00** | 73.79 | **389.50** | 110.45 | **393.50** | 93.54 | **488.00** | - | **482.00** | - |
| *alpha* | **103.20** | 18.98 | **107.98** | 22.94 | **62.33** | 19.97 | **63.14** | 21.02 | **92.61** | - | **99.86** | - |
| *Singletons_(min, max)*_* | **49.00** | 93.00 | **49.00** | 92.00 | **26.00** | 54.00 | **27.00** | 51.00 | **85.00** | 85.00 | **92.00** | 92.00 |
| *Singletons* | **64.75** | 19.70 | **67.50** | 18.66 | **43.50** | 12.12 | **43.25** | 11.15 | **85.00** | - | **92.00** | **-** |
| *Doubletons_(min, max)*_* | **36.00** | 54.00 | **37.00** | 53.00 | **25.00** | 63.00 | **28.00** | 64.00 | **37.00** | 37.00 | **34.00** | 34.00 |
| *Doubletons* | **45.00** | 8.83 | **45.00** | 8.68 | **43.50** | 17.54 | **45.50** | 16.82 | **37.00** | - | **34.00** | - |
| *Species* | **172.50** | 16.42 | **178.50** | 14.27 | **121.50** | 29.78 | **123.25** | 30.19 | **170.00** | - | **176.00** | - |
| *Genus* | **109.25** | 7.50 | **112.75** | 7.68 | **80.25** | 17.84 | **78.25** | 17.35 | **120.00** | - | **121.00** | - |
| *Family* | **41.25** | 2.50 | **40.75** | 2.22 | **36.50** | 3.11 | **35.75** | 3.86 | **41.00** | - | **42.00** | - |
| *# Species* | **425.00** | - | **440.00** | - | **302.00** | - | **306.00** | - | **170.00** | - | **176.00** | - |
| *# Genus* | **199.00** | - | **206.00** | - | **153.00** | - | **153.00** | - | **120.00** | - | **121.00** | - |
| *# Family* | **57.00** | - | **56.00** | - | **56.00** | - | **56.00** | - | **41.00** | - | **42.00** | - |
